# Improved simulated ventilation with a novel tidal volume and peak inspiratory pressure controlling bag valve mask: A pilot study

**DOI:** 10.1016/j.resplu.2022.100350

**Published:** 2023-01-05

**Authors:** Jonathan G. Merrell, Adam C. Scott, Ryan Stambro, Amit Boukai, Dylan D. Cooper

**Affiliations:** a Department of Pediatrics, Indiana University School of Medicine, 340 West 10th Street, Suite 6200, Indianapolis, IN, USA; b Compact Medical Inc, 7711 Ashtree Dr., Indianapolis, IN, USA; c The Simulation Center at Fairbanks Hall, Indiana University Health, 340 W Tenth Street, Suite 4100, Indianapolis, IN, USA; d Department of Emergency Medicine, Aventura Hospital and Medical Center, 20900 Biscayne Blvd, Aventura, FL, USA; e Department of Emergency Medicine, Indiana University School of Medicine, Indianapolis, IN, USA

**Keywords:** CPR, Resuscitation, Pediatric, Bag Valve Mask, Ambu, Ventilation, Tidal volume, Peak Inspiratory Pressure, Hyperventilation

## Abstract

**Introduction:**

The dangers of hyperventilation during resuscitation are well known. Traditional bag valve mask (BVM) devices rely on end users to control tidal volume (V_t_), rate, and peak inspiratory pressures (PIP) of ventilation. The Butterfly BVM (BBVM) is a novel device intending to give greater control over these parameters. The objective of this pilot study was to compare the BBVM against a traditional device in simulated resuscitations.

**Methods:**

Senior emergency medicine residents and fellows participated in a three-phase simulation study. First, participants used the Ambu Spur II BVM in adult and pediatric resuscitations. V_t_, PIP, and rate were recorded. Second, participants repeated the resuscitations after a brief introduction to the BBVM. Third, participants were given a longer introduction to the BBVM and were tested on their ability to adjust its various settings.

**Results:**

Nineteen participants were included in the adult arm of the study, and 16 in the pediatric arm. The BBVM restricted V_t_ delivered to a range of 4–8 ml/kg vs 9 ml/kg and 13 ml/kg (Ambu adult and Ambu pediatric respectively). The BBVM never exceeded target minute ventilations while the Ambu BVMs exceeded target minute ventilation in 2 of 4 tests. The BBVM failed to reliably reach higher PIP targets in one test, while the pediatric Ambu device had 76 failures of excessive PIP compared to 2 failures by the BBVM.

**Conclusion:**

The BBVM exceeded the Ambu Spur II in delivering appropriate V_t_s and in keeping PIPs below target maximums to simulated adult and pediatric patients in this pilot study.

## Highlights

• Hyperventilation during resuscitation can be dangerous to patients.
• This study of emergency medicine (EM) residents using traditional Bag Valve Masks (BVMs) showed high tidal volume (V_t_), pressures, and minute ventilations with a traditional BVM in simulated pediatric and adult patients.
• The novel Butterfly BVM generally demonstrated more accurate and appropriate V_t_, pressures, and minute ventilations in this study.
• EM residents found the Butterfly BVM easy to use and preferred it to the traditional BVM.

## Introduction

The bag valve mask (BVM) is one of the most important and common pieces of lifesaving equipment, and this product has formed the backbone of manual ventilatory support for almost 65 years. However, BVMs have also been linked to injuries such as gastric insufflation, aspiration, and pneumothorax from hyperventilation.^1^, ^2^, ^3^ This problem has prompted resuscitation leaders to repeatedly stress the need to “avoid excessive ventilation.”^4^, ^5^, ^6^, ^7^, ^8^ Despite this, studies have shown that airway managers, including those with high levels of experience are prone to hyperventilate patients.^9^, ^10^, ^11^

Traditional BVMs hold a maximum volume of air that is well above physiologic values.^12^ This is a necessary feature for these devices which rely on wall tension for self-inflation. As a result, traditional BVMs primarily rely upon a user’s skill to ensure proper tidal volume (V_t_) delivery. Expecting an airway manager to simultaneously monitor V_t_, peak inspiratory pressure (PIP), inspiratory rate, and mask seal while watching for chest rise/fall during a code scenario has proven to be a nearly impossible task.^9^, ^10^, ^11^ To reduce the risk of hyperventilation, some thought leaders now call for airway managers to consider using the pediatric BVM as first-line therapy for adults.^13^, ^14^, ^15^ Other solutions such as timing lights or metronomes have proven effective at promoting more responsible rates,^16^, ^17^ but BVMs with these accessories have yet to reach mainstream.

**Fig. 1:**
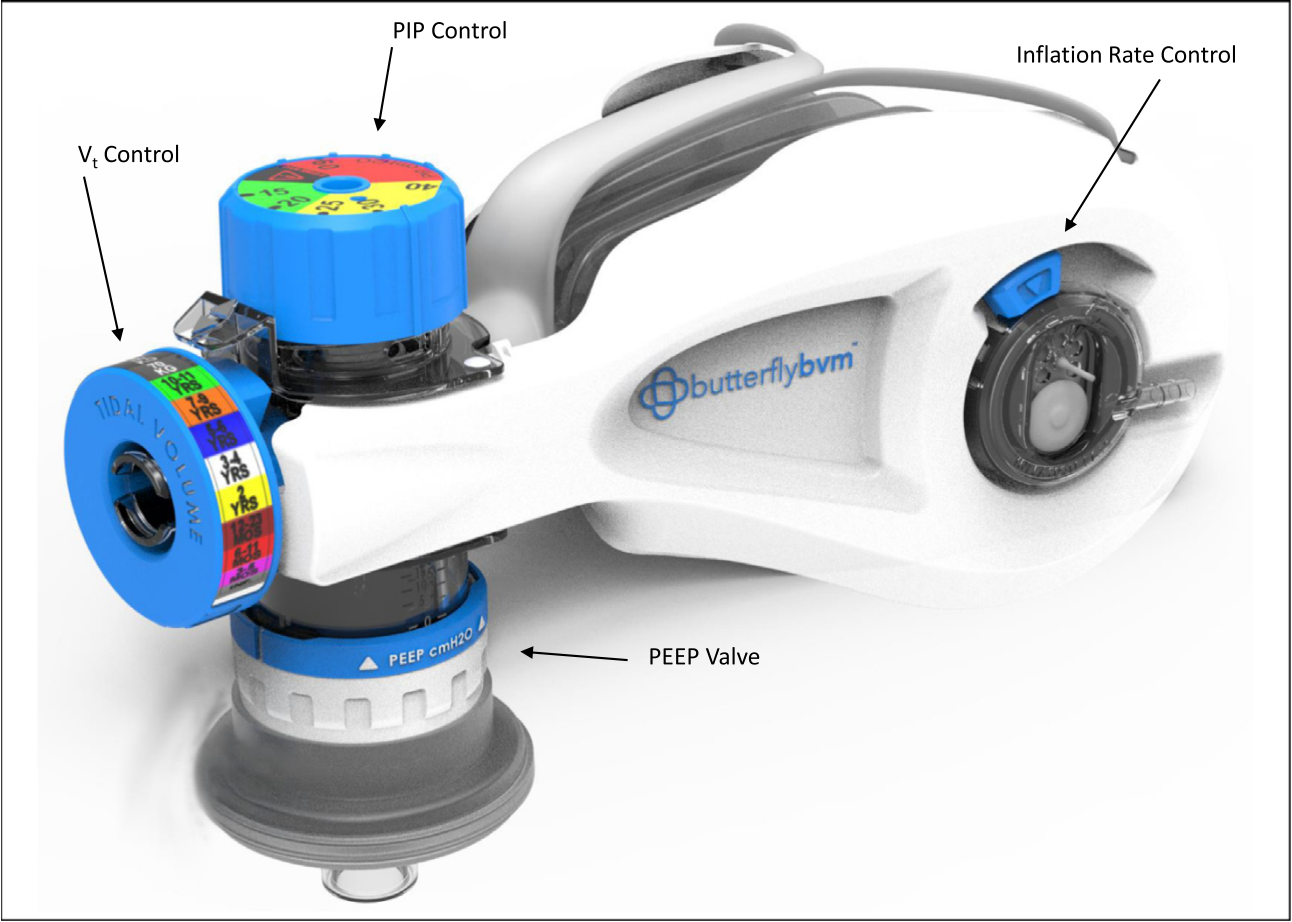
Render of the Butterfly BVM.

A prototypical device, known as the Butterfly BVM, has been developed to help address these concerns (Fig. 1). The Butterfly BVM intends to give users more control over V_t_, PIP, and rate than is typical with a traditional BVM and aims to be able to resuscitate full-term infants, children, teens, and adults with a single product.

The goal of this study was threefold. First, to assess the ability of airway managers to administer physiologically appropriate V_t_s, rates, and PIPs to adult and pediatric manikins while using a traditional BVM. Second, to compare these values against the V_t_s, rates, and PIPs administered with a novel device (the Butterfly BVM). Finally, we wished to assess the ability of users to rapidly learn how to adjust the settings of the Butterfly BVM.

## Methods

The study design (Supplement S1), protocol, and recruitment tools were approved by the Indiana University Institutional Review Board (IRB). Physicians (MDs or DOs) currently working in the field of Emergency Medicine (EM) with active Advanced Life Support (ALS) certification and a minimum of two years clinical experience since the time of first ALS certification were included. The study was conducted at The Simulation Center at Fairbanks Hall, with informed consent obtained prior to the study, and subjects were compensated with a gift card.

### Phase 1

Baseline resuscitation performance was first assessed using a traditional BVM (Ambu Spur II adult). Participants were asked if they had any questions regarding its operation. The device was then attached to a 7.5 mm cuffed endotracheal tube (ETT) of an intubated high-fidelity adult manikin (SimMan 3G, Laerdal) connected to a test lung (ASL 5000, IngMar, validated test lung, calibrated professionally) and set to default resistance/compliance settings. The manikin represented a 70 kg adult who had a pulse but was not breathing. Participants were asked to provide rescue breaths until told to stop while the research team observed from a separate room. After two calibrating breaths, V_t_s for the subsequent 20 breaths were documented along with the rate. Participants were offered a 30 second pause to rest their hands between this and all subsequent tests.

The above procedure was repeated with the Ambu Spur II Pediatric BVM. The device was attached to a 3.0 mm cuffed ETT of an intubated high-fidelity pediatric manikin (SimBaby, Laerdal) which was connected to a second test lung (ASL 5000, IngMar, validated test lung, calibrated professionally) with a resistance of 23cmH_2_O/L/s and a compliance of 15 ml/cmH_2_O (to approach ISO standards for pediatric test lungs as much as was possible).^18^ A digital pressure sensor (TruStability® SSCDANN005PGAA5, Honeywell, reported accuracy within 0.25 % of a measurement) was branched into the 15 mm adapter of the endotracheal tube. Participants were informed that the manikin represented a 2-year-old child who measured in the yellow Broselow zone who had a pulse but was not breathing. Twenty breaths were recorded in the same fashion as above.

A manometer was then added to the BVM. Participants were asked to provide rescue breaths for two additional tests, first while keeping PIP at or below 20cmH_2_O, and second while increasing the PIP between 25 and 30cmH_2_O.

### Phase 2

Participants were then given an introduction to the Butterfly BVM. They were told that the device was a bag-valve-mask, that it opened more slowly than a traditional BVM, and that it had been set to deliver the appropriate amount of air to a 70 kg adult. The device was compressed twice as a demonstration before being attached to the endotracheal tube of the adult manikin. Participants were not allowed to ask questions before using the Butterfly BVM. The same clinical scenario prompts and tests were administered as before. For the adult manikin test, the Butterfly BVM was set as follows: V_t_ patient selection – 70 kg, PEEP – 0 cmH_2_O, inflation speed – slow, PIP – infinite cmH_2_O. For the pediatric manikin tests, the device was set as follows: V_t_ patient selection – Yellow/2yr old, PEEP – 0 cmH_2_O, inflation speed – slow, PIP – 40cmH_2_O (first test) / 20cmH_2_O (second test) / 30cmH_2_O (final test).

### Phase 3

Participants then received a 2-minute introduction to the Butterfly BVM during which its features and settings adjustments were explained and demonstrated in detail. Participants were allowed up to 2 additional minutes to handle a Butterfly BVM device and ask questions before being administered a 14-question test of their ability to manipulate the various settings of the device to match a series of clinical scenarios. All participants completed an anonymous survey of their experience using the Butterfly BVM, elements liked or disliked, and their overall BVM preference. Each participant’s gender identity, hand size, and years of clinical experience (defined as years since first ALS certification) were documented.

### Statistical analysis

Pairwise t-tests compared the BVM devices; Utilizing the Linear Mixed Effect (LME) function of R, Analyses of Variance (ANOVA) tests were performed for BVM type, experience, hand size, and gender, while accounting for the random effect of participants on the data. Similar analysis was performed on the pediatric data for comparing the three different experimental conditions. Lastly, an assessment of normality was conducted by applying the Basic Test of Normality (e.g. Shapiro’s Test) to each of the participants. The detailed analyses are provided in the Supplemental Materials.

## Results

Nineteen participants (11 female, and 8 male) were included in the analysis of the adult manikin data. The mean experience since time of ALS certification was 5.1 years. Data from three additional participants were excluded from the pediatric V_t_ analysis due to technical errors in the data gathering. A final count of 16 participants (10 female, 6 male) were included in the pediatric analysis (mean years of experience 5.6).

**Fig. 2:**
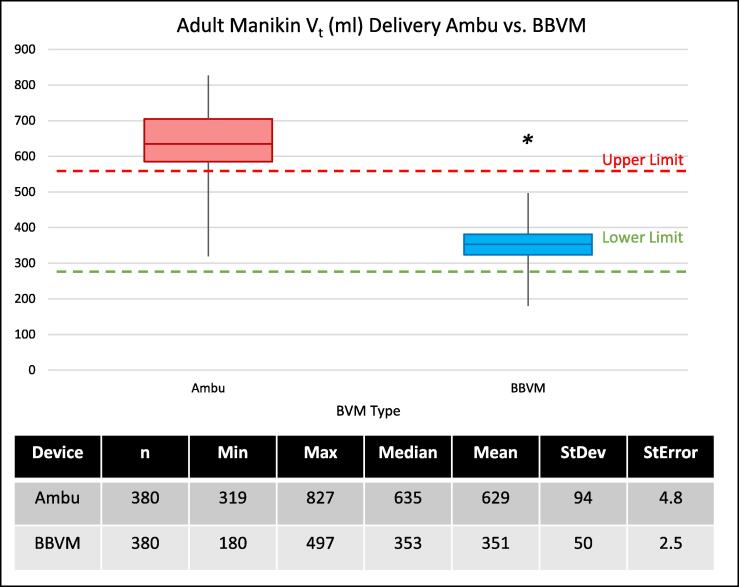
Tidal volumes delivered to an adult manikin by participants using a typical BVM, (Ambu Spur II adult) vs a Butterfly BVM. The green dashed line indicates the typical low, threshold Vt (4 ml/kg) for a patient of the stated size (70 kg), and the red dashed line represents, the typical max threshold Vt (8 ml/kg) for the same. *p < 0.01, 99 % CI (278 ± 8.69) ml.

The mean V_t_ delivered to the 70 kg adult manikin with the Ambu Spur II was 629 ml ± 94 ml vs 351 ml ± 50 ml delivered by the Butterfly BVM (Fig. 2). There was a significant difference in the mean measured volumes delivered between the two devices (p < 0.01, 99 % CI (278 ± 8.68 ml)). The Ambu Spur II also displayed greater variance, among individual participants and among all participants, than was found in the Butterfly BVM (Supplement S2). No significant variability over time and no systematic divergence was observed over repeated measures between the two devices over their trials (Supplement S3).

**Fig. 3:**
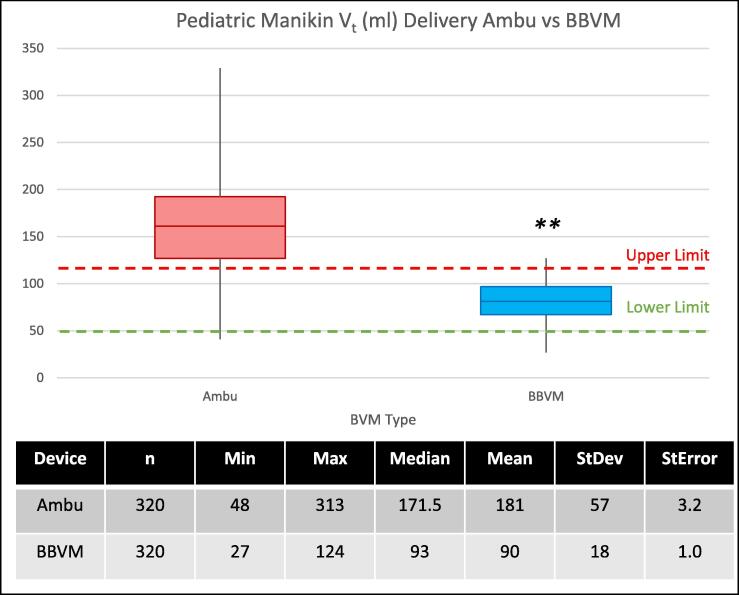
Tidal volumes delivered to a pediatric manikin by participants using a typical BVM (Ambu Spur II pediatric) vs the Butterfly BVM (set to the pediatric Vt setting). The green dashed line indicates the typical low threshold Vt (4 ml/kg) for a patient of the stated size (2 yr old child, 12–14 kg), and each red dashed line represents the typical max threshold Vt (8 ml/kg) for the same. **p < 0.01, 99 % CI (278 ± 8.69) ml.

The mean V_t_ delivered to the pediatric manikin with the Ambu Spur II was 181 ml ± 57 ml vs 90 ml ± 18 ml delivered by the Butterfly BVM (Fig. 3). There was a significant difference in the mean measured volumes delivered by the Ambu Spur II versus those delivered by the Butterfly BVM (p < 0.01, 99 % CI (91 ± 8.58 ml)).

Tidal volumes tended to be lower when participants were asked to keep pressures below the 20cmH_2_O target and tended to rise when participants were asked to increase pressures between 25 and 30cmH_2_O for both devices. Mean V_t_s were above range in all tests with the Ambu Spur II, and were within the acceptable range regardless of target PIP with the Butterfly BVM (p < 0.01) (Supplement S4).^19^

**Fig. 4:**
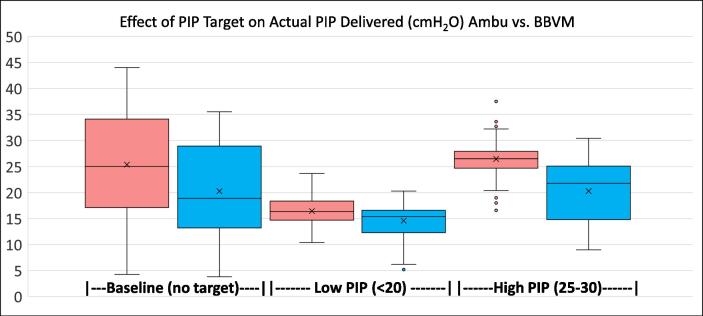
Peak inspiratory pressures between the three pediatric tests. There was more variability in pressures delivered during the baseline test with both devices (when participants were not given a target PIP). When given a target, peak pressures were more precise when lifesavers were using the Ambu Spur II with a manometer than when using the BBVM. The Ambu Spur II with manometer particularly outperformed the BBVM in the third test (participants were more successful at administering pressures within the target range of 25-30cmH2O).

PIP data from 1880 breaths was analyzed. Data from 40 breaths had been lost (20 Ambu and 20 BBVM) due to a technical failure. PIPs tended to be less precise when participants were given no target (Fig. 4), but participants using an Ambu Spur II Pediatric BVM with manometer tended to deliver more consistent PIPs over participants using the Butterfly BVM. Furthermore, the Butterfly BVM delivered most peak pressures below the target on the third test. However, there were 76 failures (breaths delivered with a PIP above the max) when participants were using the Ambu Spur II (Table 1). The highest pressure delivered by the Ambu Spur II was 7.9cmH_2_O above the target maximum. By contrast, the Butterfly BVM had two failures in 940 breaths, and the highest pressure delivered by the Butterfly BVM exceeded the target by 0.4cmH_2_O.

**Table 1:** Number of failures (out of 1880 recorded breaths) in which participants delivered a peak inspiratory pressure above the target. Of note, there were only two failures when participants used the manometer-free Butterfly BVM (each of which was less than 0.5cmH_2_O above target) in contrast to the higher failure rate, and higher failure values associated with use of the Ambu Spur II.

	**Baseline, BVM Set to Vent at 40cmH**_2_**O**		**Target Max PIP ≤20cmH**_2_**O**		**Target Max PIP ≤30cmH**_2_**O**	
	# of failures	Max PIP above goal (cmH_2_O)	# of failures	Max PIP above goal (cmH_2_O)	# of failures	Max PIP above goal (cmH_2_O)
Ambu Spur II	21	44	23	23.7	32	37.9
Butterfly BVM	0	N/A	1	20.3	1	30.4

The rate control mechanism of the Butterfly BVM prototype malfunctioned during the study (in that it allowed the device to inflate faster than intended), and inspiratory rates were similar between both the Ambu Spur II and the Butterfly BVM. However, despite this, minute ventilations were kept within range by the Butterfly BVM in 4 out of 4 tests but were above range in 2 out of 4 tests with the Ambu Spur II (Table 2).

**Table 2:** Average minute ventilation (MV) delivered by the Ambu Spur II vs the Butterfly BVM to adult and pediatric manikins (adult data on top). The Ambu Spur II delivered Mvs above range to the adult manikin and to the pediatric manikin in one of three tests. The Butterfly BVM delivered Mvs within the target ranges to both manikins in all tests. Of note, MV delivery with the Ambu Spur II Pediatric device reached unsafe levels when participants were asked to focus on raising PIP. *70 kg Adult target MV calculated as 70 kg × [4–8 ml/kg] × 10 breaths/min. **Yellow pediatric Broselow zone calculated as [12–14 kg] × [4–8 ml/kg] × [20–30 breaths/min].

**Adult**	**Avg V_t_ (ml)**	**Avg Rate (breaths/min)**	**Avg Minute Ventilation (L/min)**	Target Minute Ventilation Range* (L/min)
Ambu Spur II	635	11.6	**7.4**	2.8–5.6
Butterfly BVM	353	12.0	4.2	
**Pediatric**	**Avg V_t_ (ml)**	**Avg Rate(breaths/min)**	**Avg Minute Ventilation (L/min)**	Target Minute Ventilation Range** (L/min)
Ambu (baseline)	181	16.7	3.0	1.0–3.4
Ambu (low PIP)	136	18.5	2.5	
Ambu (high PIP)	173	21.1	**3.7**	
BBVM (baseline)	90	17.4	1.6	
BBVM (low PIP)	71	18.3	1.3	
BBVM (high PIP)	84	19.3	1.6	

An ANOVA, which account also for participant (random) effects, confirmed no significant difference in V_t_, PIPs, or rates delivered from physicians with different years of experience, genders, or hand sizes (see supplemental data).

Supplement S5 explains the core findings of phase 3. Out of a total of 266 tested adjustments, participants made the correct adjustment 258 times (∼97 % correct). When asked if they found the Butterfly BVM to be easy or difficult to use, participants responded with a mean score of 4.3 (using a Likert scale with 1 being “very difficult” and 5 being “very easy”). Likewise, respondents indicated that the adjustments on the Butterfly BVM were intuitive to perform with a mean score of 4.7 (using a Likert scale with 1 being “not intuitive” and 5 being “very intuitive”). Finally, when asked which BVM they would prefer to use, 74 % of participants indicated that they would prefer the Butterfly BVM to a traditional BVM.

## Discussion

Our results are consistent with earlier research showing that traditional BVMs are commonly misused by airway managers.^2^, ^9^, ^10^, ^11^ This was true during our relatively simple and highly controlled lifesaving scenarios. Our participants were also senior EM residents and fellows with active ALS certification and who had received repeated training on BVM use with a mean of 5.1 and 5.6 (adult and pediatric tests respectively) years of experience using BVMs. ALS certification includes training on the need to “avoid excessive ventilation” and physicians are now expected to maintain skills quarterly through the American Heart Association’s new Resuscitation Quality Improvement program. Furthermore, not one participant asked questions about the Ambu Spur II’s operation when given the opportunity suggesting a high degree of familiarity with these devices within our cohort.

One cause of hyperventilation is excessive V_t_. Target V_t_s are admittedly not well-defined for BVM users. Today, the AHA recommends giving sufficient air to produce chest rise or “approximately 500–600 ml” to adults.^6^ As BVMs become capable of controlling V_t_s with more precision, however, it may be possible to give users more defined targets. For our analysis we considered this general AHA recommendation and applied more specific targets given by the Agency for Healthcare Research and Quality (AHRQ). The AHRQ recommendations are intended for mechanical ventilators, but they reflect the current understanding of ideal V_t_s for critically ill patients. The AHRQ establishes 6–8 ml/kg as a target V_t_ for patients not in acute respiratory distress syndrome (ARDS), and 4–6 ml/kg for patients in ARDS.^19^ Assessing BVM V_t_ delivery based on targets defined by patient ideal body weight has been done by others in this field.^14^, ^15^

Though it did produce reliable chest rise in the adult manikin, the Butterfly BVM’s mean V_t_ delivery was below the AHA’s target V_t_ range. The Butterfly BVM was also below the target of 6 ml/kg for the simulated adult patient but within the AHRQ range of 4–8 ml/kg. This highlights room for improvement in the device’s design to increase the baseline V_t_ delivered to adults. The mean V_t_ delivered by the Ambu BVM to the manikin was near but just outside the specified AHRQ maximum, and it exceeded the AHA recommendation in both maximum and mean V_t_s delivered.

For children, assessment for chest rise remains essential, and the AHA admonishes users to avoid “excessive ventilation,” but it understandably does not give specific target V_t_ ranges for this diverse group of patients.^20^ Here the AHRQ guidelines may be of greater assistance. In our study, the Butterfly BVM delivered a mean volume that was essentially 6 ml/kg to the 2-year-old manikin while the Ambu Pediatric BVM delivered a mean volume of double this.

Hyperventilation is also caused by excessive rates of bagging. In this, the Butterfly BVM demonstrated no significant difference from the Ambu Spur II. This was most likely due to a failure of a strip of tape in the rate control mechanism. This tape was placed to partially occlude an aperture and restrict airflow into the device, but it failed to properly bind to the prototype. The final product will be made without the use of this tape. Thus, while other failures are possible, this specific failure will not occur in the final device.

Our study confirmed previous research showing that airway managers are “task saturated” when using a BVM.^10^, ^11^, ^12^, ^13^ This was readily seen when participants administered air with a larger minute ventilation when they were asked to increase the PIP given to the pediatric manikin while using the Ambu Spur II (Table 2). The Butterfly BVM enables users to set parameters such as V_t_ and PIP before use and thus reduces the amount of active monitoring of the device required while performing a resuscitation. Increased focus can then be given to other clinical inputs such as monitoring chest rise/fall or tracking end-tidal CO_2_. Indeed, in the post study survey, the ability of the device to assist users in regulating these ventilation parameters was one of the most frequently expressed reasons why users preferred the Butterfly BVM over a traditional BVM.

In addition, the Butterfly BVM’s variable PIP mechanism without manometer demonstrated superiority to the Ambu Spur II’s popoff with manometer in keeping peak inspiratory pressures from exceeding targets. However, it is likely, that at least some of this benefit was artificial. 3D printed materials, like those used in this Butterfly BVM prototype, are more prone to air leak than commercial quality units. This leak may have result in lower delivered peak pressures than would have been expected with a commercial-ready device.

Finally, the 30-second introduction participants received when first shown the Butterfly BVM demonstrated that the device could be used properly by an airway manager who did not already know the device assuming the settings were first adjusted by a user familiar with the device. The final phase of testing confirmed that the Butterfly BVM can readily be adjusted to the appropriate setting, at least during an idealized test, with a high degree of success after only a 2–4 minute introduction.

We note that the Butterfly BVM is not the only manual ventilator device which aims to address the problems of excessive rates and V_t_s. Devices such as the Small Adult CPR-2 by Mercury Medical, the Smart Bag by O_Two Medical, and the “V_t_ Select” by Pulmodyne are similarly attempting to address this issue. The trend of the industry toward devices that address critical safety gaps is encouraging.

### Limitations

There were several technical errors in the study. These include those mentioned above, and there was a technical error in the high-fidelity pediatric manikin that caused it to sometimes give an audible cough and to not always reliably show chest rise/fall during the study. Participants were made aware of these concerns before the pediatric tests. The missing chest rise was intermittent and present at times in both the Ambu and Butterfly BVM tests, but this glitch may have been responsible for some of the elevated V_t_s given to the pediatric manikin by participants using the Ambu Spur II.

Next, this simulation study was not designed to be able to capture the truest view of an airway manager’s behavior. Participants were alone in a room with the manikin, and they were asked to focus only on delivering appropriate rescue breathing in the absence of other clinical care. In studies of traditional BVMs used in more complex code scenarios, respiratory rates observed have generally been much higher than were observed in our study.^9^, ^10^, ^11^ Differences in respiratory rates and V_t_s between the Butterfly BVM and a traditional BVM may be more pronounced in higher-stress scenarios in the clinical setting.

This study also only involved EM physicians. While this is an important group of BVM users, there are others who may be even more likely in a code scenario to handle a BVM. Future work may help to test the Butterfly BVM within multidisciplinary teams that better reflect the diversity of BVM users.

## Conclusions

This study confirmed the tendency of airway managers to give excessive volumes of air with a traditional BVM to simulated patients. Though volumes and pressures delivered by the Butterfly BVM were at times lower than desired, the device demonstrated superiority to the Ambu Spur II in delivering reduced volumes and pressures to both adult and pediatric manikins without the need for a manometer or external V_t_ assisting device. Additional work is needed to evaluate the performance of the Butterfly BVM with other healthcare professionals, and to determine if these advancements will translate to improved outcomes for patients.

### Support

This material is based upon work supported in its entirety by the National Science Foundation (grant # 2124771) and the Southwest Pediatric Device Consortium and American Academy of Pediatrics Section on Advances in Therapeutics and Technology (grant # P50FD006428). Any findings, opinions, conclusions, or recommendations expressed in this material are those of the authors and do not necessarily reflect the views of the NSF, the SWPDC, or the AAP SOATT.

## Declarations of interest

The study design, protocol, recruitment tools, and data collection mechanisms, were all approved by the Indiana University Institutional Review Board. JGM and ACS have a financial interest in Compact Medical and could benefit from the results of this research. JGM and ACS were involved in the study design, data gathering, and drafting of results and conclusions. Neither JGM nor ACS were directly involved in the recruitment of participants, in obtaining informed consent, or in analyzing the data. DDC was responsible for recruitment and consent of study participants, while AB was directly responsible for statistical analysis and was blinded to participants. This research has not been published elsewhere. It was funded entirely by the financial grant support mentioned above and not by Compact Medical. JGM’s and ACS’s conflicts of interest were reviewed by Indiana University and were appropriately managed to maintain objectivity.

## CRediT authorship contribution statement

**Jonathan G. Merrell:** Conceptualization, Methodology, Investigation, Resources, Writing – original draft, Writing – review & editing, Visualization, Project administration, Funding acquisition. **Adam C. Scott:** Conceptualization, Methodology, Investigation, Resources, Data curation, Writing – original draft, Writing – review & editing, Visualization, Project administration, Funding acquisition. **Ryan Stambro:** Investigation, Resources, Writing – review & editing. **Amit Boukai:** Formal analysis, Data curation, Writing – original draft, Writing – review & editing. **Dylan D. Cooper:** Validation, Investigation, Resources, Writing – original draft, Writing – review & editing, Supervision.

## Appendix A Supplementary material

Supplementary data 1 Adult Mannequin Trial, Pediatric Mannequin Trial, PIP under the Baseline Condition, Vt under the Baseline Condition, PIP under the Low PIP Condition, Vt under the Low PIP Condition, PIP under the High PIP Condition, and Vt under the High PIP Condition.

## References

[1] Berg M.D., Idris A.H., Berg R.A. (1998). Severe ventilatory compromise due to gastric distention during pediatric cardiopulmonary resuscitation. Resuscitation.

[2] Aufderheide T.P., Sigurdsson G., Pirrallo R.G. (2004). Hyperventilation-induced hypotension during cardiopulmonary resuscitation. Circulation.

[3] Ioannidis G., Lazaridis G., Baka S. (2015). Barotrauma and pneumothorax. J Thorac Dis.

[4] Acute Respiratory Distress Syndrome N, Brower R.G., Matthay M.A. (2000). Ventilation with lower tidal volumes as compared with traditional tidal volumes for acute lung injury and the acute respiratory distress syndrome. N Engl J Med.

[5] Kleinman M.E., Brennan E.E., Goldberger Z.D. (2015). Part 5: Adult Basic Life Support and Cardiopulmonary Resuscitation Quality: 2015 American Heart Association Guidelines Update for Cardiopulmonary Resuscitation and Emergency Cardiovascular Care. Circulation.

[6] Panchal A.R., Bartos J.A., Cabanas J.G. (2020). Part 3: Adult Basic and Advanced Life Support: 2020 American Heart Association Guidelines for Cardiopulmonary Resuscitation and Emergency Cardiovascular Care. Circulation.

[7] Field J.M., Hazinski M.F., Sayre M.R. (2010). Part 1: executive summary: 2010 American Heart Association Guidelines for Cardiopulmonary Resuscitation and Emergency Cardiovascular Care. Circulation.

[8] Perkins G.D., Handley A.J., Koster R.W. (2015). European Resuscitation Council Guidelines for Resuscitation 2015: Section 2. Adult basic life support and automated external defibrillation. Resuscitation.

[9] Aufderheide T.P., Lurie K.G. (2004). Death by hyperventilation: a common and life-threatening problem during cardiopulmonary resuscitation. Crit Care Med.

[10] Losert H., Sterz F., Kohler K. (2006). Quality of cardiopulmonary resuscitation among highly trained staff in an emergency department setting. Arch Intern Med.

[11] Niebauer J.M., White M.L., Zinkan J.L., Youngblood A.Q., Tofil N.M. (2011). Hyperventilation in pediatric resuscitation: performance in simulated pediatric medical emergencies. Pediatrics.

[12] Ambu Inc. Ambu Spur II Datasheet. LBL-008649 - V01 - 7/2022. https://www.ambuusa.com/emergency-care-and-training/resuscitators/product/ambu-spur-ii.

[13] Dafilou B., Schwester D., Ruhl N., Marques-Baptista A. (2020). It's In The Bag: Tidal Volumes in Adult and Pediatric Bag Valve Masks. West J Emerg Med.

[14] Siegler J., Kroll M., Wojcik S., Moy H.P. (2017). Can EMS Providers Provide Appropriate Tidal Volumes in a Simulated Adult-sized Patient with a Pediatric-sized Bag-Valve-Mask?. Prehosp Emerg Care.

[15] Kroll M., Das J., Siegler J. (2019). Can Altering Grip Technique and Bag Size Optimize Volume Delivered with Bag-Valve-Mask by Emergency Medical Service Providers?. Prehosp Emerg Care.

[16] Kern K.B., Stickney R.E., Gallison L., Smith R.E. (2010). Metronome improves compression and ventilation rates during CPR on a manikin in a randomized trial. Resuscitation.

[17] Milander M.M., Hiscok P.S., Sanders A.B., Kern K.B., Berg R.A., Ewy G.A. (1995). Chest compression and ventilation rates during cardiopulmonary resuscitation: the effects of audible tone guidance. Acad Emerg Med.

[18] International Organization for Standardization. Lung Ventilators - Part 4: Particular requirements for operator-powered resuscitators; 2002.

[19] Agency for Healthcare Research and Quality. Low Tidal Volume Ventilation Facts. January 2017. https://www.ahrq.gov/hai/tools/mvp/modules/technical/ltvv-fact-sheet.html.

[20] Topjian A.A., Raymond T.T., Atkins D. (2020). Part 4: Pediatric Basic and Advanced Life Support: 2020 American Heart Association Guidelines for Cardiopulmonary Resuscitation and Emergency Cardiovascular Care. Circulation.

