# Supplementary material for: Improved simulated ventilation with a novel tidal volume and peak inspiratory pressure controlling bag valve mask: A pilot study
Source: Resusc Plus. 2023 Jan 5;13:100350. doi: 10.1016/j.resplu.2022.100350 (PMC9841173; doi:10.1016/j.resplu.2022.100350)
Supplement: Supplementary data 1 — Adult Mannequin Trial, Pediatric Mannequin Trial, PIP under the Baseline Condition, Vt under the Baseline Condition, PIP under the Low PIP Condition, Vt under the Low PIP Condition, PIP under the High PIP Condition, and Vt under the High PIP Condition. [file mmc1.pdf]

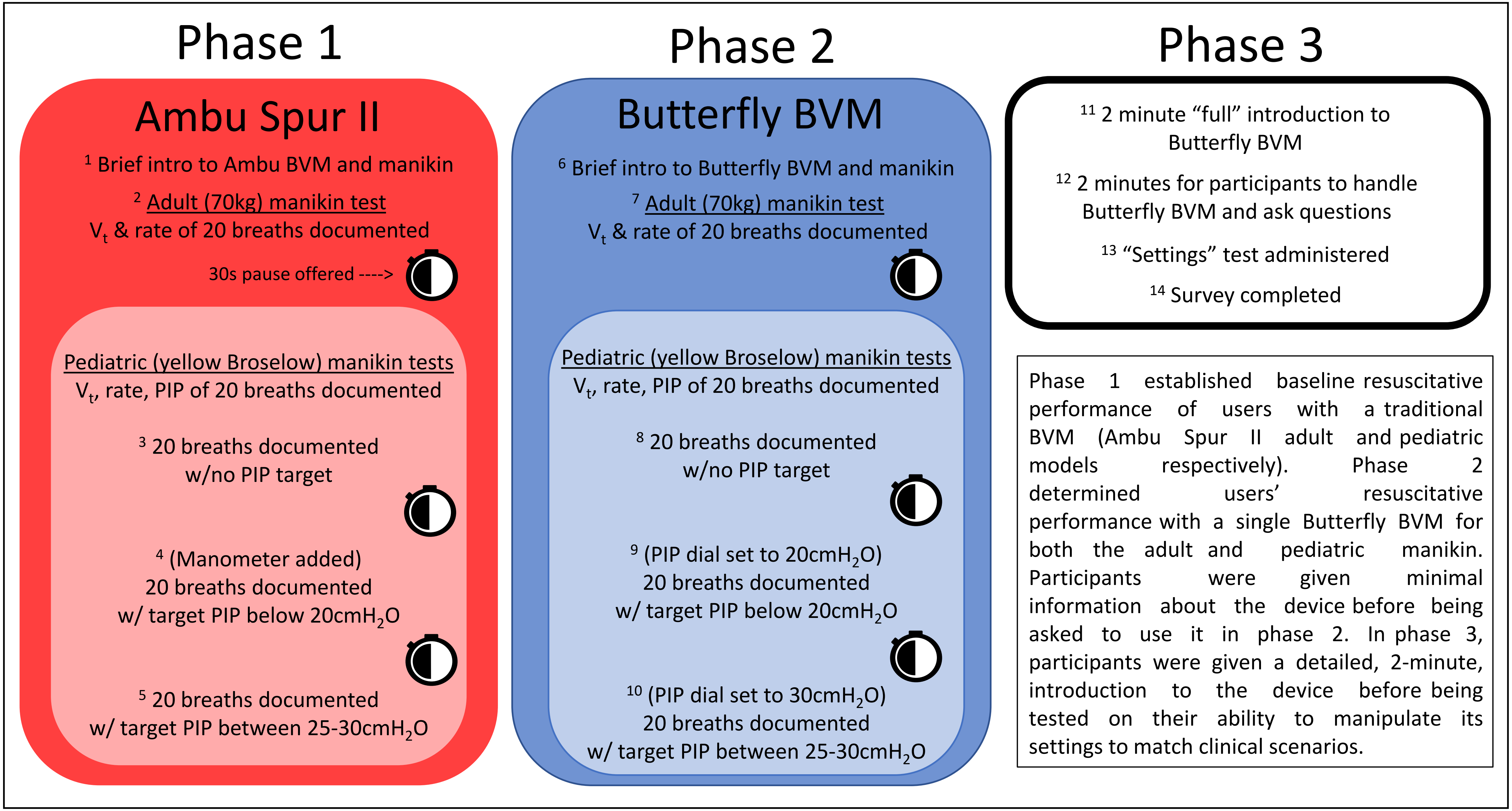

Figure S1: flowchart outlining study methods (steps 1-14).

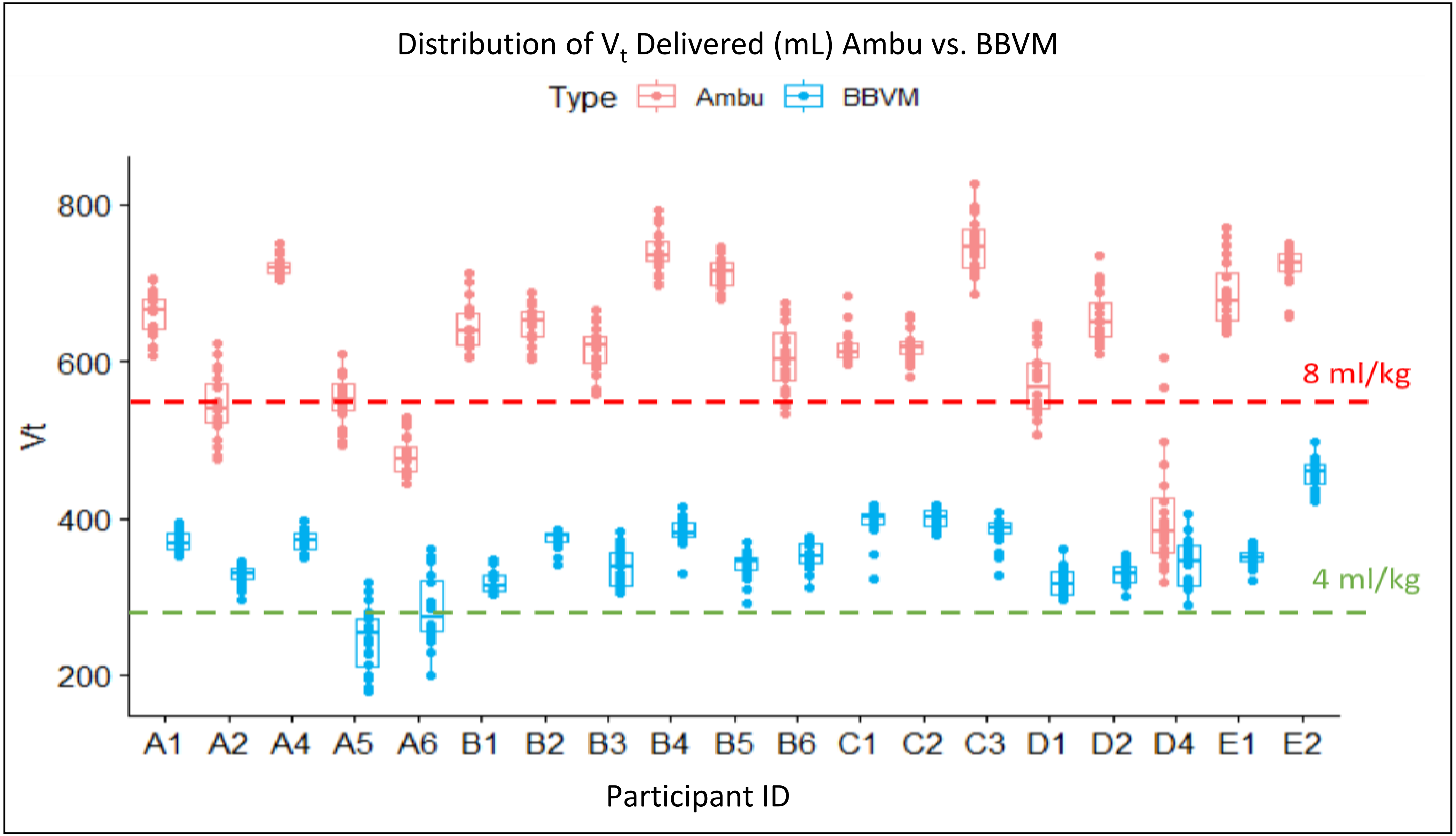

Figure S2: Visualizing the distribution of V_t among users by type of BVM. Note that there is typically reduced V_t variability within a single user and across multiple users of the Butterfly BVM vs with those using a traditional BVM.

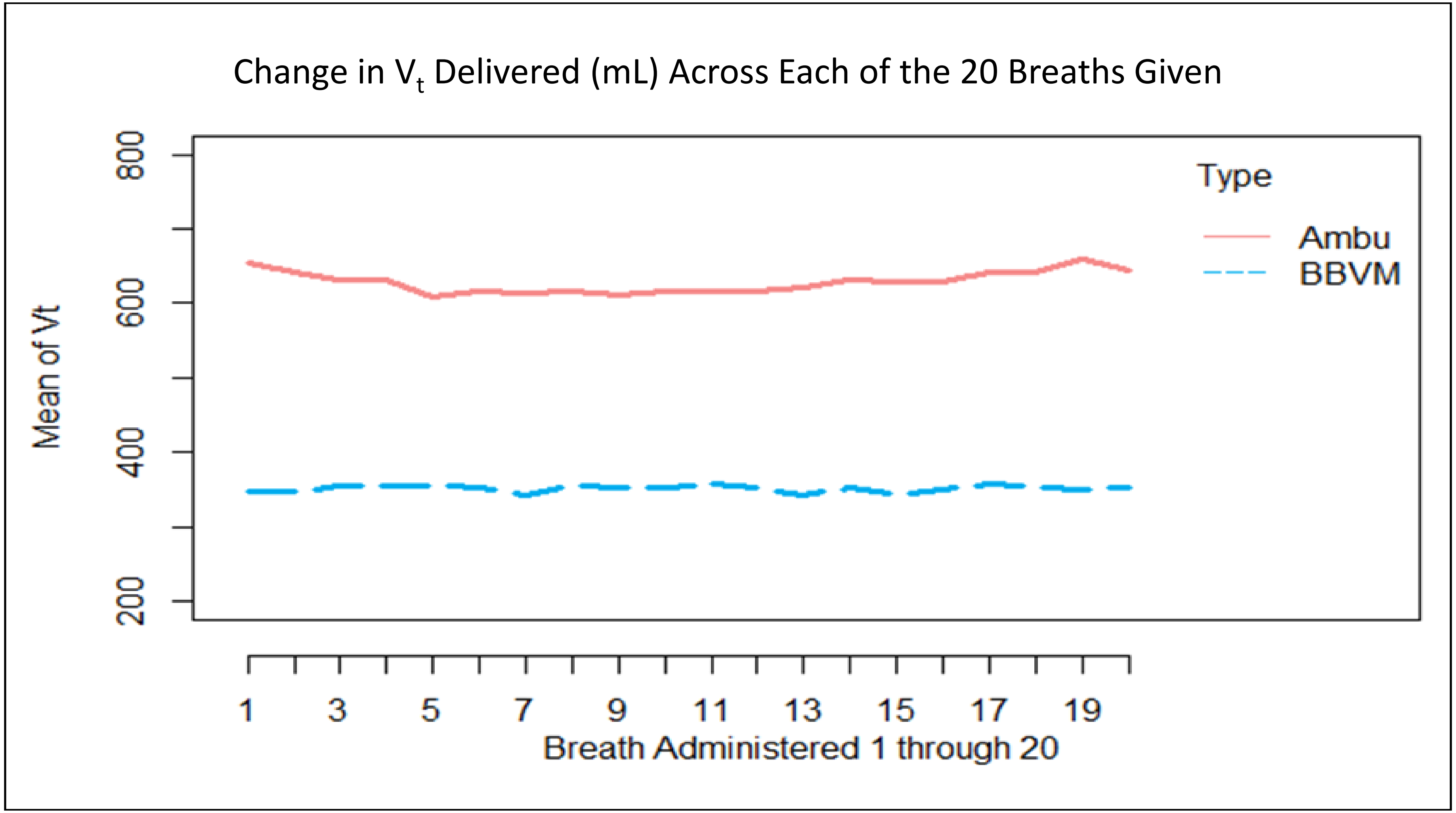

Figure S3: The "interaction" between devices' repeated measurements across all trials and across all participants reveals no significant variability over time, nor any systematic divergence between the two devices over their trials.

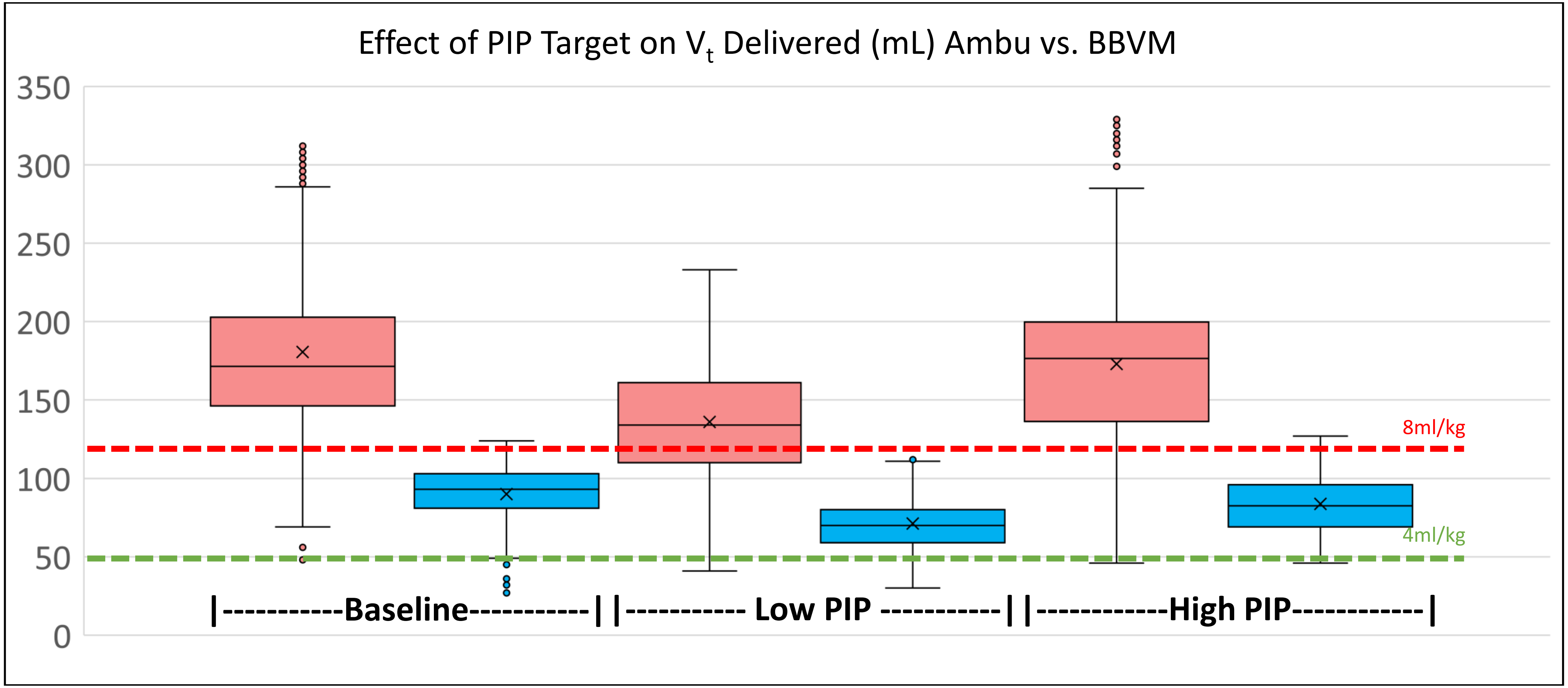

Figure S4: Changes in V_t when participants are asked to deliver air without regard to PIP (baseline) vs. air at a low PIP (<20cmH_2O) and air at a high PIP (between 25 and 30cmH_2O). The green dashed line indicates the typical low threshold V_t (4ml/kg) for a patient of the stated size (2yr old child, 12-14kg), and the red dashed line represents the typical max threshold V_t (8ml/kg) for the same. The Butterfly BVM was more accurate and more precise at delivering tidal volumes within the physiologic range than the Ambu Spur II irrespective of the stated peak pressure target. $p<0.01$

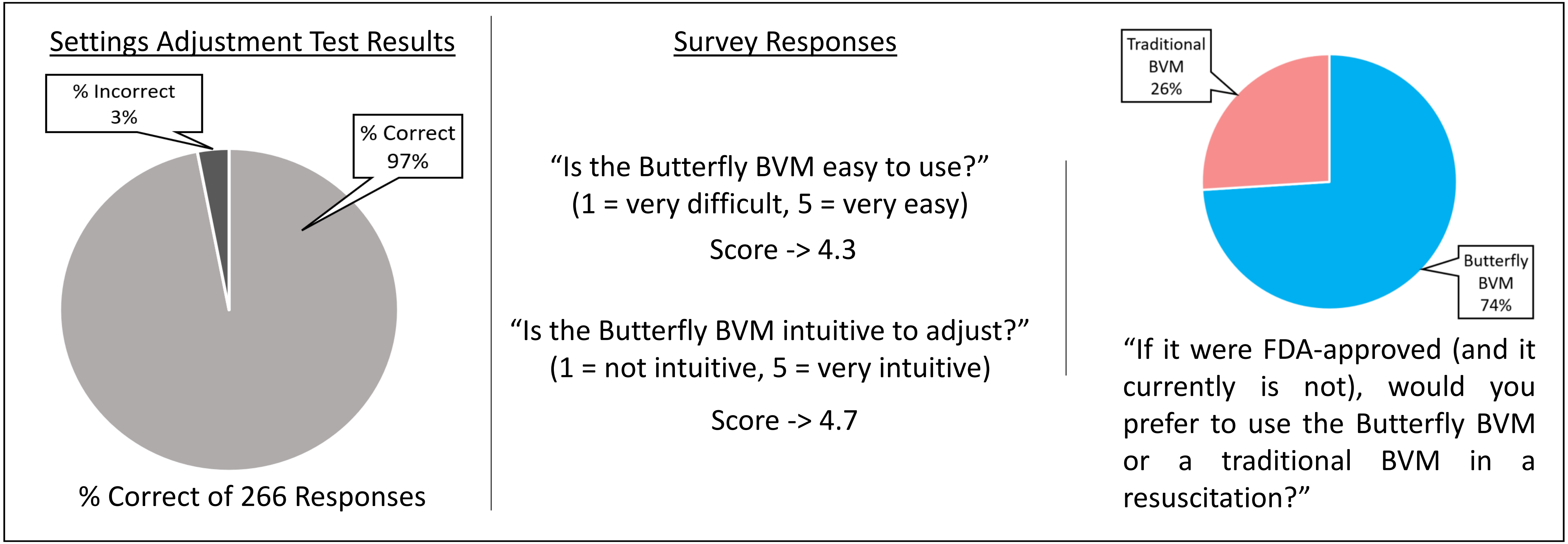

Figure S5: Results of settings adjustment test (left) and selection of survey responses (right).
