# Supplementary material for: Improved simulated ventilation with a novel tidal volume and peak inspiratory pressure controlling bag valve mask: A pilot study
Source: Resusc Plus. 2023 Jan 5;13:100350. doi: 10.1016/j.resplu.2022.100350 (PMC9841173; doi:10.1016/j.resplu.2022.100350)
Supplement: Supplementary data 2 [file mmc2.pdf]

Improved Ventilation with a Novel Tidal Volume and Peak Inspiratory Pressure Controlling Bag Valve Mask–A Pilot Study*

Supplemental Report #1

Jonathan Merrell, Adam Scott, Ryan Stambro, Amit Boukai and Dylan Cooper

Statistical Analysis of the Adult Mannequin Trial

• The raw Data

Nineteen participants (all EM physicians (either MD or Do) with active ACLS certification and a minimum one year of clinical experience) were recruited for the study. Each participant was classified according to their years of experience (T1= "Less than 5 years", T2= "Between 5 to 10 years", T3= "Over 10 years") and according to their Gender (8 males and 11 females). Under a strict experimental protocol, the participant was asked to provide rescue ventilation to a simulated adult patient (as a high-fidelity intubated adult mannequin connected to an ASL 5000 machine). Each participant delivered 20 successive rescue breaths (trails) using a traditional BVM resuscitator ("Ambu") and then, after a brief pause, delivered 20 additional rescue breaths using a novel Butterfly BVM resuscitator ("BBVM"), and the tidal volume (V_t) Measurements, among others (Rate and MV), were recorded after each breath.

• The statistical procedures:

Below we provide descriptive statistics of the measured variable (V_t), by the two types of resuscitators per each of the participants' groups (by Gender and by Years' Experience). As this was carried out as a Pilot Study, these groups were unbalanced. The V_t means under the two resuscitator types were compared by each group and by participants, accounting also for the participants' (random) effect utilizing standard paired t-test as well as the the AOV (Analysis Of Variance) and the LME (Linear Mixed Effect) functions. Throughout, the significance level used for all testing as well as for Confidence Intervals was $\alpha = 0.01$.

• Setting up the Data Frame (Adult Mannequin Experiment)

```
## 'data.frame':    380 obs. of  7 variables:
##  $ ID    : Factor w/ 19 levels "A1","A2","A4",..: 1 1 1 1 1 1 1 1 1 1 ...
##  $ Gender: Factor w/ 2 levels "F","M": 2 2 2 2 2 2 2 2 2 2 ...
##  $ Exp   : Factor w/ 3 levels "T1","T2","T3": 1 1 1 1 1 1 1 1 1 1 ...
##  $ Trial : int  1 2 3 4 5 6 7 8 9 10 ...
##  $ Ambu  : int  634 644 666 687 665 668 705 664 643 618 ...
##  $ BBVM  : int  395 395 391 384 381 366 374 380 370 361 ...
##  $ Size  : num  19.5 19.5 19.5 19.5 19.5 19.5 19.5 19.5 19.5 19.5 ...
```

*Supplemental Report to the *Improved Ventilation with a Novel Tidal Volume and Peak Inspiratory Pressure Controlling Bag Valve Mask–A Pilot Study*

- **The Structure of the Adult Data**

ID	Gender	Exp	Trial	Ambu	BBVM	Size
A1	M	T1	1	634	395	19.5
A1	M	T1	2	644	395	19.5
A1	M	T1	3	666	391	19.5
A1	M	T1	4	687	384	19.5
A1	M	T1	5	665	381	19.5

- **Changing the data frame from a wide format to a Long Style**

```
## 'data.frame':    760 obs. of  7 variables:
##  $ ID    : Factor w/ 19 levels "A1","A2","A4",..: 1 1 1 1 1 1 1 1 1 1 ...
##  $ Gender: Factor w/ 2 levels "F","M": 2 2 2 2 2 2 2 2 2 2 ...
##  $ Exp   : Factor w/ 3 levels "T1","T2","T3": 1 1 1 1 1 1 1 1 1 1 ...
##  $ Trial : int  1 2 3 4 5 6 7 8 9 10 ...
##  $ Size  : num  19.5 19.5 19.5 19.5 19.5 19.5 19.5 19.5 19.5 19.5 ...
##  $ Type  : Factor w/ 2 levels "Ambu","BBVM": 1 1 1 1 1 1 1 1 1 1 ...
##  $ Vt    : int  634 644 666 687 665 668 705 664 643 618 ...
```

- **The numberof participants per each Gender by Experience group**

	F	M
T1	7	6
T2	4	0
T3	0	2

- **The number of participants per each Gender by Hand Size group**

	F	M
15.5	1	0
16.5	1	0
17.5	4	0
18	5	0
18.5	0	1
19	0	4
19.5	0	3

- **The sample sizes per each Gender by Experience group**

Exp	Gender	n	prop
T1	F	280	53.8
T1	M	240	46.2
T2	F	160	100.0

Exp	Gender	n	prop
T3	M	80	100.0

- **Summary statistics for Vt measurements by the two BVM types (while ignoring all other factors)**

Type	variable	n	min	max	median	iqr	mean	sd	se	ci
Ambu	Vt	380	319	827	635	119.50	628.679	93.657	4.805	9.447
BBVM	Vt	380	180	497	353	57.25	351.468	49.589	2.544	5.002

- **Visualizing the Distrubution of Vt by the two BVM Types (while ignoring all other factors)**

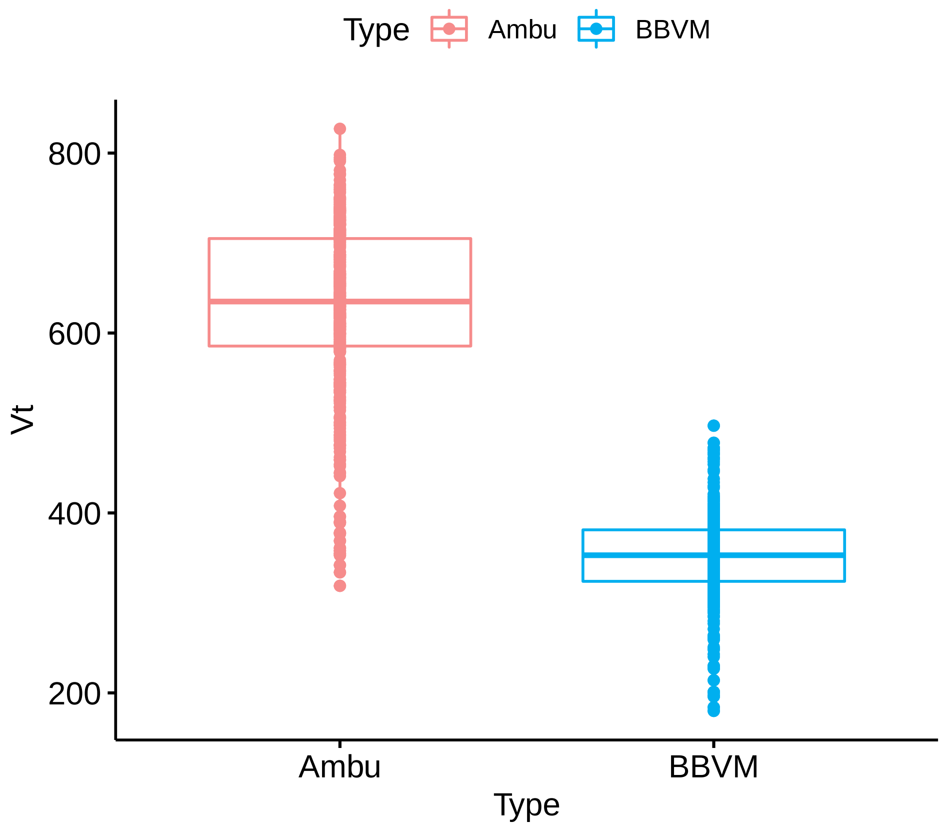

- **Summary statistics for Vt by Gender and Type**

Gender	Type	variable	n	min	max	median	iqr	mean	sd	se	ci
F	Ambu	Vt	220	319	827	617	114.50	596.073	99.824	6.730	13.264
M	Ambu	Vt	160	506	793	685	94.50	673.513	60.743	4.802	9.484
F	BBVM	Vt	220	180	418	339	52.00	333.541	47.455	3.199	6.306

Gender	Type	variable	n	min	max	median	iqr	mean	sd	se	ci
M	BBVM	Vt	160	292	497	375	43.25	376.119	41.275	3.263	6.445

- **Visualizing the Distrubution of Vt by Type for each Gender**

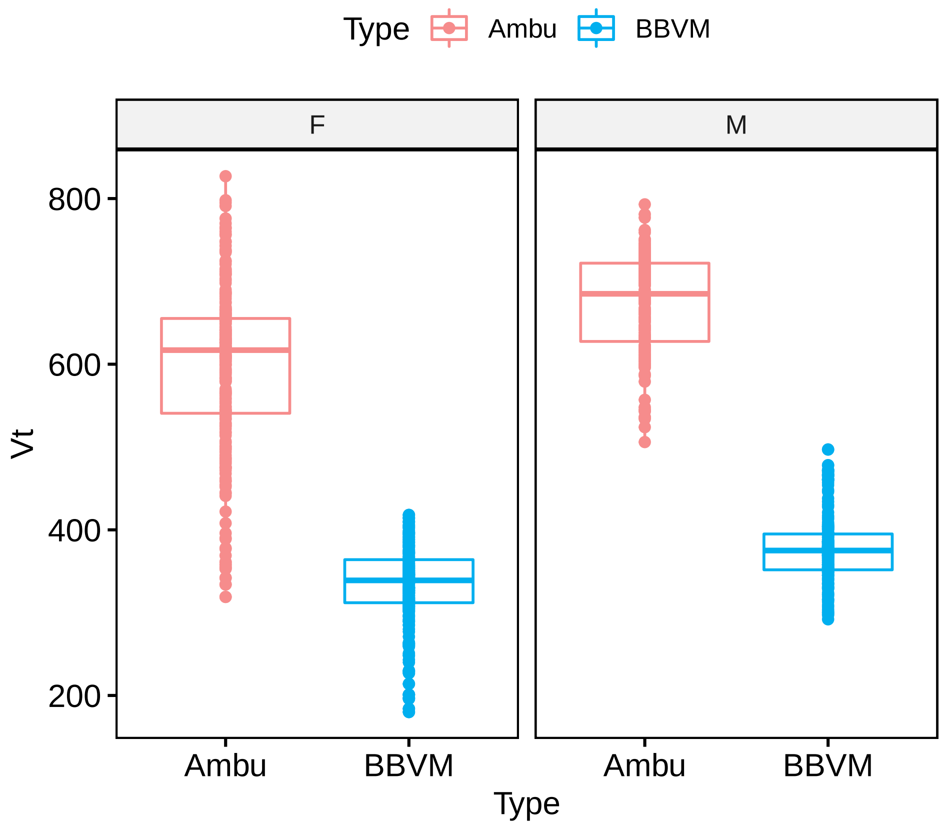

- **Summary statistics for Vt by Type and Years of Expereince**

Exp	Type	variable	n	min	max	median	iqr	mean	sd	se	ci
T1	Ambu	Vt	260	319	793	621.0	140.50	607.342	99.297	6.158	12.126
T2	Ambu	Vt	80	581	827	661.0	93.00	677.400	58.158	6.502	12.942
T3	Ambu	Vt	40	596	750	659.5	113.00	669.925	57.113	9.030	18.266
T1	BBVM	Vt	260	180	415	344.5	56.25	335.842	44.117	2.736	5.388
T2	BBVM	Vt	80	301	418	360.0	52.75	365.663	31.267	3.496	6.958
T3	BBVM	Vt	40	324	497	419.0	59.25	424.650	38.182	6.037	12.211

- **Visualizing the Distrubution of Vt by Type and Years of Expereince**

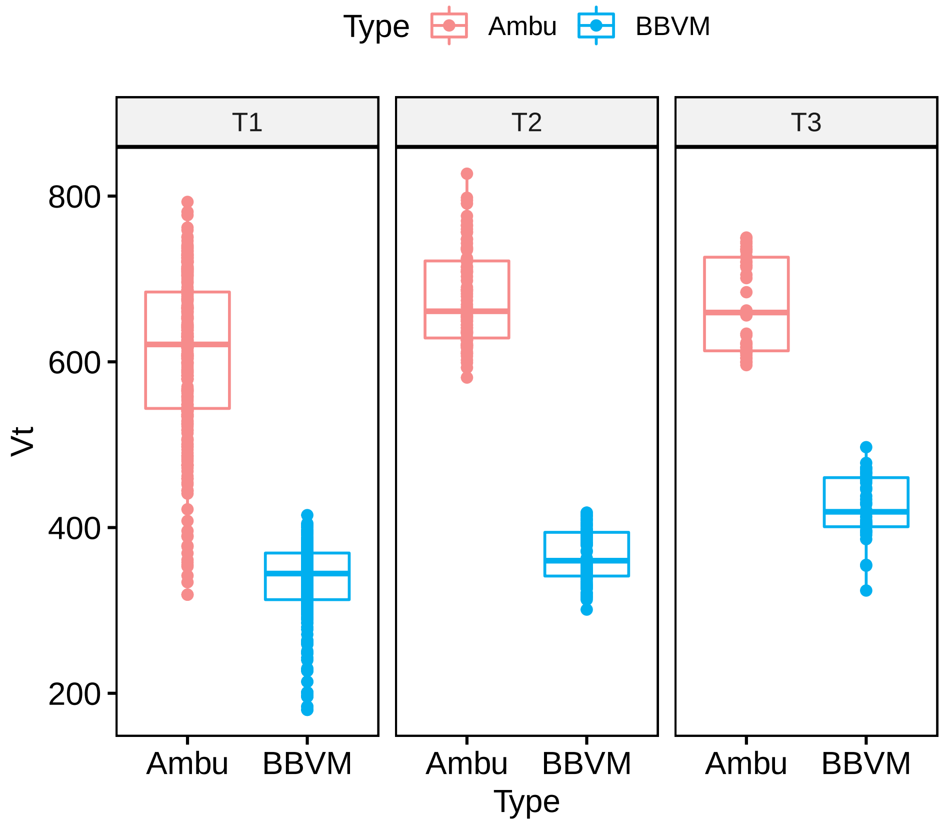

- **Summary statistics of Vt by Participants and Type**

ID	Type	variable	n	min	max	median	iqr	mean	sd	se	ci
A1	Ambu	Vt	20	607	705	664.5	37.75	658.75	28.639	6.404	13.404
A2	Ambu	Vt	20	475	624	541.0	49.00	544.40	41.649	9.313	19.493
A4	Ambu	Vt	20	704	750	720.0	14.25	720.25	11.933	2.668	5.585
A5	Ambu	Vt	20	494	610	550.5	33.25	551.85	33.274	7.440	15.573
A6	Ambu	Vt	20	444	528	475.5	31.50	479.25	25.041	5.599	11.719
B1	Ambu	Vt	20	604	712	638.0	41.25	643.45	30.741	6.874	14.387
B2	Ambu	Vt	20	603	687	652.5	29.75	646.85	24.603	5.501	11.514
B3	Ambu	Vt	20	559	666	620.5	33.50	617.00	28.466	6.365	13.323
B4	Ambu	Vt	20	697	793	734.0	25.75	738.20	26.100	5.836	12.215
B5	Ambu	Vt	20	679	746	714.5	29.25	711.15	19.658	4.396	9.200
B6	Ambu	Vt	20	534	675	603.5	60.50	604.85	44.544	9.960	20.847
C1	Ambu	Vt	20	596	684	612.5	17.50	618.35	21.149	4.729	9.898
C2	Ambu	Vt	20	581	658	619.5	16.75	618.80	18.716	4.185	8.759
C3	Ambu	Vt	20	685	827	745.5	48.25	748.55	36.306	8.118	16.992
D1	Ambu	Vt	20	506	647	568.0	57.00	573.05	41.503	9.280	19.424
D2	Ambu	Vt	20	610	735	650.0	40.75	657.20	33.779	7.553	15.809
D4	Ambu	Vt	20	319	604	383.5	69.50	406.40	75.680	16.922	35.419
E1	Ambu	Vt	20	636	770	676.5	61.00	685.05	42.653	9.538	19.962

ID	Type	variable	n	min	max	median	iqr	mean	sd	se	ci
E2	Ambu	Vt	20	656	750	726.5	21.25	721.50	25.461	5.693	11.916
A1	BBVM	Vt	20	352	395	368.5	19.25	371.25	13.042	2.916	6.104
A2	BBVM	Vt	20	297	346	330.0	14.00	327.65	13.504	3.019	6.320
A4	BBVM	Vt	20	349	397	371.5	21.25	371.35	14.087	3.150	6.593
A5	BBVM	Vt	20	180	318	254.0	61.75	245.75	41.107	9.192	19.239
A6	BBVM	Vt	20	201	362	274.5	63.25	284.35	44.793	10.016	20.964
B1	BBVM	Vt	20	302	348	314.5	20.75	317.35	13.781	3.082	6.450
B2	BBVM	Vt	20	342	386	378.0	12.50	374.10	11.698	2.616	5.475
B3	BBVM	Vt	20	305	384	339.5	43.25	338.35	24.748	5.534	11.582
B4	BBVM	Vt	20	329	415	381.5	18.75	383.15	18.039	4.034	8.442
B5	BBVM	Vt	20	292	369	345.0	15.50	341.15	17.842	3.990	8.350
B6	BBVM	Vt	20	312	376	352.0	26.50	352.75	17.571	3.929	8.223
C1	BBVM	Vt	20	324	417	401.0	12.00	393.05	22.956	5.133	10.744
C2	BBVM	Vt	20	379	418	402.5	20.75	400.35	13.144	2.939	6.152
C3	BBVM	Vt	20	328	409	388.5	14.50	383.10	18.851	4.215	8.823
D1	BBVM	Vt	20	297	360	316.0	27.50	318.65	16.968	3.794	7.941
D2	BBVM	Vt	20	301	355	330.0	20.00	329.40	13.496	3.018	6.316
D4	BBVM	Vt	20	289	405	345.0	50.25	340.10	33.586	7.510	15.719
E1	BBVM	Vt	20	321	371	349.5	11.00	349.80	10.909	2.439	5.106
E2	BBVM	Vt	20	421	497	460.5	23.75	456.25	19.059	4.262	8.920

- **Visualizing the Distrubution of Vt by Participants and Type**

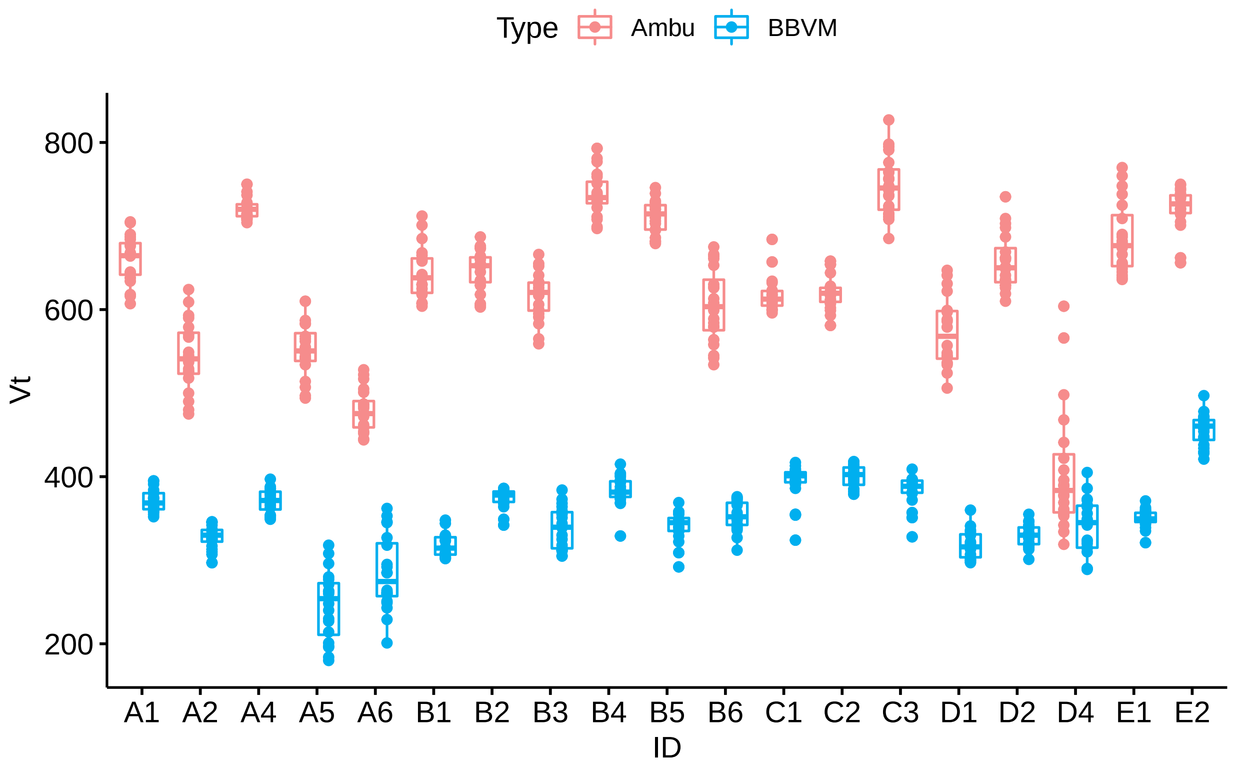

- **Basic test of Normality (Shapiro's Test applied to each Paricipant by Type)**

ID	Type	variable	statistic	p
A1	Ambu	Vt	0.9636705	0.6194770
A2	Ambu	Vt	0.9761551	0.8754798
A4	Ambu	Vt	0.9227670	0.1120222
A5	Ambu	Vt	0.9652013	0.6520522
A6	Ambu	Vt	0.9467246	0.3200900
B1	Ambu	Vt	0.9272236	0.1365261
B2	Ambu	Vt	0.9445691	0.2920586
B3	Ambu	Vt	0.9704344	0.7640233
B4	Ambu	Vt	0.9521169	0.4003706
B5	Ambu	Vt	0.9562907	0.4726586
B6	Ambu	Vt	0.9500205	0.3674006
C1	Ambu	Vt	0.8157929	0.0015005
C2	Ambu	Vt	0.9563414	0.4735902
C3	Ambu	Vt	0.9752468	0.8593357
D1	Ambu	Vt	0.9404193	0.2441940
D2	Ambu	Vt	0.9310081	0.1614694
D4	Ambu	Vt	0.8483739	0.0050067
E1	Ambu	Vt	0.8983364	0.0383853
E2	Ambu	Vt	0.8473579	0.0048152
A1	BBVM	Vt	0.9438042	0.2826459
A2	BBVM	Vt	0.9516862	0.3934131
A4	BBVM	Vt	0.9502268	0.3705454
A5	BBVM	Vt	0.9605632	0.5551620
A6	BBVM	Vt	0.9486037	0.3463922
B1	BBVM	Vt	0.8975144	0.0370522
B2	BBVM	Vt	0.8386378	0.0034588
B3	BBVM	Vt	0.9371899	0.2120723
B4	BBVM	Vt	0.9106300	0.0655223
B5	BBVM	Vt	0.9055316	0.0524223
B6	BBVM	Vt	0.9458374	0.3082802
C1	BBVM	Vt	0.7553150	0.0002002
C2	BBVM	Vt	0.9272886	0.1369205
C3	BBVM	Vt	0.8355624	0.0030829
D1	BBVM	Vt	0.9378436	0.2182352
D2	BBVM	Vt	0.9869821	0.9911646
D4	BBVM	Vt	0.9564415	0.4754314
E1	BBVM	Vt	0.9649777	0.6472707
E2	BBVM	Vt	0.9674180	0.6996922

- **Visualizing the differences between the BVM Types per each participant**

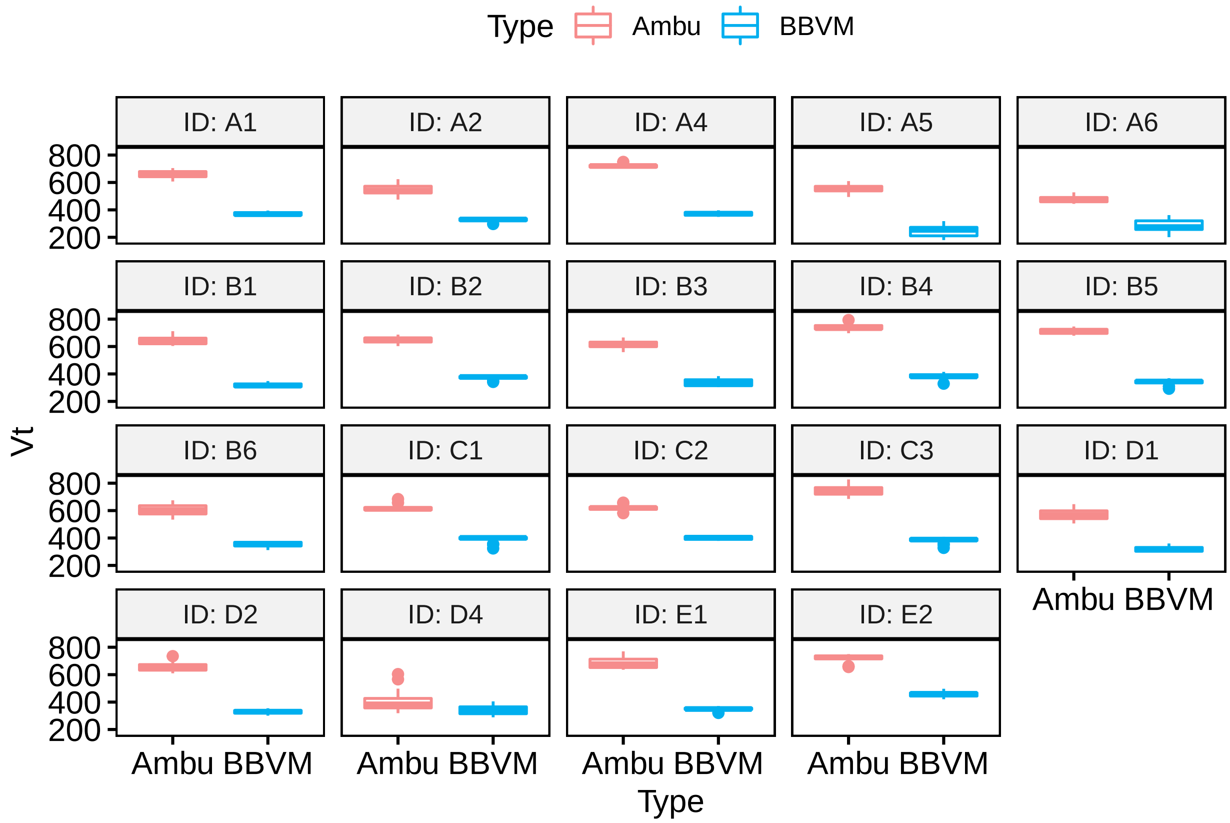

- **Pairwise T-test comparing the BVM Types by each participant**

ID	.y.	group1	group2	n1	n2	statistic	df	p
A1	Vt	Ambu	BBVM	20	20	41.006513	19	0.0000
A2	Vt	Ambu	BBVM	20	20	20.061175	19	0.0000
A4	Vt	Ambu	BBVM	20	20	72.544296	19	0.0000
A5	Vt	Ambu	BBVM	20	20	36.355830	19	0.0000
A6	Vt	Ambu	BBVM	20	20	17.203244	19	0.0000
B1	Vt	Ambu	BBVM	20	20	41.527881	19	0.0000
B2	Vt	Ambu	BBVM	20	20	43.517043	19	0.0000
B3	Vt	Ambu	BBVM	20	20	31.030473	19	0.0000
B4	Vt	Ambu	BBVM	20	20	66.744810	19	0.0000
B5	Vt	Ambu	BBVM	20	20	61.102027	19	0.0000
B6	Vt	Ambu	BBVM	20	20	24.364896	19	0.0000
C1	Vt	Ambu	BBVM	20	20	37.139340	19	0.0000
C2	Vt	Ambu	BBVM	20	20	37.427745	19	0.0000
C3	Vt	Ambu	BBVM	20	20	43.543198	19	0.0000
D1	Vt	Ambu	BBVM	20	20	29.619360	19	0.0000
D2	Vt	Ambu	BBVM	20	20	37.438769	19	0.0000
D4	Vt	Ambu	BBVM	20	20	2.945586	19	0.0083
E1	Vt	Ambu	BBVM	20	20	36.973700	19	0.0000

ID	.y.	group1	group2	n1	n2	statistic	df	p
E2	Vt	Ambu	BBVM	20	20	35.904215	19	0.0000

ANOVA approach for the comparisons

- **"Interaction" plot between the Type and the repeated measurements, Trial, on Vt**

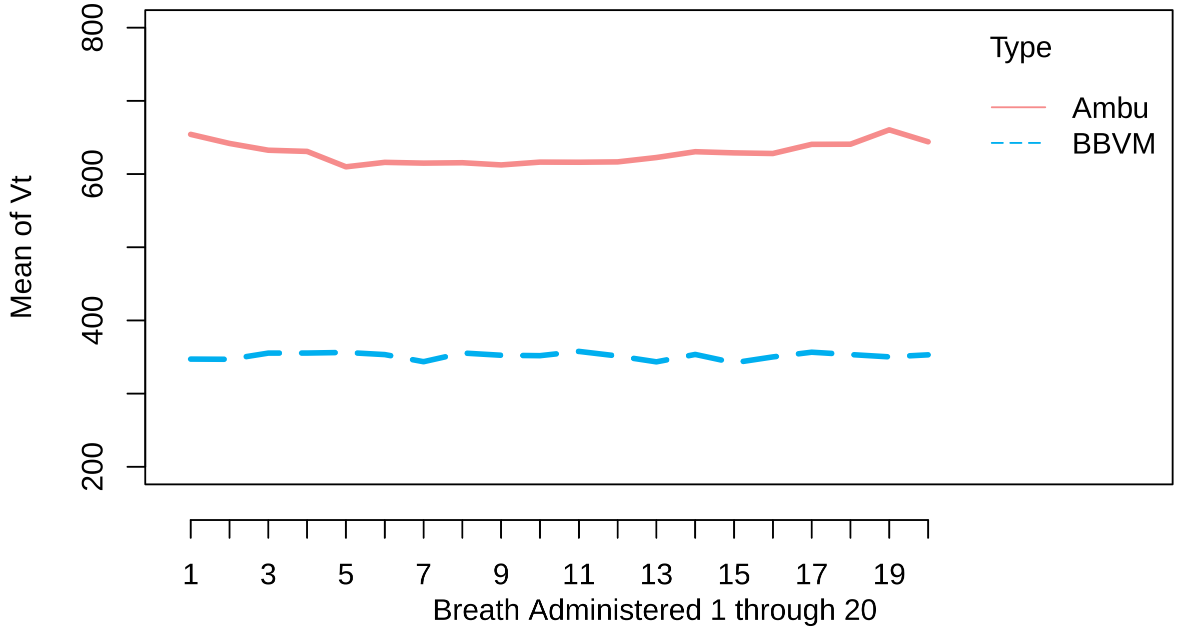

- **With Type only and Accounting for the random effects of the Participants.**

```
##
## Error: ID
##           Df  Sum Sq Mean Sq F value Pr(>F)
## Residuals 18 2650608  147256
##
## Error: Within
##            Df   Sum Sq  Mean Sq F value Pr(>F)
## Type        1 14600678 14600678    6728 <2e-16 ***
## Residuals 740  1605855     2170
## ---
## Signif. codes:  0 '***' 0.001 '**' 0.01 '*' 0.05 '.' 0.1 ' ' 1
```

- **With Type and Exp and Accounting for the random effects of the Participants (unbalanced case).**

```
##
## Error: ID
##           Df  Sum Sq Mean Sq F value Pr(>F)
## Exp        2  597818  298909    2.33  0.129
## Residuals 16 2052790  128299
##
## Error: Within
##            Df   Sum Sq  Mean Sq F value Pr(>F)
## Type        1 14600678 14600678    6728 <2e-16 ***
## Residuals 740  1605855     2170
## ---
## Signif. codes:  0 '***' 0.001 '**' 0.01 '*' 0.05 '.' 0.1 ' ' 1
```

- **With Type and Gender and Accounting for the random effects of the Participants (unbalanced case).**

```
##
## Error: ID
##           Df  Sum Sq Mean Sq F value Pr(>F)
## Gender     1  667143  667143   5.718 0.0286 *
## Residuals 17 1983465  116674
## ---
## Signif. codes:  0 '***' 0.001 '**' 0.01 '*' 0.05 '.' 0.1 ' ' 1
##
## Error: Within
##            Df   Sum Sq  Mean Sq F value Pr(>F)
## Type        1 14600678 14600678    6728 <2e-16 ***
## Residuals 740  1605855     2170
## ---
## Signif. codes:  0 '***' 0.001 '**' 0.01 '*' 0.05 '.' 0.1 ' ' 1
```

• With Type, Exp and Gender and Accounting for the random effects of the Participants (unbalanced case).

```
##
## Error: ID
##           Df  Sum Sq Mean Sq F value  Pr(>F)
## Gender     1  667143  667143   8.930 0.00919 **
## Exp        2  862877  431439   5.775 0.01381 *
## Residuals 15 1120588   74706
## ---
## Signif. codes:  0 '***' 0.001 '**' 0.01 '*' 0.05 '.' 0.1 ' ' 1
##
## Error: Within
##            Df   Sum Sq  Mean Sq F value Pr(>F)
## Type        1 14600678 14600678    6728 <2e-16 ***
## Residuals 740  1605855     2170
## ---
## Signif. codes:  0 '***' 0.001 '**' 0.01 '*' 0.05 '.' 0.1 ' ' 1
```

• 1-way Anova (Type) and Accounting for the random effects of the Participants– using LME.

```
## Loading required package: nlme

##
## Attaching package: 'nlme'

## The following object is masked from 'package:dplyr':
##
##     collapse

## Linear mixed-effects model fit by REML
##   Data: data0
##        AIC      BIC    logLik
##   8070.252 8088.775 -4031.126
##
## Random effects:
##  Formula: ~1 | ID
##         (Intercept) Residual
## StdDev:    60.22581 46.58406
##
## Fixed effects:  Vt ~ Type
##                  Value Std.Error  DF   t-value p-value
## (Intercept)  628.6789 14.021885 740  44.83555       0
## TypeBBVM    -277.2105  3.379563 740 -82.02555       0
##  Correlation:
##          (Intr)
## TypeBBVM -0.121
##
## Standardized Within-Group Residuals:
##        Min         Q1        Med         Q3        Max
## -4.1768976 -0.6457592  0.0173058  0.6117727  3.6199861
```

```
##
## Number of Observations: 760
## Number of Groups: 19
```

```
##             numDF denDF  F-value p-value
## (Intercept)     1   740 1239.548  <.0001
## Type            1   740 6728.191  <.0001
```

- **2-way Anova (Type+Exp) and Accounting for the random effects of the Participants– using NLME.**

```
##   ID Gender Exp Trial Size Type  Vt
## 1 A1      M  T1     1 19.5 Ambu 634
## 2 A1      M  T1     2 19.5 Ambu 644
## 3 A1      M  T1     3 19.5 Ambu 666
## 4 A1      M  T1     4 19.5 Ambu 687
## 5 A1      M  T1     5 19.5 Ambu 665
## 6 A1      M  T1     6 19.5 Ambu 668
```

```
## Linear mixed-effects model fit by REML
##   Data: data0
##       AIC      BIC    logLik
##   8051.65 8079.418 -4019.825
##
## Random effects:
##  Formula: ~1 | ID
##         (Intercept) Residual
## StdDev:    56.15365 46.58406
##
## Fixed effects:  Vt ~ Type + Exp
##                 Value Std.Error  DF   t-value p-value
## (Intercept)  610.1976  15.79826 740  38.62436  0.0000
## TypeBBVM    -277.2105   3.37956 740 -82.02555  0.0000
## ExpT2         49.9389  32.38210  16   1.54218  0.1426
## ExpT3         75.6952  43.01711  16   1.75965  0.0976
##  Correlation:
##          (Intr) TyBBVM ExpT2
## TypeBBVM -0.107
## ExpT2    -0.482  0.000
## ExpT3    -0.363  0.000  0.177
##
## Standardized Within-Group Residuals:
##         Min          Q1         Med          Q3         Max
## -4.17564775 -0.64999781  0.02299579  0.60603017  3.62123597
##
## Number of Observations: 760
## Number of Groups: 19
```

```
##             numDF denDF  F-value p-value
## (Intercept)     1   740 1422.695  <.0001
## Type            1   740 6728.191  <.0001
## Exp             2    16    2.330  0.1294
```

• **2-way Anova (Type+Gender) and Accounting for the random effects of the Participants– using NLME.**

```
## Linear mixed-effects model fit by REML
##   Data: data0
##        AIC      BIC   logLik
##   8058.779 8081.926 -4024.39
##
## Random effects:
##  Formula: ~1 | ID
##         (Intercept) Residual
## StdDev:    53.50335 46.58406
##
## Fixed effects:  Vt ~ Type + Gender
##                 Value Std.Error  DF   t-value p-value
## (Intercept)  603.4121 16.371452 740  36.85758  0.0000
## TypeBBVM    -277.2105  3.379563 740 -82.02555  0.0000
## GenderM       60.0088 25.095350  17   2.39123  0.0286
##  Correlation:
##          (Intr) TyBBVM
## TypeBBVM -0.103
## GenderM  -0.645  0.000
##
## Standardized Within-Group Residuals:
##        Min         Q1        Med         Q3        Max
## -4.1764962 -0.6421706  0.0205881  0.6132669  3.6203875
##
## Number of Observations: 760
## Number of Groups: 19
```

```
##             numDF denDF  F-value p-value
## (Intercept)     1   740 1564.447  <.0001
## Type            1   740 6728.191  <.0001
## Gender          1    17    5.718  0.0286
```

• **3-way Anova (Type) and Accounting for the random effects of the Participants– using NLME.**

```
## Linear mixed-effects model fit by REML
##   Data: data0
##        AIC      BIC  logLik
##   8035.799 8068.186 -4010.9
##
## Random effects:
##  Formula: ~1 | ID
##         (Intercept) Residual
## StdDev:    42.58397 46.58406
##
## Fixed effects:  Vt ~ Type + Exp + Gender
##                 Value Std.Error  DF   t-value p-value
## (Intercept)  570.9981  16.42139 740  34.77161  0.0000
## TypeBBVM    -277.2105   3.37956 740 -82.02555  0.0000
## ExpT2         89.1384  27.08723  15   3.29079  0.0050
```

```
## ExpT3         29.9625  35.28594  15   0.84913  0.4092
## GenderM       84.9321  24.04331  15   3.53246  0.0030
##  Correlation:
##          (Intr) TyBBVM ExpT2  ExpT3
## TypeBBVM -0.103
## ExpT2    -0.600  0.000
## ExpT3     0.000  0.000  0.000
## GenderM  -0.676  0.000  0.410 -0.367
##
## Standardized Within-Group Residuals:
##         Min          Q1         Med          Q3         Max
## -4.17682026 -0.64338401  0.02944334  0.61578507  3.62006346
##
## Number of Observations: 760
## Number of Groups: 19
```

```
##             numDF denDF  F-value p-value
## (Intercept)     1   740 2443.328  <.0001
## Type            1   740 6728.191  <.0001
## Exp             2    15    4.001  0.0405
## Gender          1    15   12.478  0.0030
```
