# Supplementary material for: Improved simulated ventilation with a novel tidal volume and peak inspiratory pressure controlling bag valve mask: A pilot study
Source: Resusc Plus. 2023 Jan 5;13:100350. doi: 10.1016/j.resplu.2022.100350 (PMC9841173; doi:10.1016/j.resplu.2022.100350)
Supplement: Supplementary data 3 [file mmc3.pdf]

Improved Ventilation with a Novel Tidal Volume and Peak Inspiratory Pressure Controlling Bag Valve Mask–A Pilot Study*

Supplemental Report #2

Jonathan Merrell, Adam Scott, Ryan Stambro, Amit Boukai and Dylan Cooper

Statistical Analysis of the Pediatric Mannequin Trial (on PIP, Vt and Rate)

• The raw Data

Sixteen participants (all EM physicians (either MD or DO) with active ACLS certification and a minimum one year of clinical experience) were recruited for this part of the study. Each participant was classified according to their years of experience ($T1$ = "less than 5 years", $T2$ = "between 5 to 10 years", $T3$ = "over 10 years") and according to their Gender (6 males and 10 females). Under a strict experimental protocol, the participant were asked to provide rescue ventilation to a simulated pediatrics patient (as a high-fidelity intubated Mannequin mannequin connected to an ASL 5000 machine). Each participant delivered 20 successive rescue breaths (trails) using a traditional BVM resuscitator ("Ambu") and then, after a brief pause, delivered 20 additional rescue breaths using a novel Butterfly BVM resuscitator ("BBVM"), and the tidal volume (V_t) and Peak Inspiratory Pressure (PIP) measurements, among others (Rate and MV), were recorded after each breath. The pediatric study included three experimental settings under the instructions to provide ventilation to a mannequin representing a 2yr old child who measures in the yellow Broselow weight group, has a pulse, but is breathing.

The Study considered three experimental settings:

- **Experimental Settings #1**: `Baseline:` The participant is instructed to provide appropriate ventilation to this patient.
- **Experimental Settings #2**: `Low PIP:` The participant is instructed to provide ventilation to this patient of an appropriate volume while keeping the peak inspiratory pressure at or below 20cmH2O.
- **Experimental Settings #3**: `High PIP:` The participant is instructed to provide ventilation to this patient of an appropriate volume while increasing the peak inspiratory pressure to something within 25 and 30cmH2O.

• The statistical procedures:

Below we provide descriptive statistics of the measured variables (V_t) and PIP, by the two types of resuscitators per each of the participants' groups (by Gender and by Years' Experience) and across the three experimental setting (`Condition`). As this was carried out as a Pilot Study, these groups were unbalanced. The V_t and the and PIP means under the two resuscitator types were compared by each group and by

*Supplemental Report to the *Improved Ventilation with a Novel Tidal Volume and Peak Inspiratory Pressure Controlling Bag Valve Mask–A Pilot Study*

participants, accounting also for the participants' (random) effect utilizing standard paired t-test as well as the the AOV (Analysis Of Variance) and the LME (Linear Mixed Effect) functions. Throughout, the significance level used for all testing as well as for Confidence Intervals was $\alpha = 0.01$.

• Setting up the Data Frame (Pediatric Mannequin Experiment)

```
## 'data.frame':    1920 obs. of  13 variables:
##  $ Participant.ID      : chr  "A5" "A5" "A5" "A5" ...
##  $ Test.Correct.Percent: int  93 93 93 93 93 93 93 93 93 93 ...
##  $ Test.Score          : int  13 13 13 13 13 13 13 13 13 13 ...
##  $ Gender              : chr  "F" "F" "F" "F" ...
##  $ Preferred.Hand      : chr  "R" "R" "R" "R" ...
##  $ Size                : num  15.5 15.5 15.5 15.5 15.5 15.5 15.5 15.5 15.5 15.5 ...
##  $ Yrs.Experience      : num  2.5 2.5 2.5 2.5 2.5 2.5 2.5 2.5 2.5 2.5 ...
##  $ Vt                  : int  125 132 142 143 143 131 137 131 142 147 ...
##  $ Rate                : num  14 14 14 14 14 14 14 14 14 14 ...
##  $ MV                  : int  1744 1842 1981 1995 1995 1828 1912 1828 1981 2051 ...
##  $ PIP                 : num  21.8 18.5 15.6 16.1 15.1 13.2 12.8 14.7 15.6 16.1 ...
##  $ STATE               : chr  "E1" "E1" "E1" "E1" ...
##  $ Type                : chr  "Ambu" "Ambu" "Ambu" "Ambu" ...
```

```
## 'data.frame':    1920 obs. of  10 variables:
##  $ ID       : Factor w/ 16 levels "A5","A6","B1",..: 1 1 1 1 1 1 1 1 1 1 ...
##  $ Gender   : Factor w/ 2 levels "F","M": 1 1 1 1 1 1 1 1 1 1 ...
##  $ Exp      : Factor w/ 3 levels "T1","T2","T3": 1 1 1 1 1 1 1 1 1 1 ...
##  $ Trial    : int  1 2 3 4 5 6 7 8 9 10 ...
##  $ Size     : num  15.5 15.5 15.5 15.5 15.5 15.5 15.5 15.5 15.5 15.5 ...
##  $ Rate     : num  14 14 14 14 14 14 14 14 14 14 ...
##  $ Vt       : int  125 132 142 143 143 131 137 131 142 147 ...
##  $ PIP      : num  21.8 18.5 15.6 16.1 15.1 13.2 12.8 14.7 15.6 16.1 ...
##  $ Condition: Factor w/ 3 levels "E1","E2","E3": 1 1 1 1 1 1 1 1 1 1 ...
##  $ Type     : Factor w/ 2 levels "Ambu","BBVM": 1 1 1 1 1 1 1 1 1 1 ...
```

• The Structure of the Pediatric Data

ID	Gender	Exp	Trial	Size	Rate	Vt	PIP	Condition	Type
A5	F	T1	1	15.5	14	125	21.8	E1	Ambu
A5	F	T1	2	15.5	14	132	18.5	E1	Ambu
A5	F	T1	3	15.5	14	142	15.6	E1	Ambu
A5	F	T1	4	15.5	14	143	16.1	E1	Ambu
A5	F	T1	5	15.5	14	143	15.1	E1	Ambu

• Changing the data frame from a wide format to a Long Style

```
## 'data.frame':    3840 obs. of  10 variables:
##  $ ID       : Factor w/ 16 levels "A5","A6","B1",..: 1 1 1 1 1 1 1 1 1 1 ...
##  $ Gender   : Factor w/ 2 levels "F","M": 1 1 1 1 1 1 1 1 1 1 ...
##  $ Exp      : Factor w/ 3 levels "T1","T2","T3": 1 1 1 1 1 1 1 1 1 1 ...
##  $ Trial    : int  1 2 3 4 5 6 7 8 9 10 ...
##  $ Size     : num  15.5 15.5 15.5 15.5 15.5 15.5 15.5 15.5 15.5 15.5 ...
```

```
##  $ Rate     : num  14 14 14 14 14 14 14 14 14 14 ...
##  $ Condition: Factor w/ 3 levels "E1","E2","E3": 1 1 1 1 1 1 1 1 1 1 ...
##  $ Type     : Factor w/ 2 levels "Ambu","BBVM": 1 1 1 1 1 1 1 1 1 1 ...
##  $ Method   : Factor w/ 2 levels "PIP","Vt": 2 2 2 2 2 2 2 2 2 2 ...
##  $ Y        : num  125 132 142 143 143 131 137 131 142 147 ...
```

Table 2: MySelection

ID	Gender	Exp	Trial	Size	Rate	Condition	Type	Method	Y
A5	F	T1	1	15.5	14	E1	Ambu	Vt	125
A5	F	T1	2	15.5	14	E1	Ambu	Vt	132
A5	F	T1	3	15.5	14	E1	Ambu	Vt	142
A5	F	T1	4	15.5	14	E1	Ambu	Vt	143
A5	F	T1	5	15.5	14	E1	Ambu	Vt	143
A5	F	T1	6	15.5	14	E1	Ambu	Vt	131

Analysis For Vt

- **Summary statistics for Vt by the two BVM types**

Condition	Type	variable	n	min	max	median	iqr	mean	sd	se	ci	cv
Baseline	Ambu	Vt	320	48	313	171.5	55.5	180.697	57.472	3.213	6.321	0.3180573
Low PIP	Ambu	Vt	320	41	233	134.0	51.0	136.009	41.021	2.293	4.512	0.3016050
High PIP	Ambu	Vt	320	46	329	176.5	62.5	173.016	61.239	3.423	6.735	0.3539499
Baseline	BBVM	Vt	320	27	124	93.0	22.0	90.009	17.909	1.001	1.970	0.1989690
Low PIP	BBVM	Vt	320	30	115	70.0	21.0	71.184	15.906	0.889	1.749	0.2234491
High PIP	BBVM	Vt	320	46	127	82.5	27.0	83.722	18.893	1.056	2.078	0.2256635

- **Visualizing the Distrubution of Vt by the two BVM Types per each one of the Experimental Settings**

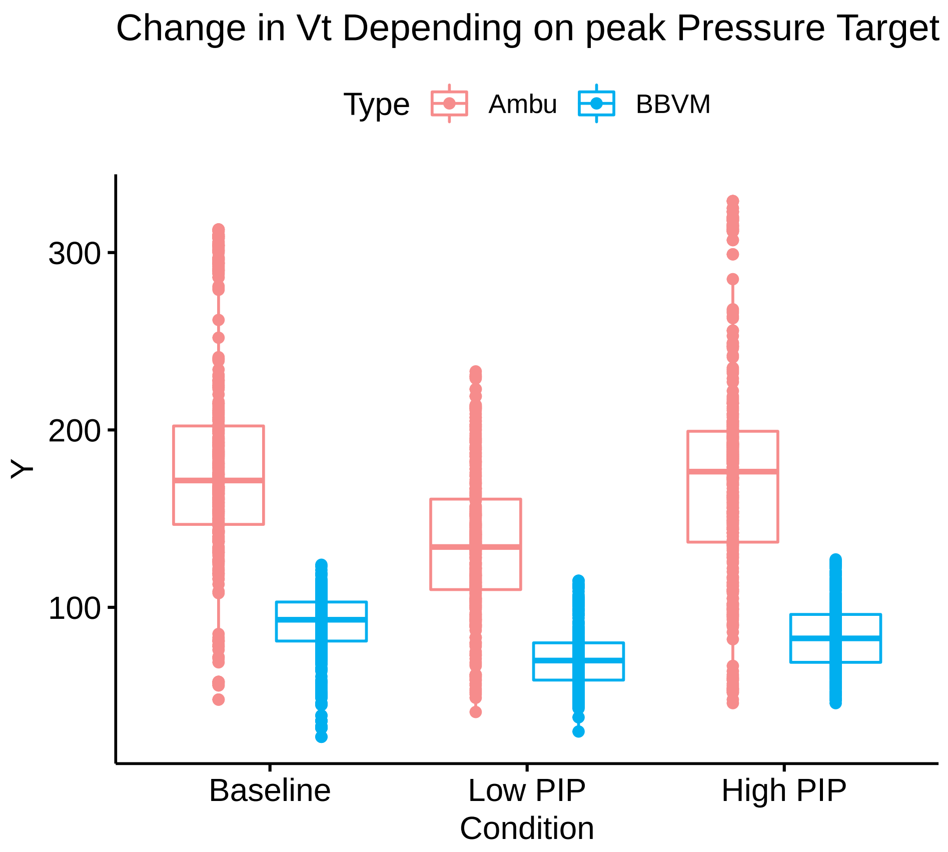

Condition	.y.	group1	group2	n1	n2	statistic	df	p
Baseline	Y	Ambu	BBVM	320	320	26.39964	319	0
Low PIP	Y	Ambu	BBVM	320	320	27.81035	319	0
High PIP	Y	Ambu	BBVM	320	320	26.78888	319	0

- **1-Way ANOVA (with Type) while accounting for the random effects of the Participants.**

```
##
## Error: ID
##           Df  Sum Sq Mean Sq F value Pr(>F)
## Residuals 15 1100674   73378
##
## Error: Within
##             Df  Sum Sq Mean Sq F value Pr(>F)
## Type         1 3196272 3196272    2527 <2e-16 ***
## Residuals 1903 2407182    1265
## ---
## Signif. codes:  0 '***' 0.001 '**' 0.01 '*' 0.05 '.' 0.1 ' ' 1
```

- **2-Way ANOVA (With Type+Condition) while accounting for the random effects of the Participants.**

```
##
## Error: ID
##           Df  Sum Sq Mean Sq F value Pr(>F)
## Residuals 15 1100674   73378
##
## Error: Within
##             Df  Sum Sq Mean Sq F value Pr(>F)
## Type         1 3196272 3196272  2962.9 <2e-16 ***
## Condition    2  356456  178228   165.2 <2e-16 ***
## Residuals 1901 2050727    1079
## ---
## Signif. codes:  0 '***' 0.001 '**' 0.01 '*' 0.05 '.' 0.1 ' ' 1
```

- **1-way ANOVA (with Type) and Accounting for the random effects of the Participants– using LME.**

```
## Loading required package: nlme

##
## Attaching package: 'nlme'

## The following object is masked from 'package:dplyr':
##
##     collapse

## Linear mixed-effects model fit by REML
##   Data: data1Vt
##        AIC      BIC    logLik
##   19225.54 19247.78 -9608.772
##
## Random effects:
##  Formula: ~1 | ID
##         (Intercept) Residual
```

```
## StdDev:    24.51417 35.56601
##
## Fixed effects:  Y ~ Type
##                 Value Std.Error   DF   t-value p-value
## (Intercept) 163.24062  6.235117 1903  26.18084       0
## TypeBBVM    -81.60208  1.623359 1903 -50.26744       0
##  Correlation:
##          (Intr)
## TypeBBVM -0.13
##
## Standardized Within-Group Residuals:
##         Min          Q1         Med          Q3         Max
## -2.82876052 -0.60605057  0.02055446  0.53406879  3.62731277
##
## Number of Observations: 1920
## Number of Groups: 16
```

```
##             numDF denDF   F-value p-value
## (Intercept)     1  1903  392.2629  <.0001
## Type            1  1903 2526.8153  <.0001
```

• 2-way Anova (Type+Condition) and Accounting for the random effects of the Participants– using LME.

```
## Linear mixed-effects model fit by REML
##   Data: data1Vt
##        AIC      BIC    logLik
##   18918.75 18952.09 -9453.373
##
## Random effects:
##  Formula: ~1 | ID
##         (Intercept) Residual
## StdDev:     24.5458 32.84451
##
## Fixed effects:  Y ~ Type + Condition
##                       Value Std.Error   DF   t-value p-value
## (Intercept)       176.15417  6.316916 1901  27.88610   0e+00
## TypeBBVM          -81.60208  1.499140 1901 -54.43259   0e+00
## ConditionLow PIP  -31.75625  1.836064 1901 -17.29583   0e+00
## ConditionHigh PIP  -6.98438  1.836064 1901  -3.80399   1e-04
##  Correlation:
##                   (Intr) TyBBVM CnLPIP
## TypeBBVM          -0.119
## ConditionLow PIP  -0.145  0.000
## ConditionHigh PIP -0.145  0.000  0.500
##
## Standardized Within-Group Residuals:
##        Min         Q1        Med         Q3        Max
## -3.4551732 -0.5412139  0.0212806  0.5386439  3.7468188
##
## Number of Observations: 1920
## Number of Groups: 16
```

```
##             numDF denDF   F-value p-value
## (Intercept)     1  1901  392.2629  <.0001
## Type            1  1901 2962.9073  <.0001
## Condition       2  1901  165.2152  <.0001
```

Analysis For PIP

- **Summary statistics for PIP by the two BVM types**

Condition	Type	variable	n	min	max	median	iqr	mean	sd	se	ci	cv
Baseline	Ambu	PIP	320	4.26	44.0	25.055	16.725	25.406	10.106	0.565	1.112	0.3977801
Low PIP	Ambu	PIP	320	10.40	23.7	16.350	3.425	16.448	2.465	0.138	0.271	0.1498662
High PIP	Ambu	PIP	300	16.60	37.9	26.500	3.200	26.465	3.009	0.174	0.342	0.1136973
Baseline	BBVM	PIP	320	3.80	35.5	18.900	15.700	20.280	8.322	0.465	0.915	0.4103550
Low PIP	BBVM	PIP	320	5.20	20.3	15.400	4.300	14.589	2.992	0.167	0.329	0.2050860
High PIP	BBVM	PIP	300	8.99	30.4	21.800	10.100	20.302	5.629	0.325	0.640	0.2772633

- **Visualizing the Distrubution of PIP by the two BVM Types per each one of the Experimental Settings**

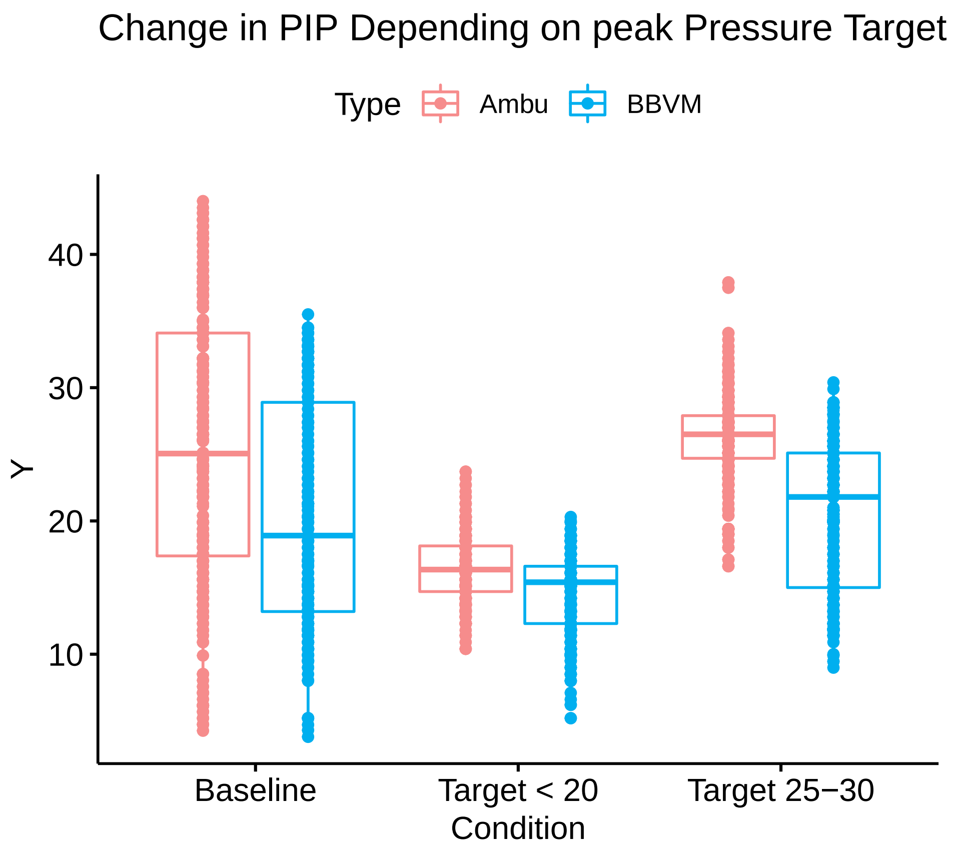

Condition	.y.	group1	group2	n1	n2	statistic	df	p
Baseline	Y	Ambu	BBVM	320	320	8.767199	319	0
Target < 20	Y	Ambu	BBVM	320	320	11.068784	319	0
Target 25-30	Y	Ambu	BBVM	320	320	19.785757	299	0

For *PIP*

• 1-Way ANOVA (with Type) while accounting for the random effects of the Participants.

```
##
## Error: ID
##           Df Sum Sq Mean Sq F value Pr(>F)
## Residuals 15  27910    1861
##
## Error: Within
##             Df Sum Sq Mean Sq F value Pr(>F)
## Type         1   8873    8873   237.1 <2e-16 ***
## Residuals 1863  69705      37
## ---
## Signif. codes:  0 '***' 0.001 '**' 0.01 '*' 0.05 '.' 0.1 ' ' 1
```

• 2-Way ANOVA (With Type+Condition) while accounting for the random effects of the Participants.

```
##
## Error: ID
##           Df Sum Sq Mean Sq F value Pr(>F)
## Condition  1    353   352.6   0.179  0.679
## Residuals 14  27557  1968.4
##
## Error: Within
##             Df Sum Sq Mean Sq F value Pr(>F)
## Type         1   8873    8873   369.5 <2e-16 ***
## Condition    2  25014   12507   520.8 <2e-16 ***
## Residuals 1861  44691      24
## ---
## Signif. codes:  0 '***' 0.001 '**' 0.01 '*' 0.05 '.' 0.1 ' ' 1
```

• 1-way ANOVA (with Type) and Accounting for the random effects of the Participants– using LME.

```
## Linear mixed-effects model fit by REML
##   Data: na.omit(data1PIP)
##        AIC      BIC    logLik
##   12211.81 12233.96 -6101.903
##
## Random effects:
##  Formula: ~1 | ID
##         (Intercept) Residual
## StdDev:    3.910205 6.116802
##
## Fixed effects:  Y ~ Type
##                 Value Std.Error   DF   t-value p-value
## (Intercept) 22.736167 0.9977968 1863  22.78637       0
## TypeBBVM    -4.344968 0.2821470 1863 -15.39966       0
```

```
##  Correlation:
##          (Intr)
## TypeBBVM -0.141
##
## Standardized Within-Group Residuals:
##         Min          Q1         Med          Q3         Max
## -2.25269823 -0.77398960 -0.07039409  0.68910845  2.60688316
##
## Number of Observations: 1880
## Number of Groups: 16
```

```
##             numDF denDF  F-value p-value
## (Intercept)     1  1863 433.3980  <.0001
## Type            1  1863 237.1495  <.0001
```

• 2-way Anova (Type+Condition) and Accounting for the random effects of the Participants– using LME.

```
## Linear mixed-effects model fit by REML
##   Data: na.omit(data1PIP)
##        AIC      BIC    logLik
##   11389.94 11423.17 -5688.972
##
## Random effects:
##  Formula: ~1 | ID
##         (Intercept) Residual
## StdDev:    3.990716 4.900476
##
## Fixed effects:  Y ~ Type + Condition
##                            Value Std.Error   DF   t-value p-value
## (Intercept)            25.015140 1.0225753 1861  24.46288  0.0000
## TypeBBVM               -4.344968 0.2260421 1861 -19.22194  0.0000
## ConditionTarget < 20   -7.323969 0.2739449 1861 -26.73519  0.0000
## ConditionTarget 25-30   0.763444 0.2806688 1861   2.72009  0.0066
##  Correlation:
##                       (Intr) TyBBVM CnT<20
## TypeBBVM              -0.111
## ConditionTarget < 20  -0.134  0.000
## ConditionTarget 25-30 -0.131  0.000  0.488
##
## Standardized Within-Group Residuals:
##         Min          Q1         Med          Q3         Max
## -2.96840372 -0.66917517 -0.03390792  0.59928268  2.80860467
##
## Number of Observations: 1880
## Number of Groups: 16
```

```
##             numDF denDF  F-value p-value
## (Intercept)     1  1861 419.4200  <.0001
## Type            1  1861 369.4830  <.0001
## Condition       2  1861 520.5518  <.0001
```

Analysis For *Rate*

- **Summary statistics for *Rate* by the two BVM types**

Condition	Type	variable	n	min	max	median	iqr	mean	sd	se	ci	cv
Baseline	Ambu	Rate	320	8.5	21.8	17.80	5.700	16.712	3.870	0.216	0.426	0.2315701
Target < 20	Ambu	Rate	320	8.9	30.0	18.95	6.925	18.538	5.128	0.287	0.564	0.2766210
Target 25-30	Ambu	Rate	320	10.7	35.3	20.40	7.575	21.100	6.801	0.380	0.748	0.3223223
Baseline	BBVM	Rate	320	9.4	26.7	17.15	6.850	17.419	4.409	0.246	0.485	0.2531144
Target < 20	BBVM	Rate	320	9.0	30.0	17.50	6.350	18.350	5.215	0.292	0.574	0.2841962
Target 25-30	BBVM	Rate	320	9.4	31.6	18.65	5.125	19.337	5.533	0.309	0.609	0.2861354

- **Visualizing the Distrubution of *Rate* by the two BVM Types per each one of the Experimental Settings**

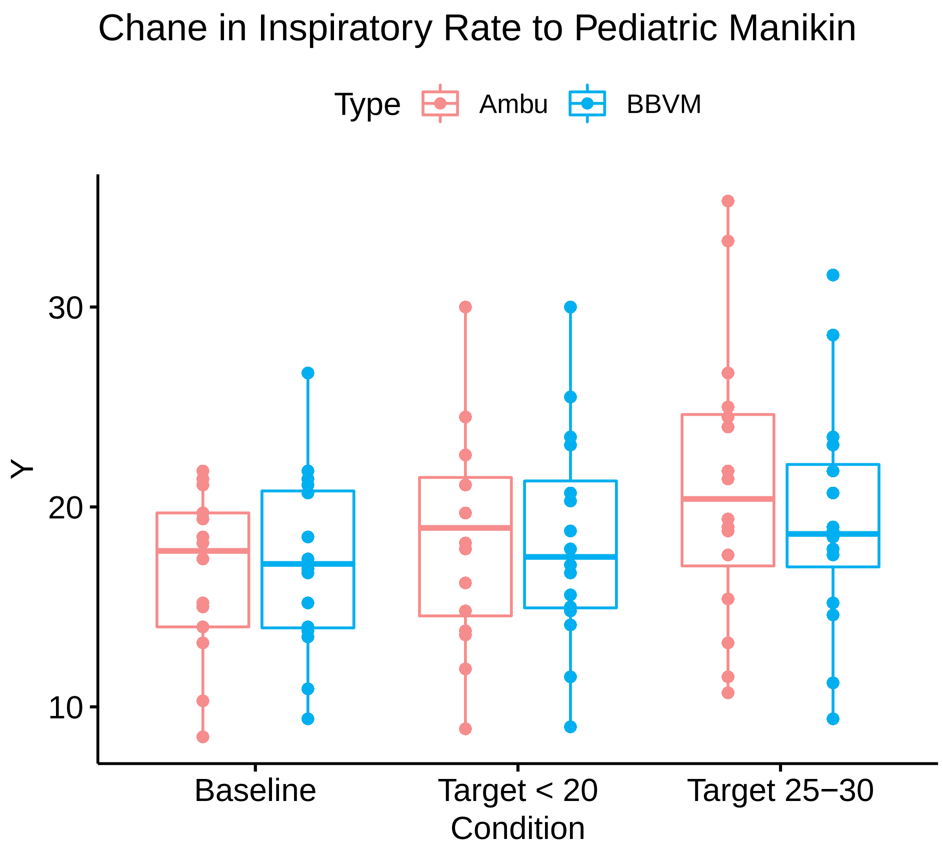

Condition	.y.	group1	group2	n1	n2	statistic	df	p
Baseline	Y	Ambu	BBVM	320	320	-3.7459329	319	2.13e-04
Target < 20	Y	Ambu	BBVM	320	320	0.7903527	319	4.30e-01
Target 25-30	Y	Ambu	BBVM	320	320	5.5275833	319	1.00e-07

For *Rate*

• 1-Way ANOVA (with Type) while accounting for the random effects of the Participants.

```
##
## Error: ID
##           Df Sum Sq Mean Sq F value Pr(>F)
## Residuals 15  37966    2531
##
## Error: Within
##             Df Sum Sq Mean Sq F value  Pr(>F)
## Type         1     83   82.50   8.581 0.00344 **
## Residuals 1903  18297    9.61
## ---
## Signif. codes:  0 '***' 0.001 '**' 0.01 '*' 0.05 '.' 0.1 ' ' 1
```

• 2-Way ANOVA (With Type+Condition) while accounting for the random effects of the Participants.

```
##
## Error: ID
##           Df Sum Sq Mean Sq F value Pr(>F)
## Residuals 15  37966    2531
##
## Error: Within
##             Df Sum Sq Mean Sq F value  Pr(>F)
## Type         1     83    82.5   10.39 0.00129 **
## Condition    2   3198  1599.2  201.35 < 2e-16 ***
## Residuals 1901  15098     7.9
## ---
## Signif. codes:  0 '***' 0.001 '**' 0.01 '*' 0.05 '.' 0.1 ' ' 1
```

• 1-way ANOVA (with Type) and Accounting for the random effects of the Participants– using LME.

```
## Linear mixed-effects model fit by REML
##   Data: na.omit(data2)
##        AIC      BIC    logLik
##   9460.835 9482.987 -4726.418
##
## Random effects:
##  Formula: ~1 | ID
##         (Intercept) Residual
## StdDev:    4.314219 2.921211
##
## Fixed effects:  Y ~ Type
##                 Value Std.Error   DF   t-value p-value
## (Intercept) 18.535677 1.0827753 1863 17.118673  0.0000
## TypeBBVM    -0.280851 0.1347454 1863 -2.084309  0.0373
##  Correlation:
```

```
##          (Intr)
## TypeBBVM -0.062
##
## Standardized Within-Group Residuals:
##         Min          Q1         Med          Q3         Max
## -2.03223949 -0.55985659 -0.03535392  0.44033217  4.60923170
##
## Number of Observations: 1880
## Number of Groups: 16
```

```
##             numDF denDF   F-value p-value
## (Intercept)     1  1863 289.74732  <.0001
## Type            1  1863   4.34434  0.0373
```

• 2-way Anova (Type+Condition) and Accounting for the random effects of the Participants– using LME.

```
## Linear mixed-effects model fit by REML
##   Data: na.omit(data2)
##       AIC      BIC    logLik
##   9179.35 9212.571 -4583.675
##
## Random effects:
##  Formula: ~1 | ID
##         (Intercept) Residual
## StdDev:    4.370131 2.703947
##
## Fixed effects:  Y ~ Type + Condition
##                           Value Std.Error   DF   t-value p-value
## (Intercept)           17.206051 1.0995184 1861 15.648715  0.0000
## TypeBBVM              -0.280851 0.1247238 1861 -2.251785  0.0245
## ConditionTarget < 20   1.378125 0.1511552 1861  9.117284  0.0000
## ConditionTarget 25-30  2.738968 0.1548822 1861 17.684210  0.0000
##  Correlation:
##                       (Intr) TyBBVM CnT<20
## TypeBBVM              -0.057
## ConditionTarget < 20  -0.069  0.000
## ConditionTarget 25-30 -0.067  0.000  0.488
##
## Standardized Within-Group Residuals:
##        Min         Q1        Med         Q3        Max
## -2.6275234 -0.5247514 -0.1069673  0.4704422  4.4738247
##
## Number of Observations: 1880
## Number of Groups: 16
```

```
##             numDF denDF   F-value p-value
## (Intercept)     1  1861 282.56648  <.0001
## Type            1  1861   5.07053  0.0245
## Condition       2  1861 156.52189  <.0001
```

- **2-way Anova and Accounting for the random effects of the Participants.**

```
##           Df Sum Sq Mean Sq F value Pr(>F)
## Residuals 15  37966    2531

##             Df Sum Sq Mean Sq F value  Pr(>F)
## Type         1     83    82.5   10.39 0.00129 **
## Condition    2   3198  1599.2  201.35 < 2e-16 ***
## Residuals 1901  15098     7.9
## ---
## Signif. codes:  0 '***' 0.001 '**' 0.01 '*' 0.05 '.' 0.1 ' ' 1
```
