# Supplementary material for: Improved simulated ventilation with a novel tidal volume and peak inspiratory pressure controlling bag valve mask: A pilot study
Source: Resusc Plus. 2023 Jan 5;13:100350. doi: 10.1016/j.resplu.2022.100350 (PMC9841173; doi:10.1016/j.resplu.2022.100350)
Supplement: Supplementary data 4 [file mmc4.pdf]

Comparing Ambu versus BBVM*

The Pediatric Mannequin Trial

Supplement #3, Analysis of PIP under the **Baseline** Condition

Summary: Experiment setting # 1, PIP Measurements

• Setting up the Data Frame (PIP Measurements)

```
## 'data.frame':    320 obs. of  6 variables:
##  $ ID    : Factor w/ 16 levels "A5","A6","B1",..: 1 1 1 1 1 1 1 1 1 1 ...
##  $ Gender: Factor w/ 2 levels "F","M": 1 1 1 1 1 1 1 1 1 1 ...
##  $ Exp   : Factor w/ 3 levels "T1","T2","T3": 1 1 1 1 1 1 1 1 1 1 ...
##  $ Trial : int  1 2 3 4 5 6 7 8 9 10 ...
##  $ Ambu  : num  21.8 18.5 15.6 16.1 15.1 13.2 12.8 14.7 15.6 16.1 ...
##  $ BBVM  : num  5.2 4.3 3.8 5.2 5.2 4.7 5.2 9.5 10.4 10.4 ...
```

• The Structure of the Pediatric Data

ID	Gender	Exp	Trial	Ambu	BBVM
A5	F	T1	1	21.8	5.2
A5	F	T1	2	18.5	4.3
A5	F	T1	3	15.6	3.8
A5	F	T1	4	16.1	5.2
A5	F	T1	5	15.1	5.2

• Changing the data frame from a wide format to a Long Style

```
## 'data.frame':    640 obs. of  6 variables:
##  $ ID    : Factor w/ 16 levels "A5","A6","B1",..: 1 1 1 1 1 1 1 1 1 1 ...
##  $ Gender: Factor w/ 2 levels "F","M": 1 1 1 1 1 1 1 1 1 1 ...
##  $ Exp   : Factor w/ 3 levels "T1","T2","T3": 1 1 1 1 1 1 1 1 1 1 ...
##  $ Trial : int  1 2 3 4 5 6 7 8 9 10 ...
##  $ Type  : Factor w/ 2 levels "Ambu","BBVM": 1 1 1 1 1 1 1 1 1 1 ...
##  $ PIP1  : num  21.8 18.5 15.6 16.1 15.1 13.2 12.8 14.7 15.6 16.1 ...
```

*Supplemental Report to the *Improved Ventilation with a Novel Tidal Volume and Peak Inspiratory Pressure Controlling Bag Valve Mask–A Pilot Study*

- **The numberof participants per each Gender by Experience group**

	F	M
T1	6	4
T2	4	0
T3	0	2

- **The sample sizes per each Gender by Experience group**

Exp	Gender	n	prop
T1	F	240	60
T1	M	160	40
T2	F	160	100
T3	M	80	100

- **Summary statistics for PIP_1 by the two BVM types (while ignoring all other factors)**

Type	variable	n	min	max	median	iqr	mean	sd	se	ci
Ambu	PIP1	320	4.26	44.0	25.055	16.725	25.406	10.106	0.565	1.112
BBVM	PIP1	320	3.80	35.5	18.900	15.700	20.280	8.322	0.465	0.915

- **Visualizing the Distrubution of PIP_1 by the two BVM Types (while ignoring all other factors)**

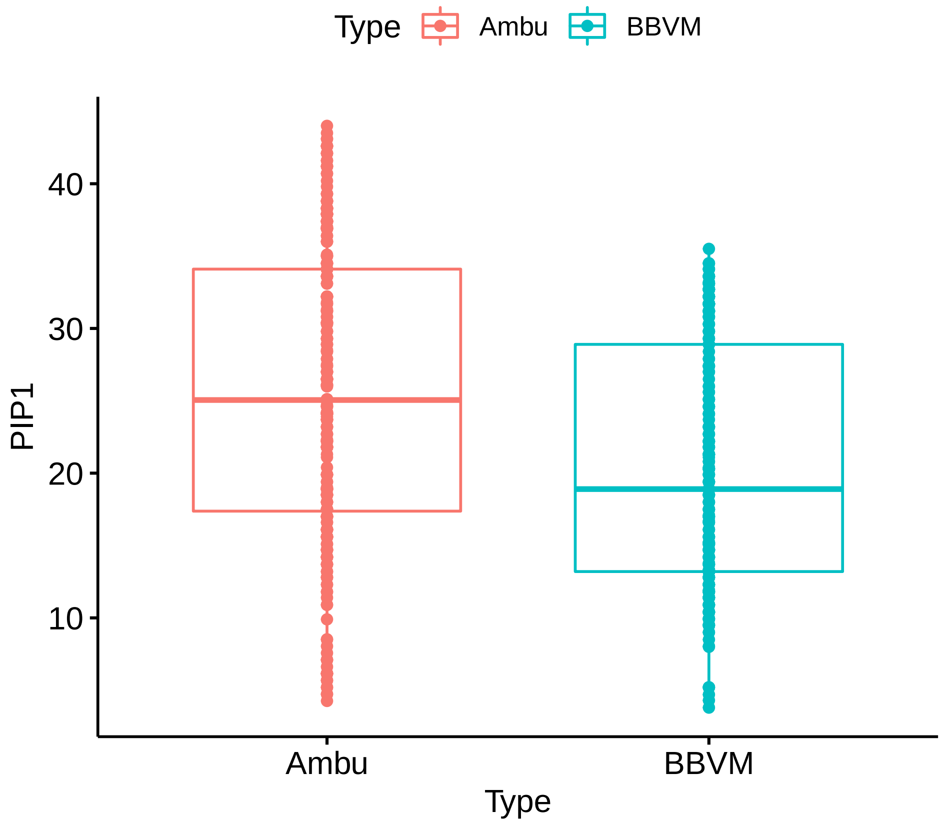

- **Summary statistics for PIP_1 by Gender and Type**

Gender	Type	variable	n	min	max	median	iqr	mean	sd	se	ci
F	Ambu	PIP1	200	4.26	40.2	24.10	17.500	23.907	10.080	0.713	1.406
M	Ambu	PIP1	120	13.70	44.0	27.00	18.900	27.903	9.686	0.884	1.751
F	BBVM	PIP1	200	3.80	34.5	15.60	9.000	17.361	6.971	0.493	0.972
M	BBVM	PIP1	120	9.00	35.5	28.15	12.925	25.143	8.139	0.743	1.471

- **Visualizing the Distrubution of PIP_1 by Type for each Gender**

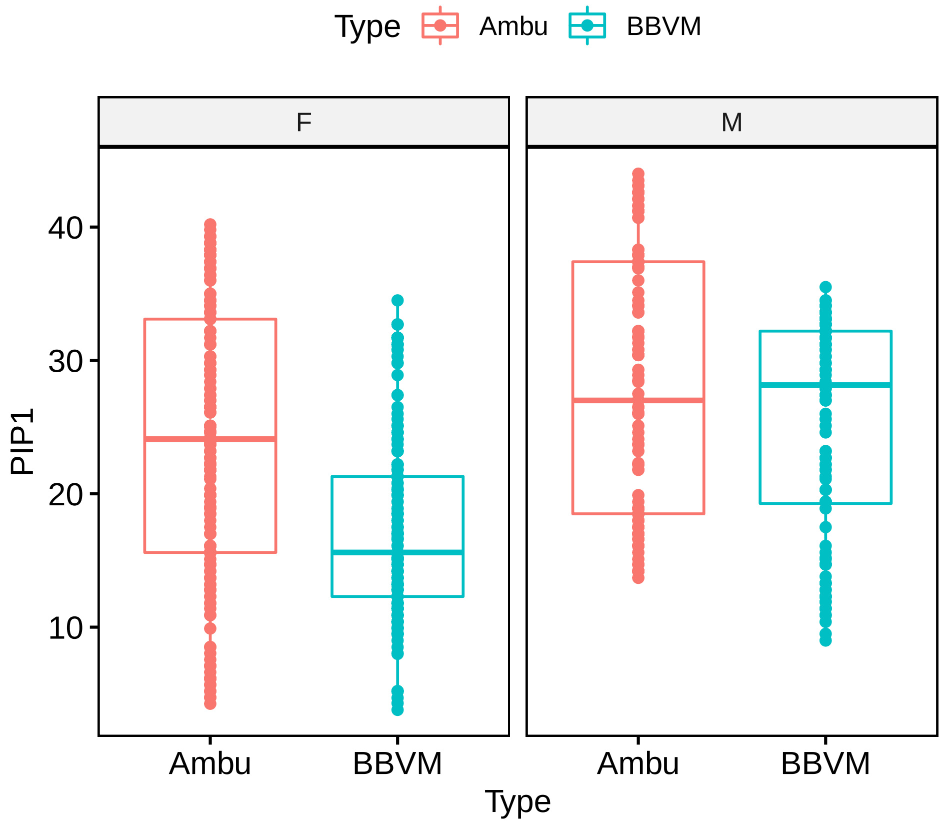

- **Summary statistics for PIP_1 by Type and Years of Expereince**

Exp	Type	variable	n	min	max	median	iqr	mean	sd	se	ci
T1	Ambu	PIP1	200	4.26	38.3	22.25	14.800	22.607	9.628	0.681	1.342
T2	Ambu	PIP1	80	17.00	40.2	30.75	11.550	30.492	6.348	0.710	1.413
T3	Ambu	PIP1	40	13.70	44.0	29.80	25.325	29.225	13.139	2.077	4.202
T1	BBVM	PIP1	200	3.80	35.5	15.60	11.800	18.492	8.063	0.570	1.124
T2	BBVM	PIP1	80	8.04	34.5	20.30	14.925	20.970	7.708	0.862	1.715
T3	BBVM	PIP1	40	11.40	33.6	30.55	7.225	27.835	6.204	0.981	1.984

- **Visualizing the Distrubution of PIP_1 by Type and Years of Expereince**

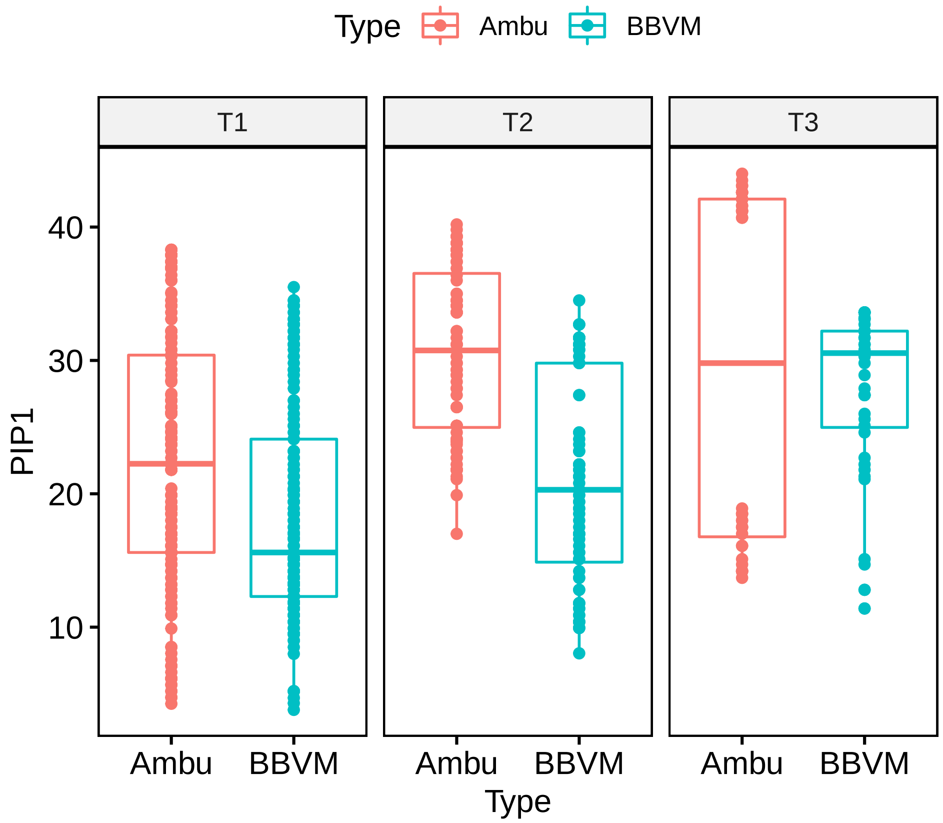

- **Summary statistics of PIP_1 by Participants and Type**

ID	Type	variable	n	min	max	median	iqr	mean	sd	se	ci
A5	Ambu	PIP1	20	12.80	21.80	15.600	1.750	15.825	2.032	0.454	0.951
A6	Ambu	PIP1	20	8.50	18.50	12.800	3.000	13.060	2.354	0.526	1.102
B1	Ambu	PIP1	20	18.90	29.80	24.805	2.925	24.811	2.807	0.628	1.314
B2	Ambu	PIP1	20	19.90	28.40	24.350	3.550	24.520	2.258	0.505	1.057
B3	Ambu	PIP1	20	30.30	38.30	36.900	3.375	35.775	2.397	0.536	1.122
B4	Ambu	PIP1	20	29.30	38.30	37.400	3.800	35.975	2.530	0.566	1.184
B5	Ambu	PIP1	20	22.30	37.00	30.800	2.175	30.970	3.461	0.774	1.620
B6	Ambu	PIP1	20	14.70	27.00	21.100	4.525	21.410	3.451	0.772	1.615
C1	Ambu	PIP1	20	13.70	18.90	16.550	3.800	16.330	1.846	0.413	0.864
C2	Ambu	PIP1	20	17.00	29.80	22.450	2.925	23.220	3.013	0.674	1.410
C3	Ambu	PIP1	20	23.20	34.50	26.500	4.025	27.300	3.425	0.766	1.603
D1	Ambu	PIP1	20	14.70	19.40	17.250	2.300	17.505	1.331	0.298	0.623
D2	Ambu	PIP1	20	28.90	36.90	33.600	3.300	32.930	2.346	0.525	1.098
D4	Ambu	PIP1	20	4.26	8.52	6.150	1.540	6.221	1.121	0.251	0.525
E1	Ambu	PIP1	20	36.40	40.20	38.550	0.725	38.520	0.879	0.197	0.412
E2	Ambu	PIP1	20	40.70	44.00	42.100	1.400	42.120	0.931	0.208	0.436
A5	BBVM	PIP1	20	3.80	12.80	9.250	4.825	8.165	2.756	0.616	1.290
A6	BBVM	PIP1	20	10.40	21.30	14.200	1.600	14.645	2.998	0.670	1.403

ID	Type	variable	n	min	max	median	iqr	mean	sd	se	ci
B1	BBVM	PIP1	20	17.00	28.90	24.100	3.800	23.240	3.037	0.679	1.421
B2	BBVM	PIP1	20	23.20	32.70	31.200	2.400	30.330	2.276	0.509	1.065
B3	BBVM	PIP1	20	12.30	20.80	16.350	3.050	16.140	2.335	0.522	1.093
B4	BBVM	PIP1	20	28.40	35.50	33.100	1.900	32.965	1.502	0.336	0.703
B5	BBVM	PIP1	20	9.00	15.60	12.300	2.025	12.310	1.759	0.393	0.823
B6	BBVM	PIP1	20	10.40	20.40	15.200	3.225	15.220	2.607	0.583	1.220
C1	BBVM	PIP1	20	11.40	33.60	25.100	6.900	23.840	6.394	1.430	2.993
C2	BBVM	PIP1	20	29.80	34.50	31.700	0.500	31.635	0.999	0.223	0.467
C3	BBVM	PIP1	20	14.20	21.80	17.000	3.050	17.595	2.121	0.474	0.993
D1	BBVM	PIP1	20	12.30	27.00	19.400	3.350	19.585	3.392	0.759	1.588
D2	BBVM	PIP1	20	8.04	19.40	11.150	1.650	11.593	2.299	0.514	1.076
D4	BBVM	PIP1	20	9.46	15.60	12.050	2.425	12.325	1.648	0.368	0.771
E1	BBVM	PIP1	20	18.50	29.80	22.200	2.925	23.055	2.727	0.610	1.276
E2	BBVM	PIP1	20	25.10	33.60	32.200	1.550	31.830	2.126	0.475	0.995

- **Visualizing the Distrubution of PIP_1 by Participants and Type**

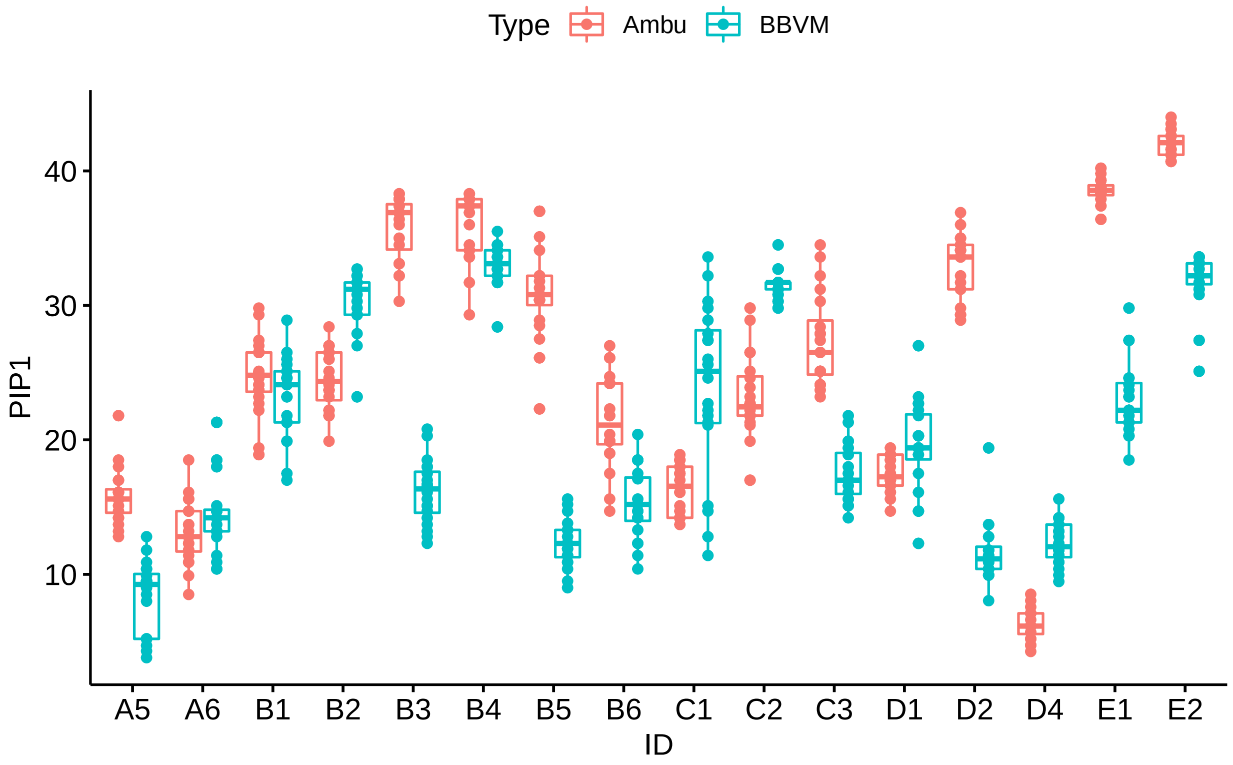

- **Basic test of Normality (Shapiro's Test applied to each Paricipant by Type)**

ID	Type	variable	statistic	p
A5	Ambu	PIP1	0.9088892	0.0607059
A6	Ambu	PIP1	0.9849945	0.9815148

ID	Type	variable	statistic	p
B1	Ambu	PIP1	0.9626733	0.5985275
B2	Ambu	PIP1	0.9620100	0.5847426
B3	Ambu	PIP1	0.8677365	0.0107234
B4	Ambu	PIP1	0.8210114	0.0018088
B5	Ambu	PIP1	0.9485407	0.3454813
B6	Ambu	PIP1	0.9606513	0.5569416
C1	Ambu	PIP1	0.8724515	0.0129780
C2	Ambu	PIP1	0.9358861	0.2002696
C3	Ambu	PIP1	0.8987849	0.0391336
D1	Ambu	PIP1	0.9283950	0.1438094
D2	Ambu	PIP1	0.9336579	0.1815411
D4	Ambu	PIP1	0.9682575	0.7177344
E1	Ambu	PIP1	0.9652423	0.6529303
E2	Ambu	PIP1	0.9527663	0.4110412
A5	BBVM	PIP1	0.9067677	0.0553267
A6	BBVM	PIP1	0.8725457	0.0130278
B1	BBVM	PIP1	0.9524367	0.4055988
B2	BBVM	PIP1	0.8293094	0.0024461
B3	BBVM	PIP1	0.9763081	0.8781222
B4	BBVM	PIP1	0.9033570	0.0476904
B5	BBVM	PIP1	0.9814685	0.9516176
B6	BBVM	PIP1	0.9692317	0.7385720
C1	BBVM	PIP1	0.9447175	0.2939173
C2	BBVM	PIP1	0.8942377	0.0322005
C3	BBVM	PIP1	0.9597573	0.5390128
D1	BBVM	PIP1	0.9597902	0.5396667
D2	BBVM	PIP1	0.8077043	0.0011282
D4	BBVM	PIP1	0.9704170	0.7636577
E1	BBVM	PIP1	0.9325693	0.1730168
E2	BBVM	PIP1	0.7333737	0.0001025

- **Visualizing the differences between the BVM Types per each participant**

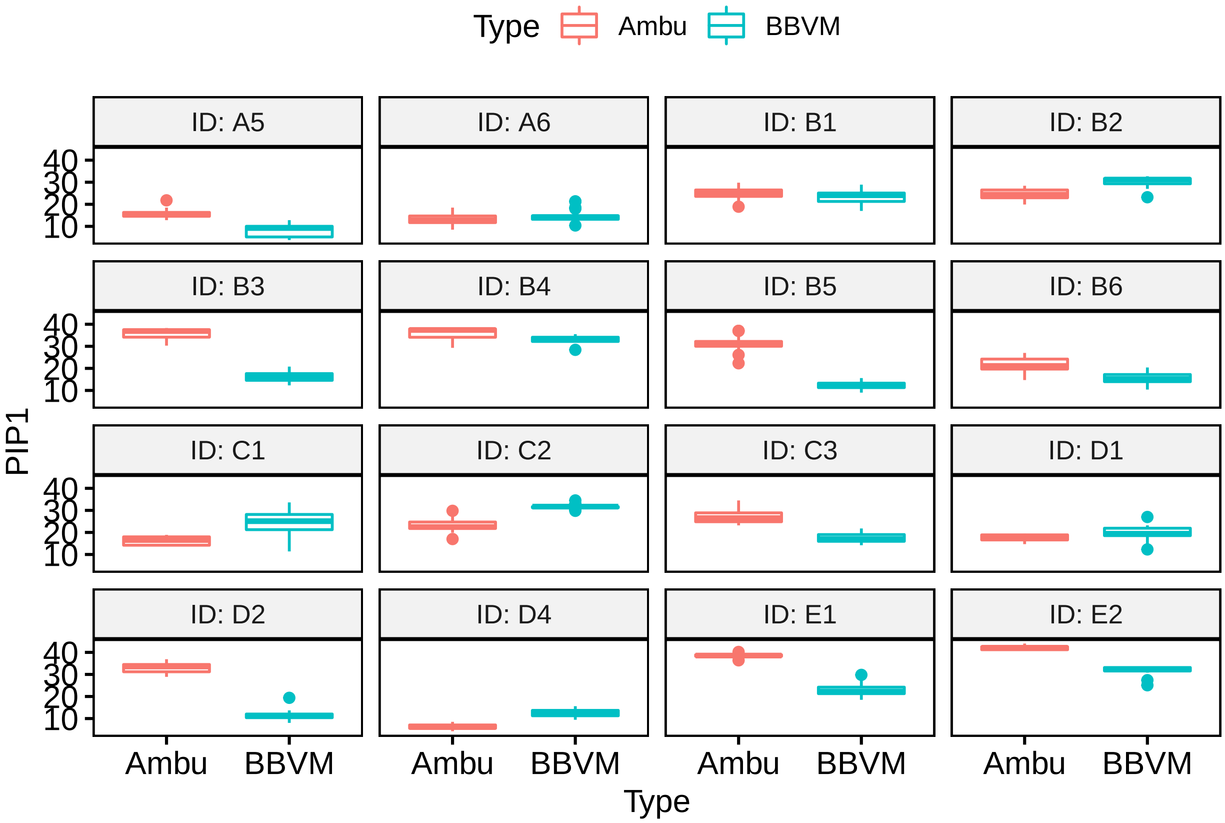

- **Pairwise T-test comparing the BVM Types by each participant**

ID	.y.	group1	group2	n1	n2	statistic	df	p
A5	PIP1	Ambu	BBVM	20	20	9.659412	19	0.00e+00
A6	PIP1	Ambu	BBVM	20	20	-1.851284	19	7.97e-02
B1	PIP1	Ambu	BBVM	20	20	1.454868	19	1.62e-01
B2	PIP1	Ambu	BBVM	20	20	-9.297237	19	0.00e+00
B3	PIP1	Ambu	BBVM	20	20	22.205848	19	0.00e+00
B4	PIP1	Ambu	BBVM	20	20	4.397707	19	3.09e-04
B5	PIP1	Ambu	BBVM	20	20	19.926577	19	0.00e+00
B6	PIP1	Ambu	BBVM	20	20	5.170357	19	5.44e-05
C1	PIP1	Ambu	BBVM	20	20	-5.429566	19	3.07e-05
C2	PIP1	Ambu	BBVM	20	20	-12.937474	19	0.00e+00
C3	PIP1	Ambu	BBVM	20	20	9.441701	19	0.00e+00
D1	PIP1	Ambu	BBVM	20	20	-3.165468	19	5.09e-03
D2	PIP1	Ambu	BBVM	20	20	26.074617	19	0.00e+00
D4	PIP1	Ambu	BBVM	20	20	-15.357700	19	0.00e+00
E1	PIP1	Ambu	BBVM	20	20	24.335280	19	0.00e+00
E2	PIP1	Ambu	BBVM	20	20	21.351586	19	0.00e+00

ANOVA approach for the comparisons

- **"Interaction" plot between the Type and the repeated measurements, Trial, on Vt**

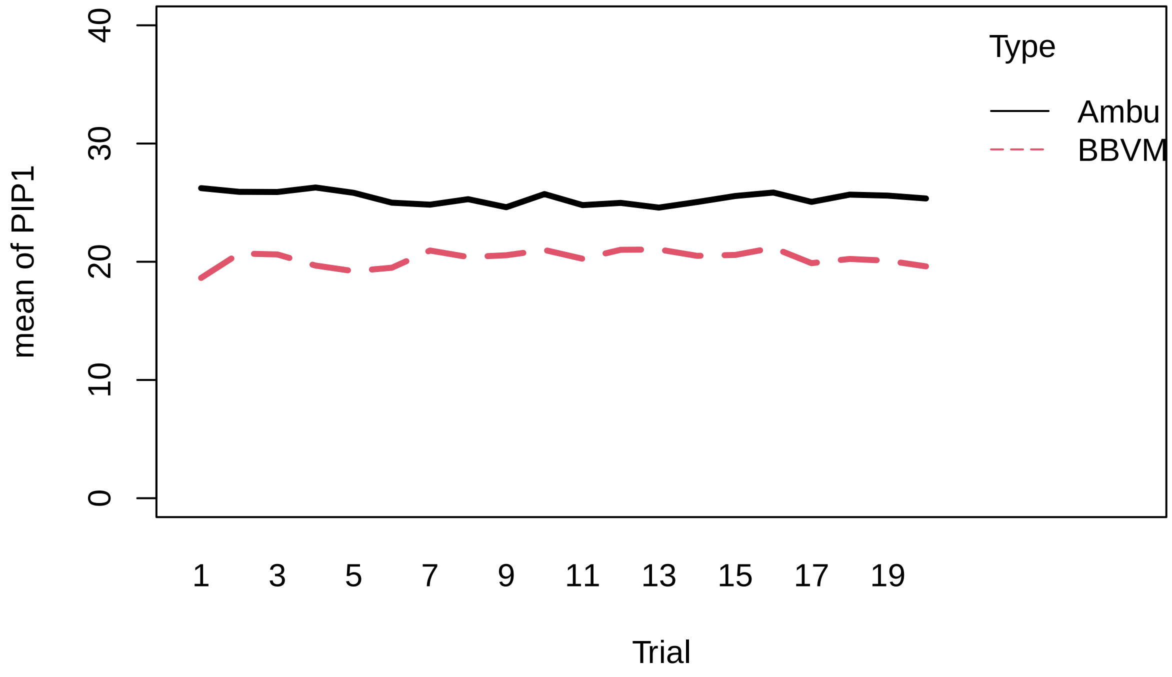

- **With Type only and also accounting for the random effects of the Participants.**

```
##
## Call:
## aov(formula = PIP1 ~ Type + Error(ID), data = data0)
##
## Grand Mean: 22.84266
##
## Stratum 1: ID
##
## Terms:
##                 Residuals
## Sum of Squares   35291.84
## Deg. of Freedom        15
##
## Residual standard error: 48.50556
##
## Stratum 2: Within
##
```

```
## Terms:
##                      Type Residuals
## Sum of Squares   4204.448 19384.218
## Deg. of Freedom         1       623
##
## Residual standard error: 5.578021
## Estimated effects are balanced
```

```
##           Df Sum Sq Mean Sq F value Pr(>F)
## Residuals 15  35292    2353
```

```
##            Df Sum Sq Mean Sq F value Pr(>F)
## Type        1   4204    4204   135.1 <2e-16 ***
## Residuals 623  19384      31
## ---
## Signif. codes:  0 '***' 0.001 '**' 0.01 '*' 0.05 '.' 0.1 ' ' 1
```

- **With Type and Exp and also accounting for the random effects of the Participants (unbalanced case).**

```
##
## Call:
## aov(formula = PIP1 ~ Type + Exp + Error(ID), data = data0)
##
## Grand Mean: 22.84266
##
## Stratum 1: ID
##
## Terms:
##                       Exp Residuals
## Sum of Squares   6025.259 29266.583
## Deg. of Freedom         2        13
##
## Residual standard error: 47.44761
## Estimated effects may be unbalanced
##
## Stratum 2: Within
##
## Terms:
##                      Type Residuals
## Sum of Squares   4204.448 19384.218
## Deg. of Freedom         1       623
##
## Residual standard error: 5.578021
## Estimated effects are balanced
```

```
##           Df Sum Sq Mean Sq F value Pr(>F)
## Exp        2   6025    3013   1.338  0.296
## Residuals 13  29267    2251
```

```
##            Df Sum Sq Mean Sq F value Pr(>F)
## Type        1   4204    4204   135.1 <2e-16 ***
```

```
## Residuals 623  19384      31
## ---
## Signif. codes:  0 '***' 0.001 '**' 0.01 '*' 0.05 '.' 0.1 ' ' 1
```

• **With Type and Gender also accounting for the random effects of the Participants (unbalanced case).**

```
##
## Call:
## aov(formula = PIP1 ~ Type + Gender + Error(ID), data = data0)
##
## Grand Mean: 22.84266
##
## Stratum 1: ID
##
## Terms:
##                    Gender Residuals
## Sum of Squares   5202.195 30089.647
## Deg. of Freedom         1        14
##
## Residual standard error: 46.36012
## Estimated effects are balanced
##
## Stratum 2: Within
##
## Terms:
##                      Type Residuals
## Sum of Squares   4204.448 19384.218
## Deg. of Freedom         1       623
##
## Residual standard error: 5.578021
## Estimated effects are balanced
```

```
##           Df Sum Sq Mean Sq F value Pr(>F)
## Gender     1   5202    5202    2.42  0.142
## Residuals 14  30090    2149
```

```
##            Df Sum Sq Mean Sq F value Pr(>F)
## Type        1   4204    4204   135.1 <2e-16 ***
## Residuals 623  19384      31
## ---
## Signif. codes:  0 '***' 0.001 '**' 0.01 '*' 0.05 '.' 0.1 ' ' 1
```

- **With Type, Exp and Gender also accounting for the random effects of the Participants (unbalanced case).**

```
##
## Call:
## aov(formula = PIP1 ~ Type + Gender + Exp + Error(ID), data = data0)
##
## Grand Mean: 22.84266
##
## Stratum 1: ID
##
## Terms:
##                    Gender       Exp Residuals
## Sum of Squares   5202.195  7410.368 22679.279
## Deg. of Freedom         1         2        12
##
## Residual standard error: 43.47344
## Estimated effects may be unbalanced
##
## Stratum 2: Within
##
## Terms:
##                      Type Residuals
## Sum of Squares   4204.448 19384.218
## Deg. of Freedom         1       623
##
## Residual standard error: 5.578021
## Estimated effects are balanced
```

```
##           Df Sum Sq Mean Sq F value Pr(>F)
## Gender     1   5202    5202   2.753  0.123
## Exp        2   7410    3705   1.960  0.183
## Residuals 12  22679    1890
```

```
##            Df Sum Sq Mean Sq F value Pr(>F)
## Type        1   4204    4204   135.1 <2e-16 ***
## Residuals 623  19384      31
## ---
## Signif. codes:  0 '***' 0.001 '**' 0.01 '*' 0.05 '.' 0.1 ' ' 1
```
