# Supplementary material for: Improved simulated ventilation with a novel tidal volume and peak inspiratory pressure controlling bag valve mask: A pilot study
Source: Resusc Plus. 2023 Jan 5;13:100350. doi: 10.1016/j.resplu.2022.100350 (PMC9841173; doi:10.1016/j.resplu.2022.100350)
Supplement: Supplementary data 5 [file mmc5.pdf]

Comparing Ambu versus BBVM*

The Pediatric Mannequin Trial

Supplement #4, Analysis of Vt under the **Baseline** Condition

Summary: Experiment setting # 1, Vt Measurements

• Setting up the Data Frame (Vt Measurements)

```
## 'data.frame':    320 obs. of  6 variables:
##  $ ID    : Factor w/ 16 levels "A5","A6","B1",..: 1 1 1 1 1 1 1 1 1 1 ...
##  $ Gender: Factor w/ 2 levels "F","M": 1 1 1 1 1 1 1 1 1 1 ...
##  $ Exp   : Factor w/ 3 levels "T1","T2","T3": 1 1 1 1 1 1 1 1 1 1 ...
##  $ Trial : int  1 2 3 4 5 6 7 8 9 10 ...
##  $ Ambu  : int  125 132 142 143 143 131 137 131 142 147 ...
##  $ BBVM  : int  50 39 32 27 36 33 32 36 64 65 ...
```

• The Structure of the Pediatric Data

ID	Gender	Exp	Trial	Ambu	BBVM
A5	F	T1	1	125	50
A5	F	T1	2	132	39
A5	F	T1	3	142	32
A5	F	T1	4	143	27
A5	F	T1	5	143	36

• Changing the data frame from a wide format to a Long Style

```
## 'data.frame':    640 obs. of  6 variables:
##  $ ID    : Factor w/ 16 levels "A5","A6","B1",..: 1 1 1 1 1 1 1 1 1 1 ...
##  $ Gender: Factor w/ 2 levels "F","M": 1 1 1 1 1 1 1 1 1 1 ...
##  $ Exp   : Factor w/ 3 levels "T1","T2","T3": 1 1 1 1 1 1 1 1 1 1 ...
##  $ Trial : int  1 2 3 4 5 6 7 8 9 10 ...
##  $ Type  : Factor w/ 2 levels "Ambu","BBVM": 1 1 1 1 1 1 1 1 1 1 ...
##  $ Vt1   : int  125 132 142 143 143 131 137 131 142 147 ...
```

*Supplemental Report to the *Improved Ventilation with a Novel Tidal Volume and Peak Inspiratory Pressure Controlling Bag Valve Mask–A Pilot Study*

- **The numberof participants per each Gender by Experience group**

	F	M
T1	6	4
T2	4	0
T3	0	2

- **The sample sizes per each Gender by Experience group**

Exp	Gender	n	prop
T1	F	240	60
T1	M	160	40
T2	F	160	100
T3	M	80	100

- **Summary statistics for Vt_1 by the two BVM types (while ignoring all other factors)**

Type	variable	n	min	max	median	iqr	mean	sd	se	ci
Ambu	Vt1	320	48	313	171.5	55.5	180.697	57.472	3.213	6.321
BBVM	Vt1	320	27	124	93.0	22.0	90.009	17.909	1.001	1.970

- **Visualizing the Distrubution of Vt_1 by the two BVM Types (while ignoring all other factors)**

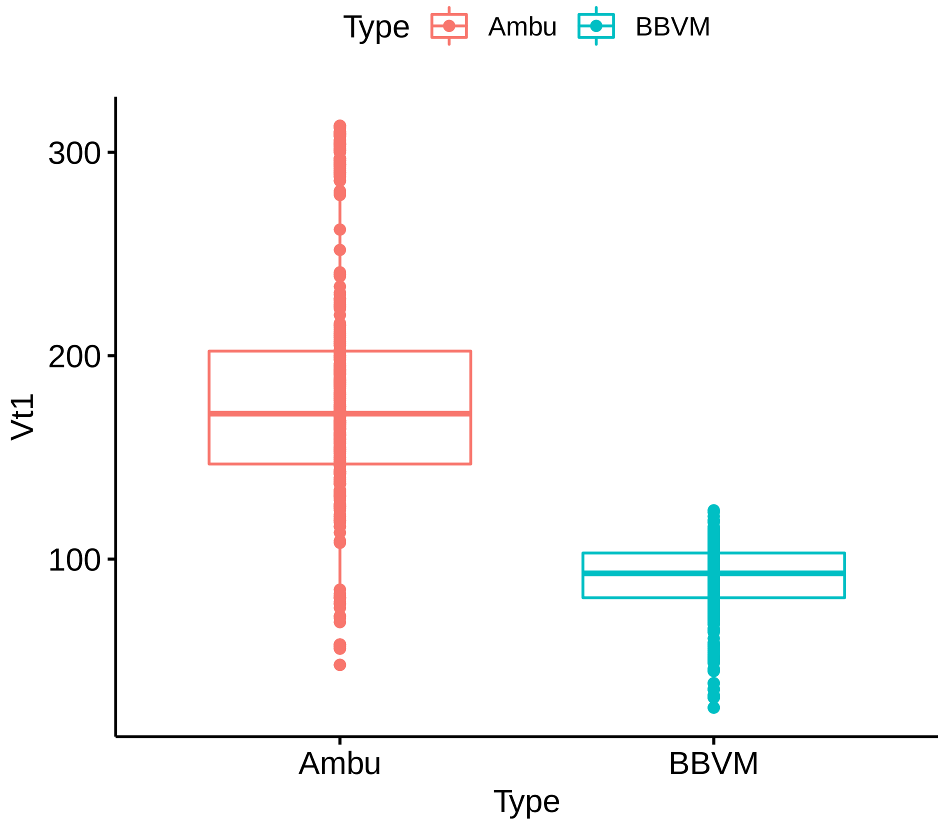

- **Summary statistics for Vt_1 by Gender and Type**

Gender	Type	variable	n	min	max	median	iqr	mean	sd	se	ci
F	Ambu	Vt1	200	48	313	167.0	62.50	175.670	59.242	4.189	8.261
M	Ambu	Vt1	120	113	308	180.5	45.75	189.075	53.593	4.892	9.687
F	BBVM	Vt1	200	27	113	87.0	21.00	84.985	18.543	1.311	2.586
M	BBVM	Vt1	120	68	124	100.5	17.00	98.383	13.100	1.196	2.368

- **Visualizing the Distrubution of Vt_1 by Type for each Gender**

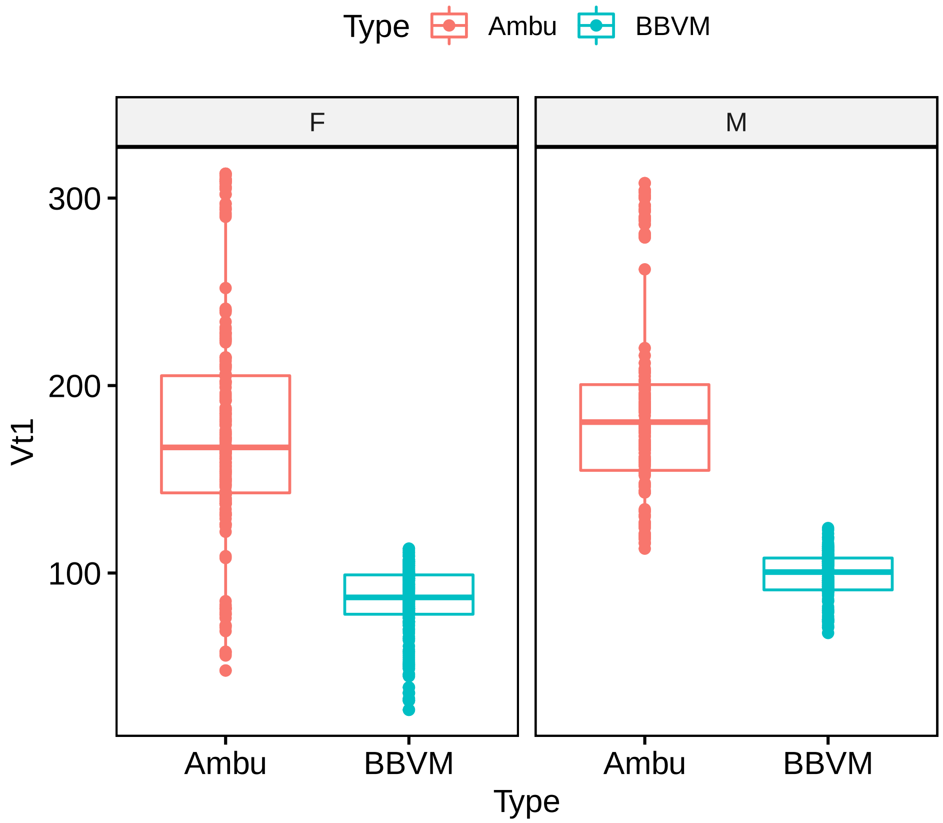

- **Summary statistics for Vt_1 by Type and Years of Expereince**

Exp	Type	variable	n	min	max	median	iqr	mean	sd	se	ci
T1	Ambu	Vt1	200	48	308	172.5	57.75	175.970	56.999	4.030	7.948
T2	Ambu	Vt1	80	139	313	190.0	75.00	210.662	56.737	6.343	12.626
T3	Ambu	Vt1	40	113	205	143.0	36.75	144.400	24.729	3.910	7.909
T1	BBVM	Vt1	200	27	124	92.0	26.50	87.630	20.430	1.445	2.849
T2	BBVM	Vt1	80	65	113	91.5	21.00	91.262	11.985	1.340	2.667
T3	BBVM	Vt1	40	85	116	96.0	14.00	99.400	8.944	1.414	2.860

- **Visualizing the Distrubution of Vt_1 by Type and Years of Expereince**

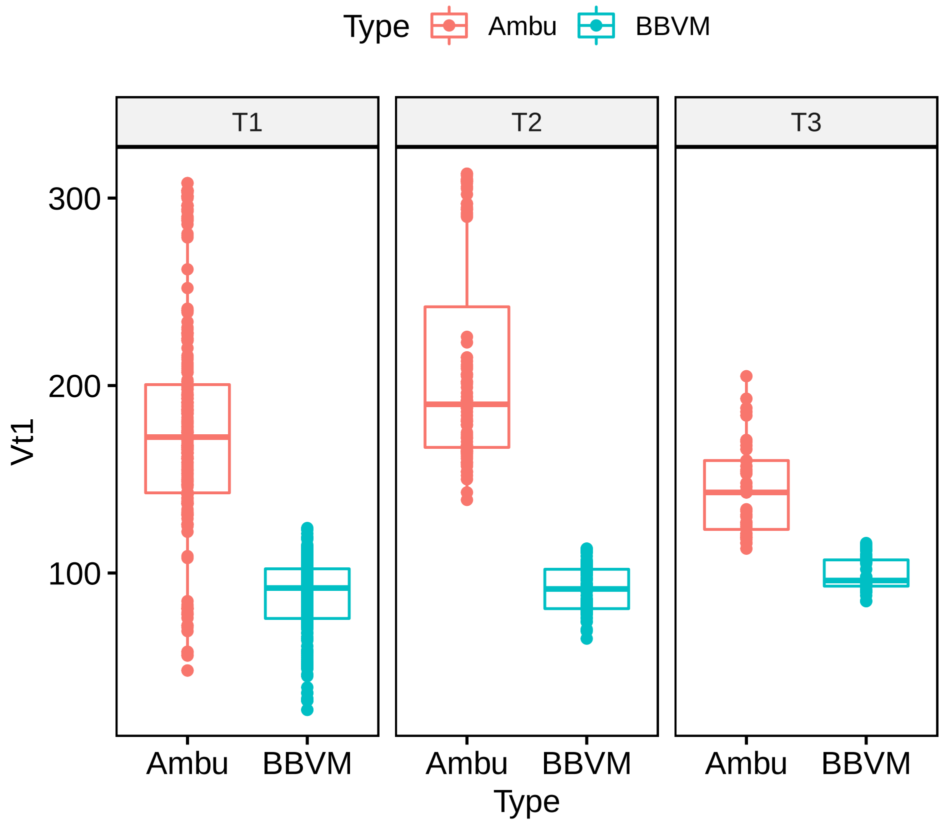

- **Summary statistics of Vt_1 by Participants and Type**

ID	Type	variable	n	min	max	median	iqr	mean	sd	se	ci
A5	Ambu	Vt1	20	125	183	141.0	15.75	144.35	15.517	3.470	7.262
A6	Ambu	Vt1	20	108	202	158.5	41.75	158.80	26.514	5.929	12.409
B1	Ambu	Vt1	20	126	210	159.0	29.75	159.35	21.291	4.761	9.964
B2	Ambu	Vt1	20	133	181	167.5	13.00	165.40	11.537	2.580	5.399
B3	Ambu	Vt1	20	193	252	228.0	11.25	227.45	12.829	2.869	6.004
B4	Ambu	Vt1	20	164	216	190.5	19.00	190.75	12.863	2.876	6.020
B5	Ambu	Vt1	20	262	308	295.0	14.00	292.95	11.362	2.541	5.318
B6	Ambu	Vt1	20	109	171	155.0	22.75	151.80	15.896	3.555	7.440
C1	Ambu	Vt1	20	143	205	160.0	23.00	164.45	18.515	4.140	8.665
C2	Ambu	Vt1	20	139	215	165.0	18.00	168.90	20.825	4.657	9.746
C3	Ambu	Vt1	20	159	226	183.0	25.25	186.25	19.045	4.259	8.913
D1	Ambu	Vt1	20	178	220	195.0	9.50	196.55	9.741	2.178	4.559
D2	Ambu	Vt1	20	290	313	305.5	14.50	302.80	8.134	1.819	3.807
D4	Ambu	Vt1	20	48	85	77.0	14.75	72.30	11.022	2.465	5.158
E1	Ambu	Vt1	20	152	213	188.0	36.25	184.70	20.321	4.544	9.511
E2	Ambu	Vt1	20	113	148	122.5	8.75	124.35	8.132	1.818	3.806
A5	BBVM	Vt1	20	27	65	49.5	18.50	46.80	11.312	2.529	5.294
A6	BBVM	Vt1	20	49	94	68.0	20.50	66.40	13.196	2.951	6.176

ID	Type	variable	n	min	max	median	iqr	mean	sd	se	ci
B1	BBVM	Vt1	20	87	101	97.5	7.25	95.50	4.640	1.037	2.171
B2	BBVM	Vt1	20	100	114	106.0	5.25	106.70	3.962	0.886	1.854
B3	BBVM	Vt1	20	68	96	81.5	11.00	81.50	6.732	1.505	3.151
B4	BBVM	Vt1	20	89	108	96.0	9.00	96.70	5.391	1.205	2.523
B5	BBVM	Vt1	20	68	86	75.0	5.25	76.20	4.336	0.970	2.029
B6	BBVM	Vt1	20	84	106	91.5	9.50	91.90	5.821	1.302	2.724
C1	BBVM	Vt1	20	85	116	107.0	7.75	105.20	9.379	2.097	4.389
C2	BBVM	Vt1	20	94	113	105.0	6.50	105.70	4.824	1.079	2.258
C3	BBVM	Vt1	20	70	93	82.5	5.25	82.20	4.675	1.045	2.188
D1	BBVM	Vt1	20	97	124	112.5	10.00	111.90	7.663	1.714	3.587
D2	BBVM	Vt1	20	65	106	97.0	6.25	95.40	9.304	2.080	4.355
D4	BBVM	Vt1	20	93	110	104.0	9.25	102.70	5.202	1.163	2.435
E1	BBVM	Vt1	20	69	99	80.5	5.50	81.75	6.812	1.523	3.188
E2	BBVM	Vt1	20	90	98	94.0	2.50	93.60	2.326	0.520	1.089

- **Visualizing the Distrubution of Vt_1 by Participants and Type**

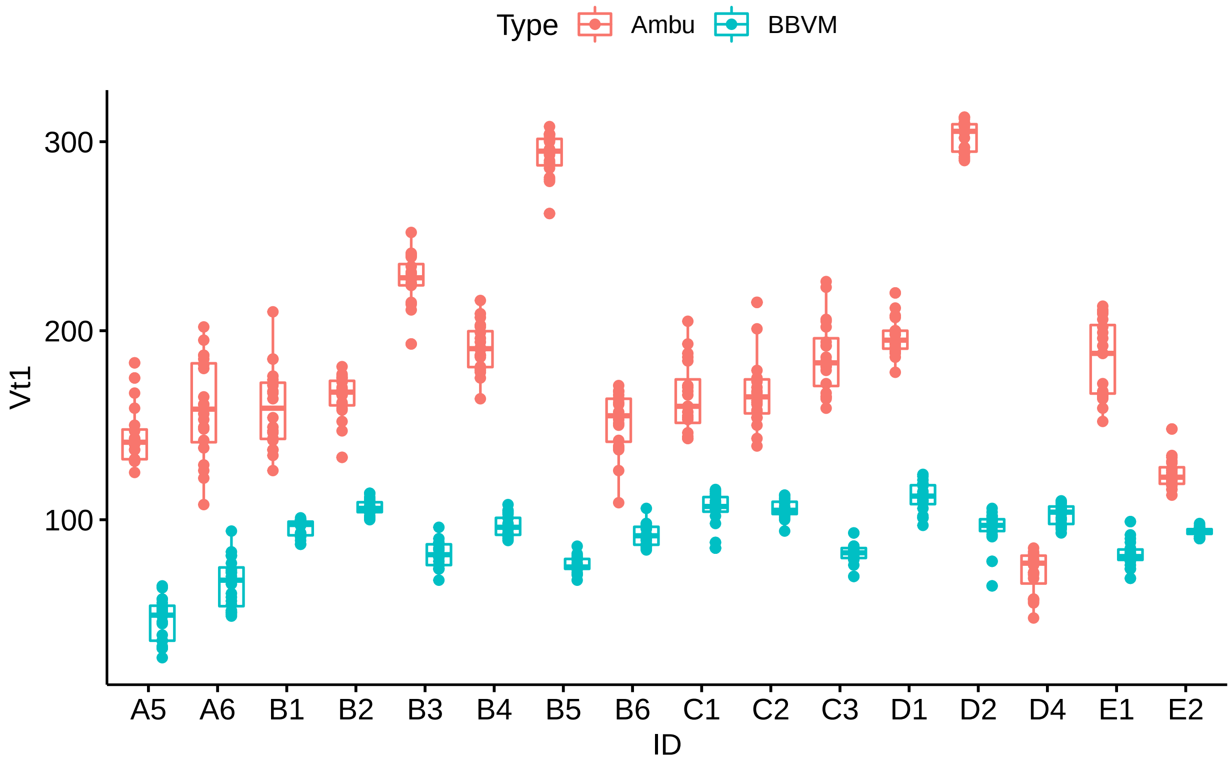

- **Basic test of Normality (Shapiro's Test applied to each Paricipant by Type)**

ID	Type	variable	statistic	p
A5	Ambu	Vt1	0.8631390	0.0089208
A6	Ambu	Vt1	0.9682977	0.7185972

ID	Type	variable	statistic	p
B1	Ambu	Vt1	0.9567313	0.4807907
B2	Ambu	Vt1	0.9071079	0.0561550
B3	Ambu	Vt1	0.9432352	0.2758205
B4	Ambu	Vt1	0.9896954	0.9977690
B5	Ambu	Vt1	0.9172731	0.0878151
B6	Ambu	Vt1	0.9038318	0.0486842
C1	Ambu	Vt1	0.9206178	0.1018360
C2	Ambu	Vt1	0.8908009	0.0278202
C3	Ambu	Vt1	0.9448964	0.2961716
D1	Ambu	Vt1	0.9620689	0.5859614
D2	Ambu	Vt1	0.8831653	0.0201796
D4	Ambu	Vt1	0.8641634	0.0092926
E1	Ambu	Vt1	0.9001991	0.0415948
E2	Ambu	Vt1	0.9085474	0.0598040
A5	BBVM	Vt1	0.9500941	0.3685205
A6	BBVM	Vt1	0.9364393	0.2051986
B1	BBVM	Vt1	0.8709671	0.0122184
B2	BBVM	Vt1	0.9568896	0.4837344
B3	BBVM	Vt1	0.9812010	0.9486449
B4	BBVM	Vt1	0.9453111	0.3014546
B5	BBVM	Vt1	0.9742554	0.8409091
B6	BBVM	Vt1	0.9287809	0.1462922
C1	BBVM	Vt1	0.8380680	0.0033856
C2	BBVM	Vt1	0.9506951	0.3777636
C3	BBVM	Vt1	0.9395395	0.2350188
D1	BBVM	Vt1	0.9641524	0.6296854
D2	BBVM	Vt1	0.7687115	0.0003059
D4	BBVM	Vt1	0.9324632	0.1722071
E1	BBVM	Vt1	0.9589355	0.5228079
E2	BBVM	Vt1	0.9481931	0.3404944

- **Visualizing the differences between the BVM Types per each participant**

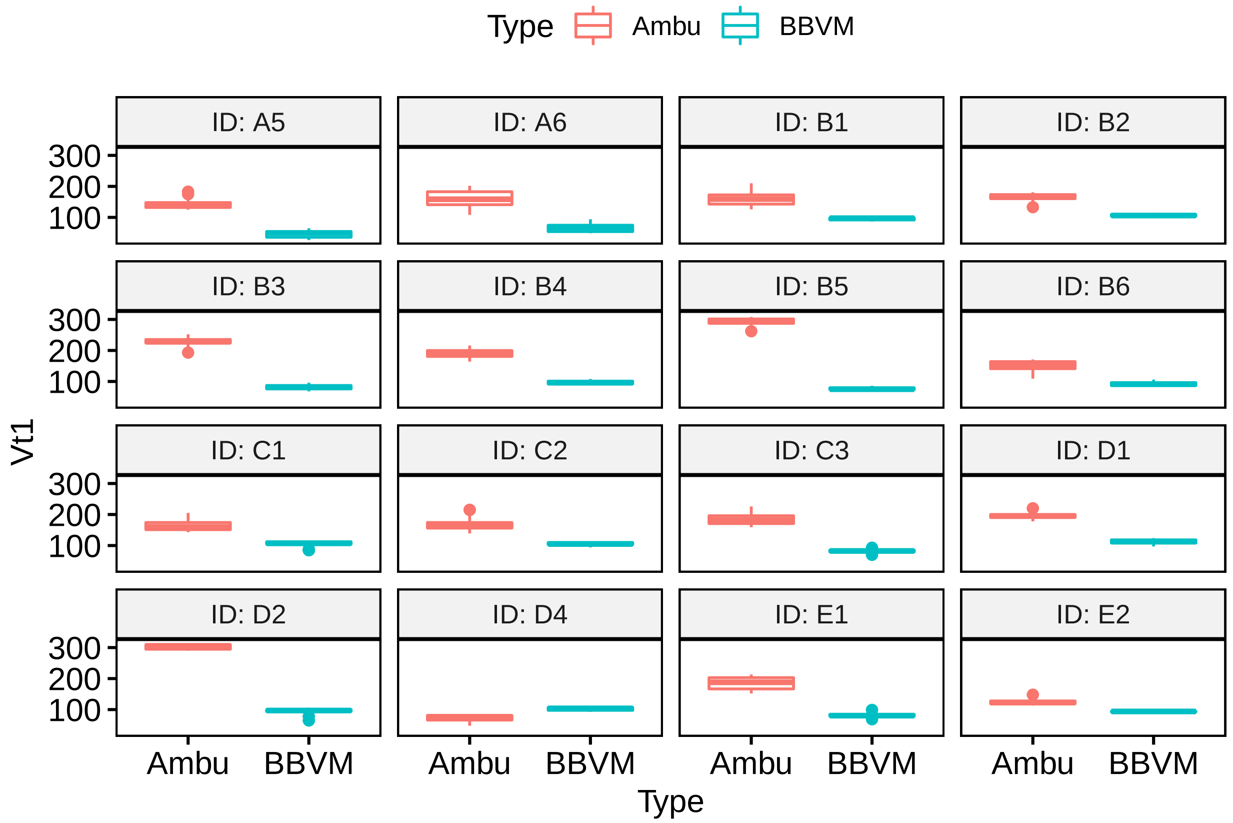

- **Pairwise T-test comparing the BVM Types by each participant**

ID	.y.	group1	group2	n1	n2	statistic	df	p
A5	Vt1	Ambu	BBVM	20	20	28.77597	19	0
A6	Vt1	Ambu	BBVM	20	20	13.74015	19	0
B1	Vt1	Ambu	BBVM	20	20	11.83062	19	0
B2	Vt1	Ambu	BBVM	20	20	18.70537	19	0
B3	Vt1	Ambu	BBVM	20	20	43.01395	19	0
B4	Vt1	Ambu	BBVM	20	20	34.86639	19	0
B5	Vt1	Ambu	BBVM	20	20	83.34997	19	0
B6	Vt1	Ambu	BBVM	20	20	14.22735	19	0
C1	Vt1	Ambu	BBVM	20	20	15.73827	19	0
C2	Vt1	Ambu	BBVM	20	20	13.17721	19	0
C3	Vt1	Ambu	BBVM	20	20	25.46761	19	0
D1	Vt1	Ambu	BBVM	20	20	37.47833	19	0
D2	Vt1	Ambu	BBVM	20	20	63.72772	19	0
D4	Vt1	Ambu	BBVM	20	20	-10.47784	19	0
E1	Vt1	Ambu	BBVM	20	20	23.55814	19	0
E2	Vt1	Ambu	BBVM	20	20	17.21989	19	0

ANOVA approach for the comparisons

- **"Interaction" plot between the Type and the repeated measurements, Trial, on Vt**

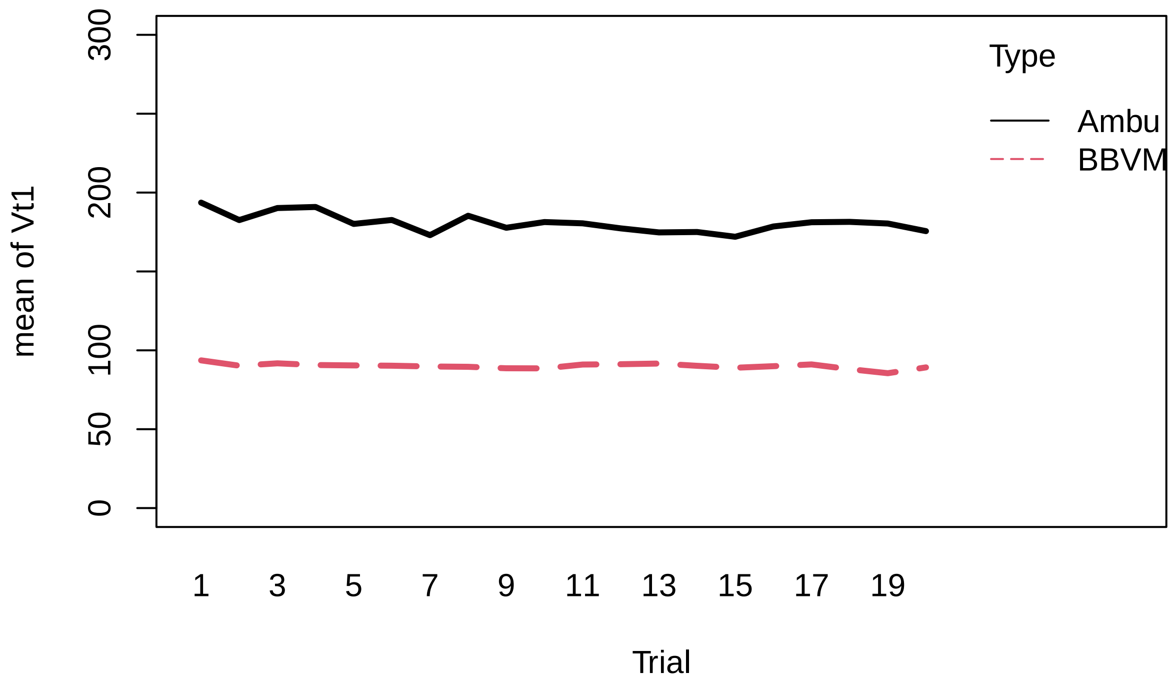

- **With Type only and also accounting for the random effects of the Participants.**

```
##
## Call:
## aov(formula = Vt1 ~ Type + Error(ID), data = data0)
##
## Grand Mean: 135.3531
##
## Stratum 1: ID
##
## Terms:
##                 Residuals
## Sum of Squares   504784.6
## Deg. of Freedom        15
##
## Residual standard error: 183.4457
##
## Stratum 2: Within
##
```

```
## Terms:
##                       Type Residuals
## Sum of Squares  1315875.6  651203.9
## Deg. of Freedom         1       623
##
## Residual standard error: 32.33065
## Estimated effects are balanced
```

```
##           Df Sum Sq Mean Sq F value Pr(>F)
## Residuals 15 504785   33652
```

```
##            Df  Sum Sq Mean Sq F value Pr(>F)
## Type        1 1315876 1315876    1259 <2e-16 ***
## Residuals 623  651204    1045
## ---
## Signif. codes:  0 '***' 0.001 '**' 0.01 '*' 0.05 '.' 0.1 ' ' 1
```

- **With Type and Exp and also accounting for the random effects of the Participants (unbalanced case).**

```
##
## Call:
## aov(formula = Vt1 ~ Type + Exp + Error(ID), data = data0)
##
## Grand Mean: 135.3531
##
## Stratum 1: ID
##
## Terms:
##                       Exp Residuals
## Sum of Squares   58513.2  446271.4
## Deg. of Freedom        2        13
##
## Residual standard error: 185.2797
## Estimated effects may be unbalanced
##
## Stratum 2: Within
##
## Terms:
##                       Type Residuals
## Sum of Squares  1315875.6  651203.9
## Deg. of Freedom         1       623
##
## Residual standard error: 32.33065
## Estimated effects are balanced
```

```
##           Df Sum Sq Mean Sq F value Pr(>F)
## Exp        2  58513   29257   0.852  0.449
## Residuals 13 446271   34329
```

```
##            Df  Sum Sq Mean Sq F value Pr(>F)
## Type        1 1315876 1315876    1259 <2e-16 ***
```

```
## Residuals 623  651204    1045
## ---
## Signif. codes:  0 '***' 0.001 '**' 0.01 '*' 0.05 '.' 0.1 ' ' 1
```

- **With Type and Gender also accounting for the random effects of the Participants (unbalanced case).**

```
##
## Call:
## aov(formula = Vt1 ~ Type + Gender + Error(ID), data = data0)
##
## Grand Mean: 135.3531
##
## Stratum 1: ID
##
## Terms:
##                    Gender Residuals
## Sum of Squares   26940.7  477843.9
## Deg. of Freedom        1        14
##
## Residual standard error: 184.7477
## Estimated effects are balanced
##
## Stratum 2: Within
##
## Terms:
##                      Type Residuals
## Sum of Squares  1315875.6  651203.9
## Deg. of Freedom         1       623
##
## Residual standard error: 32.33065
## Estimated effects are balanced
```

```
##           Df Sum Sq Mean Sq F value Pr(>F)
## Gender     1  26941   26941   0.789  0.389
## Residuals 14 477844   34132
```

```
##            Df  Sum Sq Mean Sq F value Pr(>F)
## Type        1 1315876 1315876    1259 <2e-16 ***
## Residuals 623  651204    1045
## ---
## Signif. codes:  0 '***' 0.001 '**' 0.01 '*' 0.05 '.' 0.1 ' ' 1
```

- **With Type, Exp and Gender also accounting for the random effects of the Participants (unbalanced case).**

```
##
## Call:
## aov(formula = Vt1 ~ Type + Gender + Exp + Error(ID), data = data0)
##
## Grand Mean: 135.3531
##
## Stratum 1: ID
##
## Terms:
##                    Gender      Exp Residuals
## Sum of Squares   26940.7 170729.0  307114.9
## Deg. of Freedom        1        2        12
##
## Residual standard error: 159.9778
## Estimated effects may be unbalanced
##
## Stratum 2: Within
##
## Terms:
##                       Type Residuals
## Sum of Squares  1315875.6  651203.9
## Deg. of Freedom         1       623
##
## Residual standard error: 32.33065
## Estimated effects are balanced
```

```
##           Df Sum Sq Mean Sq F value Pr(>F)
## Gender     1  26941   26941   1.053 0.3251
## Exp        2 170729   85365   3.335 0.0705 .
## Residuals 12 307115   25593
## ---
## Signif. codes:  0 '***' 0.001 '**' 0.01 '*' 0.05 '.' 0.1 ' ' 1
```

```
##            Df  Sum Sq Mean Sq F value Pr(>F)
## Type        1 1315876 1315876    1259 <2e-16 ***
## Residuals 623  651204    1045
## ---
## Signif. codes:  0 '***' 0.001 '**' 0.01 '*' 0.05 '.' 0.1 ' ' 1
```
