# Supplementary material for: Improved simulated ventilation with a novel tidal volume and peak inspiratory pressure controlling bag valve mask: A pilot study
Source: Resusc Plus. 2023 Jan 5;13:100350. doi: 10.1016/j.resplu.2022.100350 (PMC9841173; doi:10.1016/j.resplu.2022.100350)
Supplement: Supplementary data 6 [file mmc6.pdf]

# Comparing Ambu versus BBVM*

The Pediatric Mannequin Trial

Supplement #5, Analysis of $PIP$ under the **Low PIP** Condition

## Summary: Experiment setting # 2, $PIP$ Measurements

### • Setting up the Data Frame ($PIP$ Measurements)

```
## 'data.frame':    320 obs. of  6 variables:
##  $ ID    : Factor w/ 16 levels "A5","A6","B1",..: 1 1 1 1 1 1 1 1 1 1 ...
##  $ Gender: Factor w/ 2 levels "F","M": 1 1 1 1 1 1 1 1 1 1 ...
##  $ Exp   : Factor w/ 3 levels "T1","T2","T3": 1 1 1 1 1 1 1 1 1 1 ...
##  $ Trial : int  1 2 3 4 5 6 7 8 9 10 ...
##  $ Ambu  : num  17 14.7 16.1 15.1 15.1 15.1 14.2 14.2 14.2 14.7 ...
##  $ BBVM  : num  15.1 10.4 8.5 8.5 6.2 10.9 10.4 6.2 10.4 11.8 ...
```

### • The Structure of the Pediatric Data

| ID | Gender | Exp | Trial | Ambu | BBVM |
|---|---|---|---|---|---|
| A5 | F | T1 | 1 | 17.0 | 15.1 |
| A5 | F | T1 | 2 | 14.7 | 10.4 |
| A5 | F | T1 | 3 | 16.1 | 8.5 |
| A5 | F | T1 | 4 | 15.1 | 8.5 |
| A5 | F | T1 | 5 | 15.1 | 6.2 |

### • Changing the data frame from a wide format to a Long Style

```
## 'data.frame':    640 obs. of  6 variables:
##  $ ID    : Factor w/ 16 levels "A5","A6","B1",..: 1 1 1 1 1 1 1 1 1 1 ...
##  $ Gender: Factor w/ 2 levels "F","M": 1 1 1 1 1 1 1 1 1 1 ...
##  $ Exp   : Factor w/ 3 levels "T1","T2","T3": 1 1 1 1 1 1 1 1 1 1 ...
##  $ Trial : int  1 2 3 4 5 6 7 8 9 10 ...
##  $ Type  : Factor w/ 2 levels "Ambu","BBVM": 1 1 1 1 1 1 1 1 1 1 ...
##  $ PIP2  : num  17 14.7 16.1 15.1 15.1 15.1 14.2 14.2 14.2 14.7 ...
```

*Supplemental Report to the *Improved Ventilation with a Novel Tidal Volume and Peak Inspiratory Pressure Controlling Bag Valve Mask–A Pilot Study*

- **The numberof participants per each Gender by Experience group**

| | F | M |
|---|---|---|
| T1 | 6 | 4 |
| T2 | 4 | 0 |
| T3 | 0 | 2 |

- **The sample sizes per each Gender by Experience group**

| Exp | Gender | n | prop |
|---|---|---|---|
| T1 | F | 240 | 60 |
| T1 | M | 160 | 40 |
| T2 | F | 160 | 100 |
| T3 | M | 80 | 100 |

- **Summary statistics for $PIP_2$ by the two BVM types (while ignoring all other factors)**

| Type | variable | n | min | max | median | iqr | mean | sd | se | ci |
|---|---|---|---|---|---|---|---|---|---|---|
| Ambu | PIP2 | 320 | 10.4 | 23.7 | 16.35 | 3.425 | 16.448 | 2.465 | 0.138 | 0.271 |
| BBVM | PIP2 | 320 | 5.2 | 20.3 | 15.40 | 4.300 | 14.589 | 2.992 | 0.167 | 0.329 |

- **Visualizing the Distrubution of $PIP_2$ by the two BVM Types (while ignoring all other factors)**

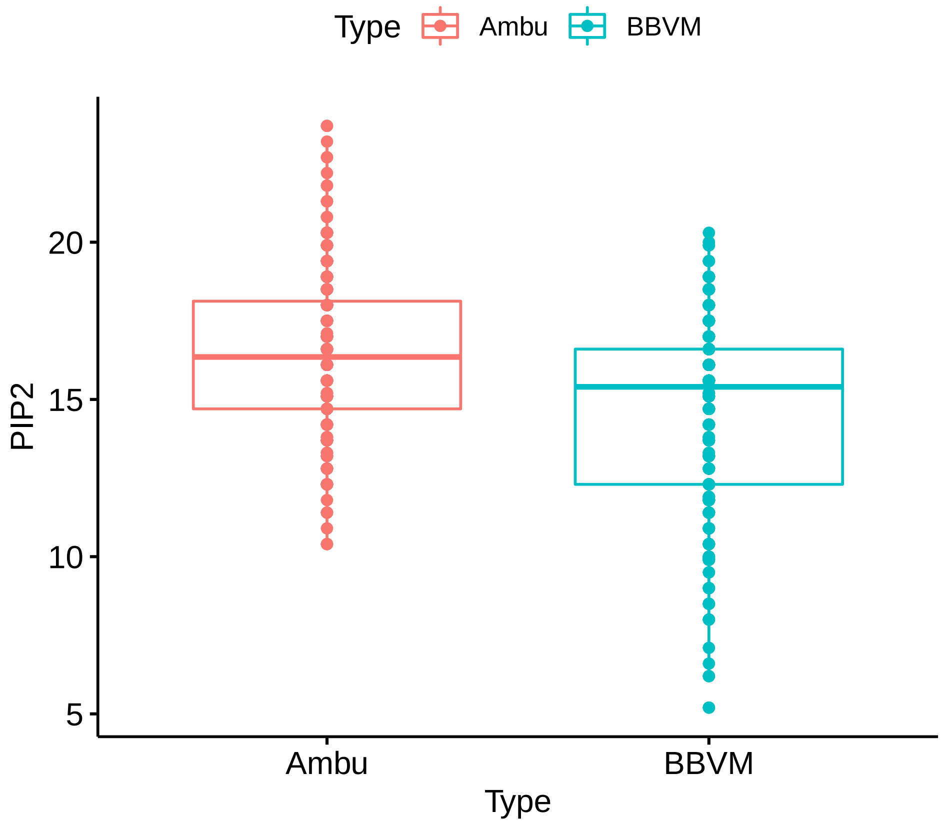


- **Summary statistics for $PIP_2$ by Gender and Type**

| Gender | Type | variable | n | min | max | median | iqr | mean | sd | se | ci |
|---|---|---|---|---|---|---|---|---|---|---|---|
| F | Ambu | PIP2 | 200 | 10.4 | 20.3 | 15.6 | 2.8 | 15.690 | 2.160 | 0.153 | 0.301 |
| M | Ambu | PIP2 | 120 | 12.3 | 23.7 | 17.5 | 3.8 | 17.713 | 2.430 | 0.222 | 0.439 |
| F | BBVM | PIP2 | 200 | 6.2 | 20.3 | 14.2 | 4.8 | 14.051 | 2.902 | 0.205 | 0.405 |
| M | BBVM | PIP2 | 120 | 5.2 | 20.0 | 16.1 | 2.8 | 15.485 | 2.938 | 0.268 | 0.531 |

- **Visualizing the Distrubution of $PIP_2$ by Type for each Gender**

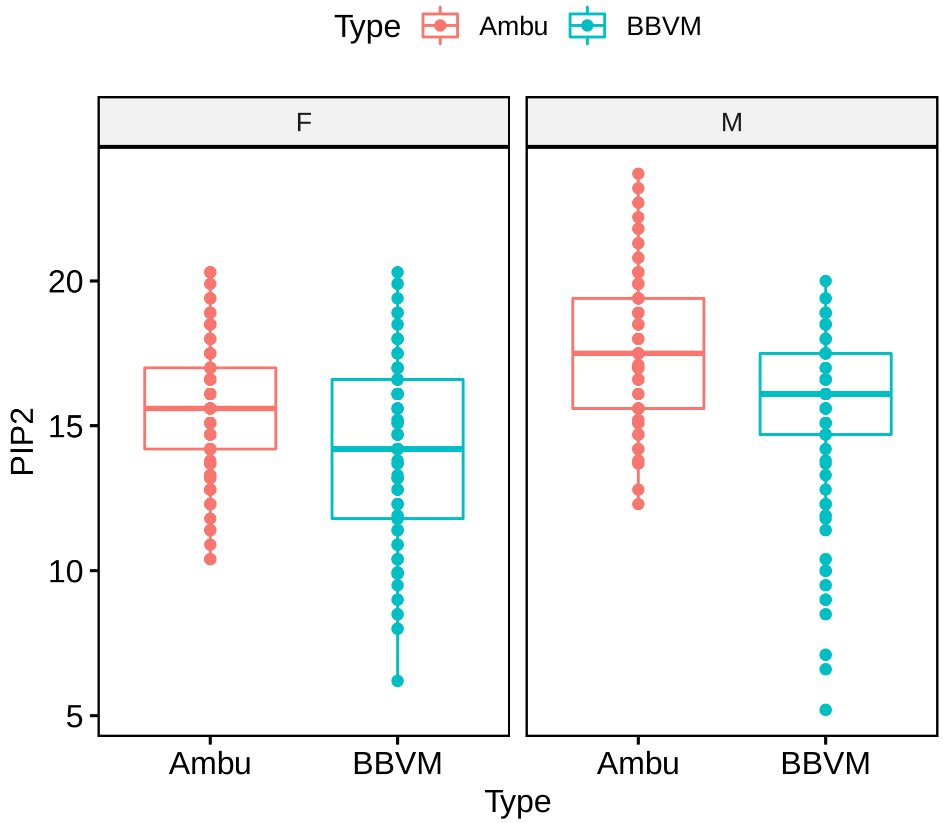


- **Summary statistics for $PIP_2$ by Type and Years of Expereince**

| Exp | Type | variable | n | min | max | median | iqr | mean | sd | se | ci |
|---|---|---|---|---|---|---|---|---|---|---|---|
| T1 | Ambu | PIP2 | 200 | 10.4 | 23.7 | 15.85 | 3.800 | 16.200 | 2.770 | 0.196 | 0.386 |
| T2 | Ambu | PIP2 | 80 | 12.3 | 20.3 | 16.60 | 2.400 | 16.808 | 1.586 | 0.177 | 0.353 |
| T3 | Ambu | PIP2 | 40 | 12.3 | 21.3 | 17.00 | 3.425 | 16.972 | 2.141 | 0.339 | 0.685 |
| T1 | BBVM | PIP2 | 200 | 5.2 | 20.0 | 15.15 | 4.700 | 14.324 | 3.201 | 0.226 | 0.446 |
| T2 | BBVM | PIP2 | 80 | 9.5 | 20.3 | 16.10 | 5.700 | 14.955 | 3.014 | 0.337 | 0.671 |
| T3 | BBVM | PIP2 | 40 | 11.4 | 16.6 | 15.60 | 1.400 | 15.180 | 1.258 | 0.199 | 0.402 |

- **Visualizing the Distrubution of $PIP_2$ by Type and Years of Expereince**

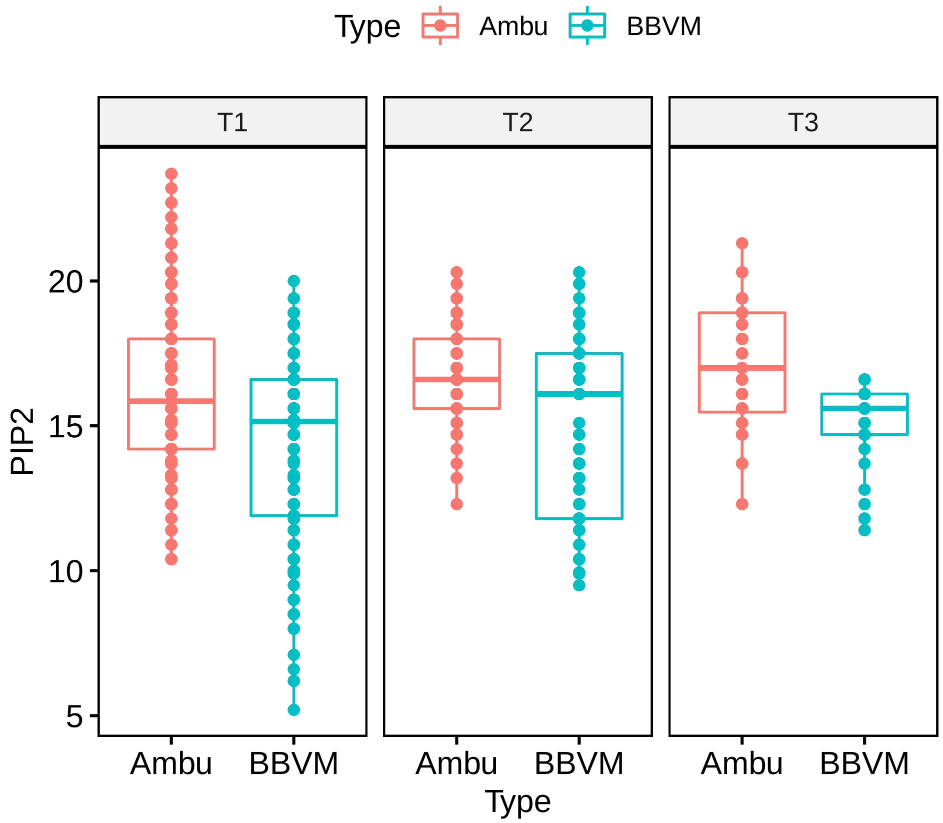


- **Summary statistics of $PIP_2$ by Participants and Type**

| ID | Type | variable | n | min | max | median | iqr | mean | sd | se | ci |
|---|---|---|---|---|---|---|---|---|---|---|---|
| A5 | Ambu | PIP2 | 20 | 12.30 | 17.0 | 14.45 | 1.150 | 14.645 | 1.266 | 0.283 | 0.592 |
| A6 | Ambu | PIP2 | 20 | 11.80 | 16.6 | 14.20 | 1.400 | 14.355 | 1.153 | 0.258 | 0.540 |
| B1 | Ambu | PIP2 | 20 | 14.70 | 20.3 | 17.50 | 1.125 | 17.670 | 1.381 | 0.309 | 0.646 |
| B2 | Ambu | PIP2 | 20 | 17.00 | 22.7 | 20.10 | 1.525 | 20.220 | 1.333 | 0.298 | 0.624 |
| B3 | Ambu | PIP2 | 20 | 13.70 | 18.9 | 16.80 | 2.025 | 16.695 | 1.399 | 0.313 | 0.655 |
| B4 | Ambu | PIP2 | 20 | 17.00 | 23.7 | 19.65 | 1.400 | 19.920 | 1.706 | 0.381 | 0.798 |
| B5 | Ambu | PIP2 | 20 | 12.80 | 18.0 | 15.20 | 2.025 | 15.360 | 1.382 | 0.309 | 0.647 |
| B6 | Ambu | PIP2 | 20 | 12.30 | 16.1 | 13.55 | 1.400 | 13.715 | 1.169 | 0.262 | 0.547 |
| C1 | Ambu | PIP2 | 20 | 12.30 | 19.4 | 15.35 | 1.525 | 15.425 | 1.579 | 0.353 | 0.739 |
| C2 | Ambu | PIP2 | 20 | 14.70 | 19.4 | 16.60 | 2.025 | 16.995 | 1.347 | 0.301 | 0.630 |
| C3 | Ambu | PIP2 | 20 | 15.10 | 20.3 | 18.50 | 1.400 | 18.170 | 1.436 | 0.321 | 0.672 |
| D1 | Ambu | PIP2 | 20 | 15.10 | 19.4 | 17.00 | 1.900 | 16.835 | 1.197 | 0.268 | 0.560 |
| D2 | Ambu | PIP2 | 20 | 14.70 | 18.5 | 16.35 | 0.900 | 16.500 | 0.968 | 0.217 | 0.453 |
| D4 | Ambu | PIP2 | 20 | 10.40 | 16.1 | 12.55 | 2.300 | 12.585 | 1.581 | 0.353 | 0.740 |
| E1 | Ambu | PIP2 | 20 | 12.30 | 17.5 | 15.60 | 1.600 | 15.565 | 1.390 | 0.311 | 0.650 |
| E2 | Ambu | PIP2 | 20 | 15.60 | 21.3 | 18.70 | 1.525 | 18.520 | 1.370 | 0.306 | 0.641 |
| A5 | BBVM | PIP2 | 20 | 6.20 | 15.1 | 9.90 | 2.025 | 9.595 | 2.029 | 0.454 | 0.949 |
| A6 | BBVM | PIP2 | 20 | 9.00 | 16.1 | 12.30 | 1.925 | 12.340 | 1.657 | 0.371 | 0.776 |

| ID | Type | variable | n | min | max | median | iqr | mean | sd | se | ci |
|---|---|---|---|---|---|---|---|---|---|---|---|
| B1 | BBVM | PIP2 | 20 | 15.60 | 18.0 | 16.10 | 0.500 | 16.385 | 0.607 | 0.136 | 0.284 |
| B2 | BBVM | PIP2 | 20 | 16.60 | 20.0 | 17.75 | 1.000 | 17.925 | 0.833 | 0.186 | 0.390 |
| B3 | BBVM | PIP2 | 20 | 14.70 | 17.0 | 15.85 | 1.500 | 15.825 | 0.753 | 0.168 | 0.352 |
| B4 | BBVM | PIP2 | 20 | 15.60 | 16.6 | 16.10 | 0.500 | 16.150 | 0.359 | 0.080 | 0.168 |
| B5 | BBVM | PIP2 | 20 | 5.20 | 13.8 | 10.00 | 3.000 | 10.225 | 2.260 | 0.505 | 1.058 |
| B6 | BBVM | PIP2 | 20 | 11.90 | 15.6 | 14.20 | 1.100 | 14.300 | 1.031 | 0.230 | 0.482 |
| C1 | BBVM | PIP2 | 20 | 11.40 | 16.6 | 15.10 | 1.650 | 14.745 | 1.569 | 0.351 | 0.734 |
| C2 | BBVM | PIP2 | 20 | 17.50 | 20.3 | 18.00 | 1.025 | 18.455 | 0.891 | 0.199 | 0.417 |
| C3 | BBVM | PIP2 | 20 | 9.50 | 17.0 | 12.30 | 1.900 | 12.765 | 1.795 | 0.401 | 0.840 |
| D1 | BBVM | PIP2 | 20 | 16.10 | 19.4 | 18.50 | 0.900 | 18.250 | 0.882 | 0.197 | 0.413 |
| D2 | BBVM | PIP2 | 20 | 9.94 | 14.7 | 11.80 | 2.125 | 11.844 | 1.373 | 0.307 | 0.643 |
| D4 | BBVM | PIP2 | 20 | 9.94 | 14.7 | 12.55 | 1.500 | 12.249 | 1.260 | 0.282 | 0.590 |
| E1 | BBVM | PIP2 | 20 | 16.10 | 18.5 | 16.60 | 0.525 | 16.755 | 0.576 | 0.129 | 0.270 |
| E2 | BBVM | PIP2 | 20 | 14.20 | 16.6 | 15.60 | 0.625 | 15.615 | 0.621 | 0.139 | 0.291 |

- **Visualizing the Distrubution of $PIP_2$ by Participants and Type**

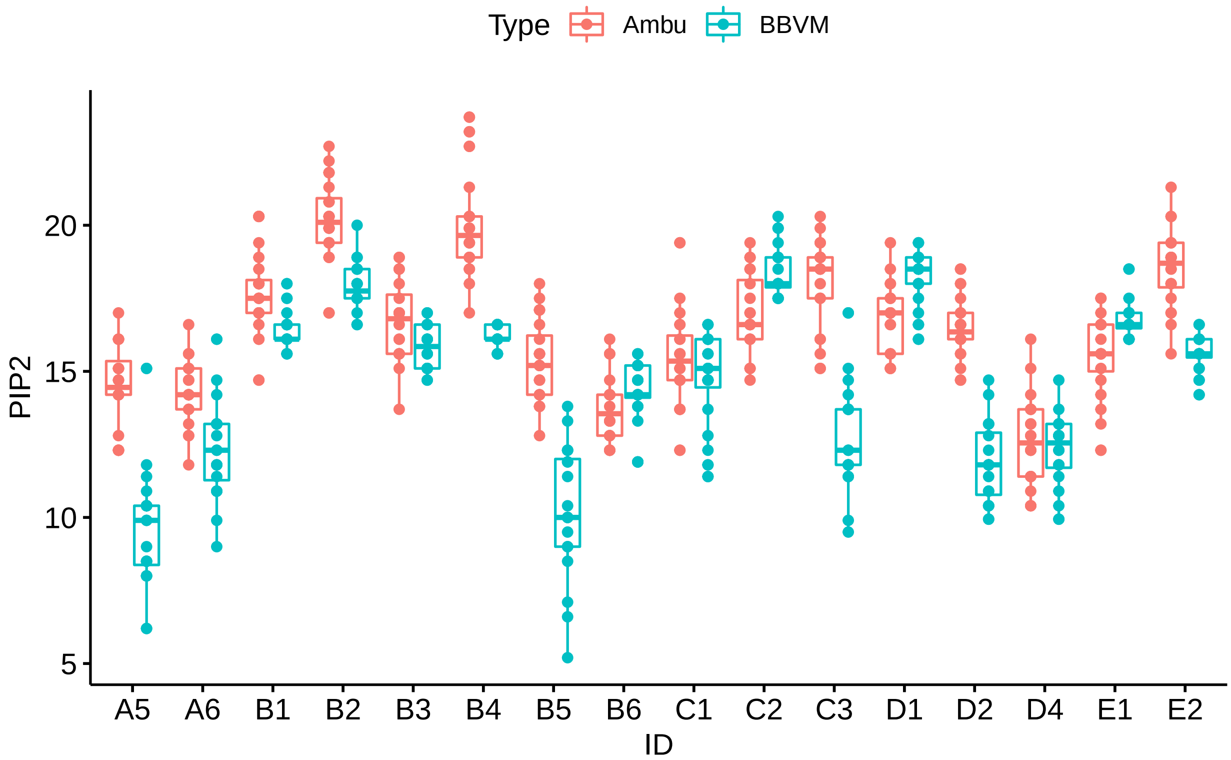


- **Basic test of Normality (Shapiro's Test applied to each Paricipant by Type)**

| ID | Type | variable | statistic | p |
|---|---|---|---|---|
| A5 | Ambu | PIP2 | 0.9270294 | 0.1353545 |
| A6 | Ambu | PIP2 | 0.9735969 | 0.8282563 |

| ID | Type | variable | statistic | p |
|---|---|---|---|---|
| B1 | Ambu | PIP2 | 0.9622562 | 0.5898433 |
| B2 | Ambu | PIP2 | 0.9626647 | 0.5983473 |
| B3 | Ambu | PIP2 | 0.9481458 | 0.3398195 |
| B4 | Ambu | PIP2 | 0.9195949 | 0.0973224 |
| B5 | Ambu | PIP2 | 0.9698076 | 0.7508052 |
| B6 | Ambu | PIP2 | 0.9159244 | 0.0827327 |
| C1 | Ambu | PIP2 | 0.9687033 | 0.7272874 |
| C2 | Ambu | PIP2 | 0.9472773 | 0.3276439 |
| C3 | Ambu | PIP2 | 0.8992832 | 0.0399831 |
| D1 | Ambu | PIP2 | 0.9415271 | 0.2562178 |
| D2 | Ambu | PIP2 | 0.9561468 | 0.4700229 |
| D4 | Ambu | PIP2 | 0.9553162 | 0.4550064 |
| E1 | Ambu | PIP2 | 0.9317541 | 0.1668900 |
| E2 | Ambu | PIP2 | 0.9822567 | 0.9597969 |
| A5 | BBVM | PIP2 | 0.9314691 | 0.1647987 |
| A6 | BBVM | PIP2 | 0.9773774 | 0.8959362 |
| B1 | BBVM | PIP2 | 0.8695546 | 0.0115393 |
| B2 | BBVM | PIP2 | 0.9354908 | 0.1968167 |
| B3 | BBVM | PIP2 | 0.9167738 | 0.0858977 |
| B4 | BBVM | PIP2 | 0.8116810 | 0.0012971 |
| B5 | BBVM | PIP2 | 0.9600144 | 0.5441366 |
| B6 | BBVM | PIP2 | 0.8726814 | 0.0131000 |
| C1 | BBVM | PIP2 | 0.8833489 | 0.0203347 |
| C2 | BBVM | PIP2 | 0.8792052 | 0.0171201 |
| C3 | BBVM | PIP2 | 0.9513334 | 0.3877855 |
| D1 | BBVM | PIP2 | 0.8955271 | 0.0340251 |
| D2 | BBVM | PIP2 | 0.9449353 | 0.2966637 |
| D4 | BBVM | PIP2 | 0.9482841 | 0.3417934 |
| E1 | BBVM | PIP2 | 0.8350212 | 0.0030213 |
| E2 | BBVM | PIP2 | 0.9226563 | 0.1114733 |

- **Visualizing the differences between the BVM Types per each participant**

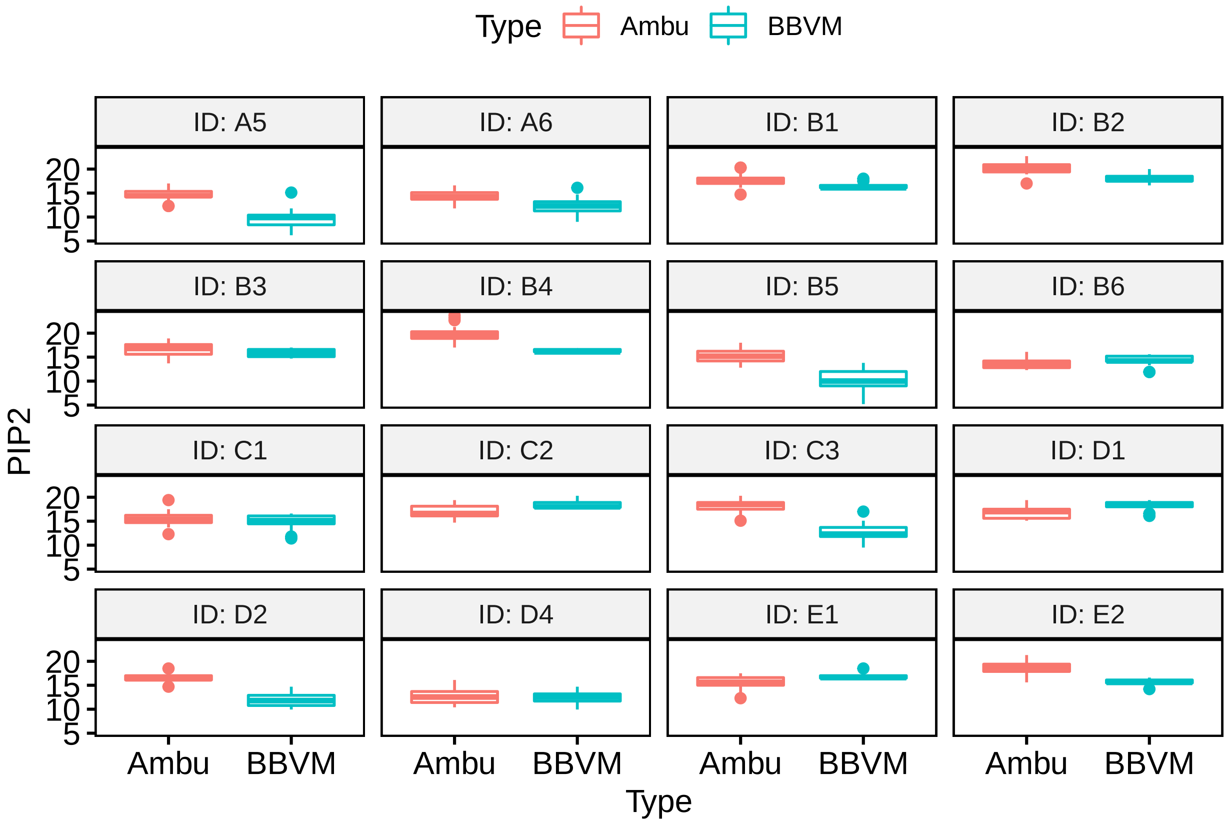


- **Pairwise T-test comparing the BVM Types by each participant**

| ID | .y. | group1 | group2 | n1 | n2 | statistic | df | p |
|---|---|---|---|---|---|---|---|---|
| A5 | PIP2 | Ambu | BBVM | 20 | 20 | 10.5065626 | 19 | 0.00e+00 |
| A6 | PIP2 | Ambu | BBVM | 20 | 20 | 4.2325390 | 19 | 4.51e-04 |
| B1 | PIP2 | Ambu | BBVM | 20 | 20 | 3.5268234 | 19 | 2.25e-03 |
| B2 | PIP2 | Ambu | BBVM | 20 | 20 | 5.5232045 | 19 | 2.50e-05 |
| B3 | PIP2 | Ambu | BBVM | 20 | 20 | 2.4864622 | 19 | 2.24e-02 |
| B4 | PIP2 | Ambu | BBVM | 20 | 20 | 9.2529544 | 19 | 0.00e+00 |
| B5 | PIP2 | Ambu | BBVM | 20 | 20 | 7.2160733 | 19 | 7.00e-07 |
| B6 | PIP2 | Ambu | BBVM | 20 | 20 | -1.6831209 | 19 | 1.09e-01 |
| C1 | PIP2 | Ambu | BBVM | 20 | 20 | 1.3310862 | 19 | 1.99e-01 |
| C2 | PIP2 | Ambu | BBVM | 20 | 20 | -3.4877606 | 19 | 2.46e-03 |
| C3 | PIP2 | Ambu | BBVM | 20 | 20 | 11.7382178 | 19 | 0.00e+00 |
| D1 | PIP2 | Ambu | BBVM | 20 | 20 | -4.6700955 | 19 | 1.67e-04 |
| D2 | PIP2 | Ambu | BBVM | 20 | 20 | 15.0634573 | 19 | 0.00e+00 |
| D4 | PIP2 | Ambu | BBVM | 20 | 20 | 0.7563046 | 19 | 4.59e-01 |
| E1 | PIP2 | Ambu | BBVM | 20 | 20 | -3.7681711 | 19 | 1.30e-03 |
| E2 | PIP2 | Ambu | BBVM | 20 | 20 | 8.2840434 | 19 | 1.00e-07 |

## ANOVA approach for the comparisons

- **“Interaction” plot between the Type and the repeated measurements, Trial, on $Vt$**

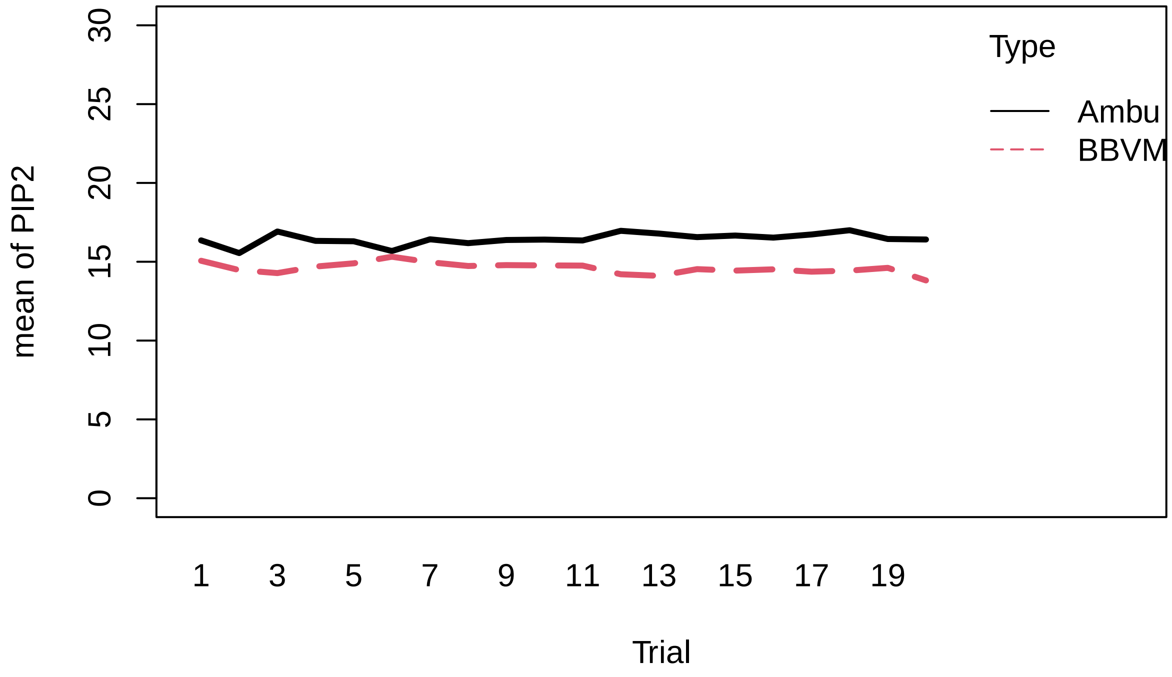


- **With Type only and also accounting for the random effects of the Participants.**

```
##
## Call:
## aov(formula = PIP2 ~ Type + Error(ID), data = data0)
##
## Grand Mean: 15.51869
##
## Stratum 1: ID
##
## Terms:
##                  Residuals
## Sum of Squares   2846.674
## Deg. of Freedom        15
##
## Residual standard error: 13.776
##
## Stratum 2: Within
##
```

```
## Terms:
##                      Type Residuals
## Sum of Squares   553.2384 1947.8180
## Deg. of Freedom         1       623
##
## Residual standard error: 1.768195
## Estimated effects are balanced
```

```
##           Df Sum Sq Mean Sq F value Pr(>F)
## Residuals 15   2847   189.8
```

```
##            Df Sum Sq Mean Sq F value Pr(>F)
## Type        1  553.2   553.2     177 <2e-16 ***
## Residuals 623 1947.8     3.1
## ---
## Signif. codes:  0 '***' 0.001 '**' 0.01 '*' 0.05 '.' 0.1 ' ' 1
```

- **With Type and Exp and also accounting for the random effects of the Participants (unbalanced case).**

```
##
## Call:
## aov(formula = PIP2 ~ Type + Exp + Error(ID), data = data0)
##
## Grand Mean: 15.51869
##
## Stratum 1: ID
##
## Terms:
##                       Exp Residuals
## Sum of Squares    72.2022 2774.4723
## Deg. of Freedom         2        13
##
## Residual standard error: 14.60893
## Estimated effects may be unbalanced
##
## Stratum 2: Within
##
## Terms:
##                      Type Residuals
## Sum of Squares   553.2384 1947.8180
## Deg. of Freedom         1       623
##
## Residual standard error: 1.768195
## Estimated effects are balanced
```

```
##           Df Sum Sq Mean Sq F value Pr(>F)
## Exp        2   72.2    36.1   0.169  0.846
## Residuals 13 2774.5   213.4
```

```
##            Df Sum Sq Mean Sq F value Pr(>F)
## Type        1  553.2   553.2     177 <2e-16 ***
```

```
## Residuals 623 1947.8     3.1
## ---
## Signif. codes:  0 '***' 0.001 '**' 0.01 '*' 0.05 '.' 0.1 ' ' 1
```

- **With Type and Gender also accounting for the random effects of the Participants (unbalanced case).**

```
##
## Call:
## aov(formula = PIP2 ~ Type + Gender + Error(ID), data = data0)
##
## Grand Mean: 15.51869
##
## Stratum 1: ID
##
## Terms:
##                    Gender Residuals
## Sum of Squares   448.2951 2398.3793
## Deg. of Freedom         1        14
##
## Residual standard error: 13.08865
## Estimated effects are balanced
##
## Stratum 2: Within
##
## Terms:
##                      Type Residuals
## Sum of Squares   553.2384 1947.8180
## Deg. of Freedom         1       623
##
## Residual standard error: 1.768195
## Estimated effects are balanced
```

```
##           Df Sum Sq Mean Sq F value Pr(>F)
## Gender     1  448.3   448.3   2.617  0.128
## Residuals 14 2398.4   171.3
```

```
##            Df Sum Sq Mean Sq F value Pr(>F)
## Type        1  553.2   553.2     177 <2e-16 ***
## Residuals 623 1947.8     3.1
## ---
## Signif. codes:  0 '***' 0.001 '**' 0.01 '*' 0.05 '.' 0.1 ' ' 1
```

- **With Type, Exp and Gender also accounting for the random effects of the Participants (unbalanced case).**

```
##
## Call:
## aov(formula = PIP2 ~ Type + Gender + Exp + Error(ID), data = data0)
##
## Grand Mean: 15.51869
##
## Stratum 1: ID
##
## Terms:
##                    Gender       Exp Residuals
## Sum of Squares   448.2951  305.2304 2093.1489
## Deg. of Freedom         1         2        12
##
## Residual standard error: 13.20716
## Estimated effects may be unbalanced
##
## Stratum 2: Within
##
## Terms:
##                      Type Residuals
## Sum of Squares   553.2384 1947.8180
## Deg. of Freedom         1       623
##
## Residual standard error: 1.768195
## Estimated effects are balanced
```

```
##           Df Sum Sq Mean Sq F value Pr(>F)
## Gender     1  448.3   448.3   2.570  0.135
## Exp        2  305.2   152.6   0.875  0.442
## Residuals 12 2093.1   174.4
```

```
##            Df Sum Sq Mean Sq F value Pr(>F)
## Type        1  553.2   553.2     177 <2e-16 ***
## Residuals 623 1947.8     3.1
## ---
## Signif. codes:  0 '***' 0.001 '**' 0.01 '*' 0.05 '.' 0.1 ' ' 1
```
