# Supplementary material for: Improved simulated ventilation with a novel tidal volume and peak inspiratory pressure controlling bag valve mask: A pilot study
Source: Resusc Plus. 2023 Jan 5;13:100350. doi: 10.1016/j.resplu.2022.100350 (PMC9841173; doi:10.1016/j.resplu.2022.100350)
Supplement: Supplementary data 7 [file mmc7.pdf]

Comparing Ambu versus BBVM*

The Pediatric Mannequin Trial

Supplement #6, Analysis of Vt under the **Low PIP** Condition

Summary: Experiment setting # 2, Vt Measurements

• Setting up the Data Frame (Vt Measurements)

```
## 'data.frame':    320 obs. of  6 variables:
##  $ ID    : Factor w/ 16 levels "A5","A6","B1",..: 1 1 1 1 1 1 1 1 1 1 ...
##  $ Gender: Factor w/ 2 levels "F","M": 1 1 1 1 1 1 1 1 1 1 ...
##  $ Exp   : Factor w/ 3 levels "T1","T2","T3": 1 1 1 1 1 1 1 1 1 1 ...
##  $ Trial : int  1 2 3 4 5 6 7 8 9 10 ...
##  $ Ambu  : int  153 141 142 147 140 145 139 137 132 142 ...
##  $ BBVM  : int  46 62 53 47 48 38 54 59 44 55 ...
```

• The Structure of the Pediatric Data

ID	Gender	Exp	Trial	Ambu	BBVM
A5	F	T1	1	153	46
A5	F	T1	2	141	62
A5	F	T1	3	142	53
A5	F	T1	4	147	47
A5	F	T1	5	140	48

• Changing the data frame from a wide format to a Long Style

```
## 'data.frame':    640 obs. of  6 variables:
##  $ ID    : Factor w/ 16 levels "A5","A6","B1",..: 1 1 1 1 1 1 1 1 1 1 ...
##  $ Gender: Factor w/ 2 levels "F","M": 1 1 1 1 1 1 1 1 1 1 ...
##  $ Exp   : Factor w/ 3 levels "T1","T2","T3": 1 1 1 1 1 1 1 1 1 1 ...
##  $ Trial : int  1 2 3 4 5 6 7 8 9 10 ...
##  $ Type  : Factor w/ 2 levels "Ambu","BBVM": 1 1 1 1 1 1 1 1 1 1 ...
##  $ Vt2   : int  153 141 142 147 140 145 139 137 132 142 ...
```

*Supplemental Report to the *Improved Ventilation with a Novel Tidal Volume and Peak Inspiratory Pressure Controlling Bag Valve Mask–A Pilot Study*

- **The numberof participants per each Gender by Experience group**

	F	M
T1	6	4
T2	4	0
T3	0	2

- **The sample sizes per each Gender by Experience group**

Exp	Gender	n	prop
T1	F	240	60
T1	M	160	40
T2	F	160	100
T3	M	80	100

- **Summary statistics for Vt_2 by the two BVM types (while ignoring all other factors)**

Type	variable	n	min	max	median	iqr	mean	sd	se	ci
Ambu	Vt2	320	41	233	134	51	136.009	41.021	2.293	4.512
BBVM	Vt2	320	30	115	70	21	71.184	15.906	0.889	1.749

- **Visualizing the Distrubution of Vt_2 by the two BVM Types (while ignoring all other factors)**

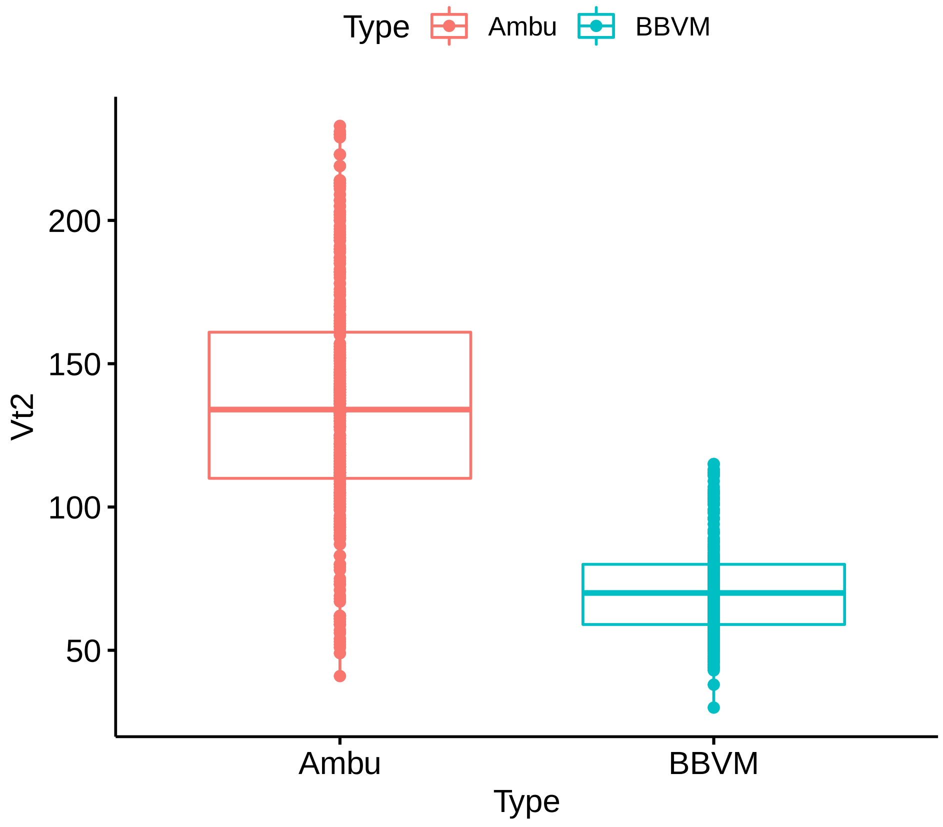

- **Summary statistics for Vt_2 by Gender and Type**

Gender	Type	variable	n	min	max	median	iqr	mean	sd	se	ci
F	Ambu	Vt2	200	41	233	121.5	41.00	132.095	39.515	2.794	5.510
M	Ambu	Vt2	120	49	219	145.5	34.00	142.533	42.790	3.906	7.735
F	BBVM	Vt2	200	38	115	68.5	24.25	72.225	18.096	1.280	2.523
M	BBVM	Vt2	120	30	104	71.5	16.25	69.450	11.214	1.024	2.027

- **Visualizing the Distrubution of Vt_2 by Type for each Gender**

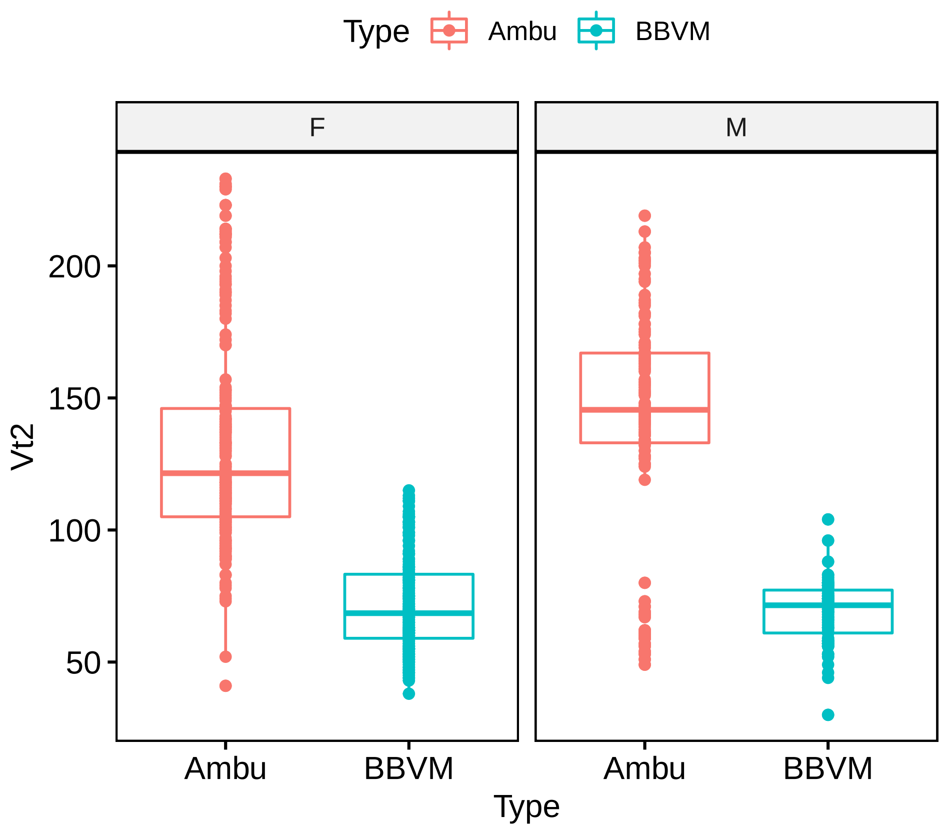

- **Summary statistics for Vt_2 by Type and Years of Expereince**

Exp	Type	variable	n	min	max	median	iqr	mean	sd	se	ci
T1	Ambu	Vt2	200	78	219	139.0	50.00	142.915	33.053	2.337	4.609
T2	Ambu	Vt2	80	41	233	122.5	61.50	136.387	49.713	5.558	11.063
T3	Ambu	Vt2	40	49	164	99.5	78.50	100.725	40.762	6.445	13.036
T1	BBVM	Vt2	200	30	112	72.0	18.00	71.580	15.333	1.084	2.138
T2	BBVM	Vt2	80	48	115	63.0	17.75	71.412	19.089	2.134	4.248
T3	BBVM	Vt2	40	53	83	68.0	22.25	68.750	11.172	1.766	3.573

- **Visualizing the Distrubution of Vt_2 by Type and Years of Expereince**

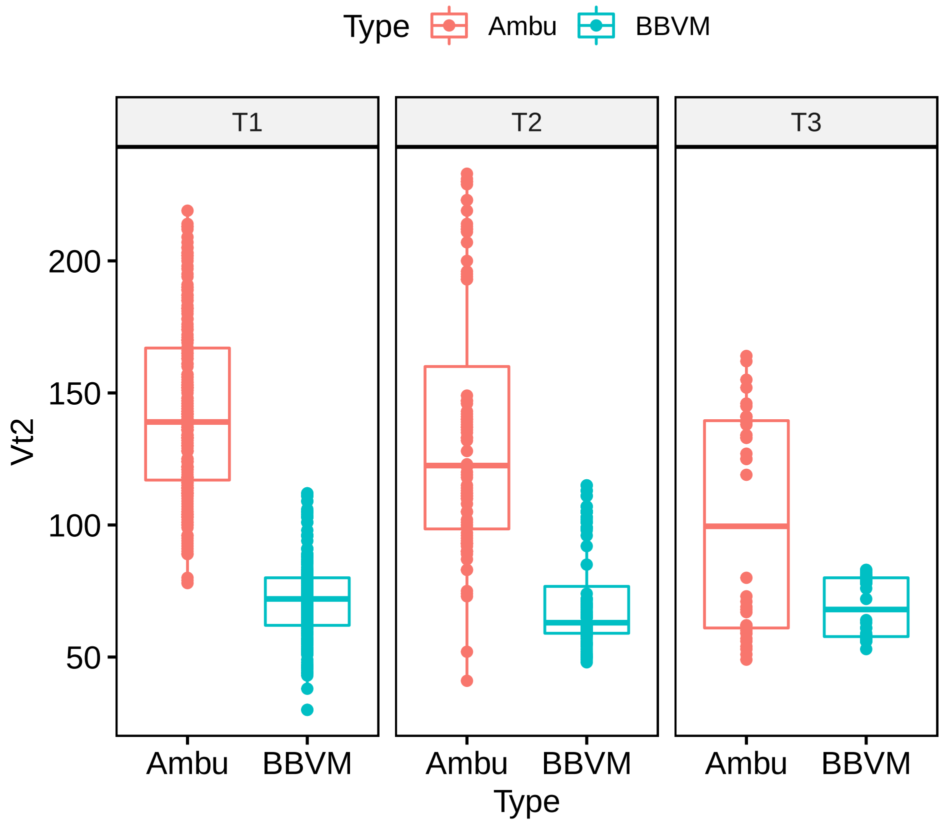

- **Summary statistics of Vt_2 by Participants and Type**

ID	Type	variable	n	min	max	median	iqr	mean	sd	se	ci
A5	Ambu	Vt2	20	122	153	139.5	12.75	139.15	9.527	2.130	4.459
A6	Ambu	Vt2	20	154	214	186.0	19.25	185.50	16.643	3.722	7.789
B1	Ambu	Vt2	20	104	146	122.5	20.25	124.35	13.076	2.924	6.120
B2	Ambu	Vt2	20	132	165	145.5	16.00	146.85	9.928	2.220	4.646
B3	Ambu	Vt2	20	78	122	105.0	16.25	106.00	12.557	2.808	5.877
B4	Ambu	Vt2	20	124	163	140.0	12.75	140.55	10.817	2.419	5.062
B5	Ambu	Vt2	20	174	219	200.5	16.50	197.95	11.500	2.572	5.382
B6	Ambu	Vt2	20	79	134	109.0	20.75	107.75	14.502	3.243	6.787
C1	Ambu	Vt2	20	119	164	140.0	12.25	139.75	11.982	2.679	5.608
C2	Ambu	Vt2	20	90	147	111.5	23.50	114.10	17.903	4.003	8.379
C3	Ambu	Vt2	20	110	149	136.5	17.25	131.95	12.037	2.692	5.634
D1	Ambu	Vt2	20	153	182	167.0	10.50	168.40	8.312	1.859	3.890
D2	Ambu	Vt2	20	193	233	212.5	25.50	212.90	14.104	3.154	6.601
D4	Ambu	Vt2	20	89	151	112.5	15.25	112.65	15.421	3.448	7.217
E1	Ambu	Vt2	20	41	112	90.0	16.00	86.60	17.215	3.849	8.057
E2	Ambu	Vt2	20	49	80	61.0	10.50	61.70	7.794	1.743	3.648
A5	BBVM	Vt2	20	38	62	49.5	7.25	49.80	5.727	1.281	2.680
A6	BBVM	Vt2	20	43	71	58.0	7.25	58.30	6.292	1.407	2.945

ID	Type	variable	n	min	max	median	iqr	mean	sd	se	ci
B1	BBVM	Vt2	20	59	75	68.0	4.00	68.20	4.595	1.028	2.151
B2	BBVM	Vt2	20	59	80	69.0	5.75	69.85	5.344	1.195	2.501
B3	BBVM	Vt2	20	68	83	77.5	7.00	77.00	4.377	0.979	2.048
B4	BBVM	Vt2	20	67	83	74.0	4.75	74.75	3.932	0.879	1.840
B5	BBVM	Vt2	20	46	75	62.5	16.00	62.10	9.233	2.065	4.321
B6	BBVM	Vt2	20	75	91	83.5	6.50	83.05	4.501	1.006	2.107
C1	BBVM	Vt2	20	72	83	80.0	2.25	79.50	2.503	0.560	1.171
C2	BBVM	Vt2	20	58	74	69.0	5.00	67.65	4.209	0.941	1.970
C3	BBVM	Vt2	20	48	63	55.0	7.50	54.55	4.419	0.988	2.068
D1	BBVM	Vt2	20	30	104	73.0	11.75	72.50	17.455	3.903	8.169
D2	BBVM	Vt2	20	85	115	102.5	6.00	102.20	6.895	1.542	3.227
D4	BBVM	Vt2	20	86	112	102.0	9.50	100.25	7.383	1.651	3.455
E1	BBVM	Vt2	20	54	67	61.0	3.25	61.25	3.093	0.692	1.448
E2	BBVM	Vt2	20	53	64	57.5	1.50	58.00	2.575	0.576	1.205

- **Visualizing the Distrubution of Vt_2 by Participants and Type**

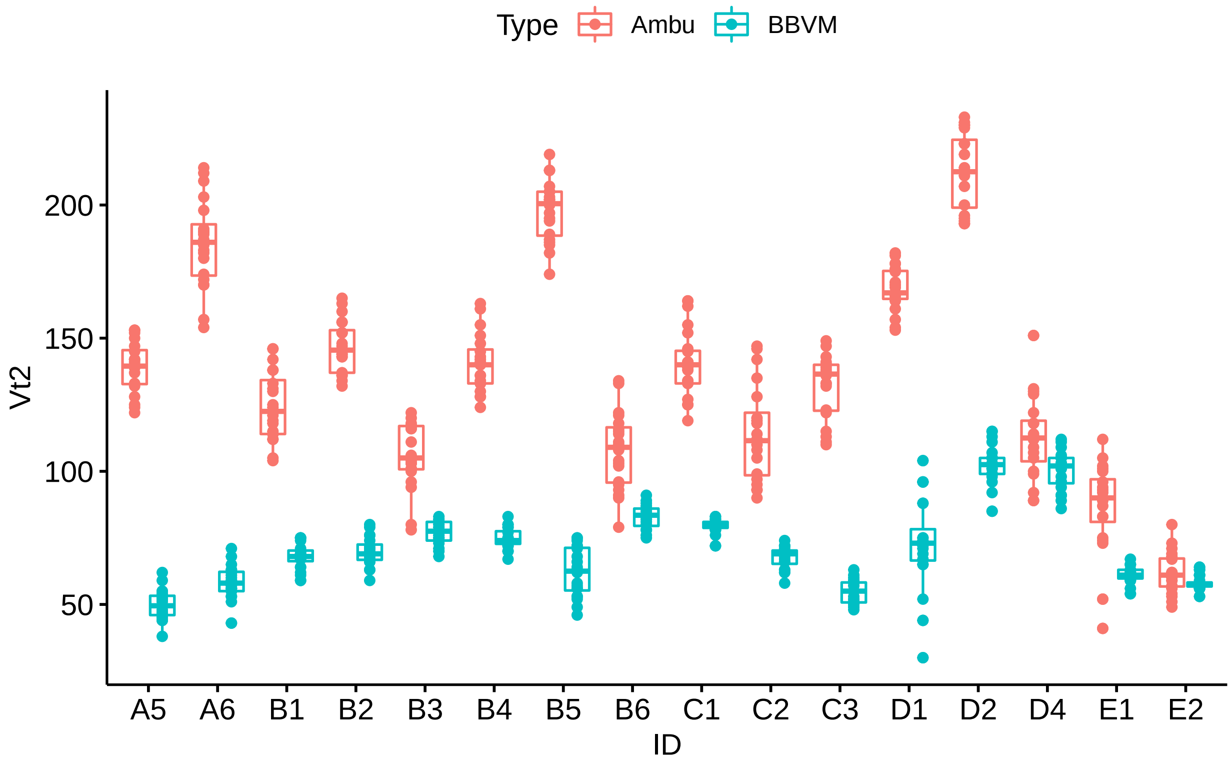

- **Basic test of Normality (Shapiro's Test applied to each Paricipant by Type)**

ID	Type	variable	statistic	p
A5	Ambu	Vt2	0.9484399	0.3440282
A6	Ambu	Vt2	0.9704138	0.7635897

ID	Type	variable	statistic	p
B1	Ambu	Vt2	0.9488458	0.3499101
B2	Ambu	Vt2	0.9522759	0.4029639
B3	Ambu	Vt2	0.9035039	0.0479956
B4	Ambu	Vt2	0.9535974	0.4250111
B5	Ambu	Vt2	0.9797536	0.9308804
B6	Ambu	Vt2	0.9809265	0.9454934
C1	Ambu	Vt2	0.9626463	0.5979635
C2	Ambu	Vt2	0.9296890	0.1523031
C3	Ambu	Vt2	0.8967571	0.0358667
D1	Ambu	Vt2	0.9594376	0.5326765
D2	Ambu	Vt2	0.9080501	0.0585166
D4	Ambu	Vt2	0.9582480	0.5094719
E1	Ambu	Vt2	0.9034277	0.0478371
E2	Ambu	Vt2	0.9689339	0.7322182
A5	BBVM	Vt2	0.9699083	0.7529353
A6	BBVM	Vt2	0.9768310	0.8869821
B1	BBVM	Vt2	0.9462427	0.3136279
B2	BBVM	Vt2	0.9751607	0.8577669
B3	BBVM	Vt2	0.9307495	0.1596305
B4	BBVM	Vt2	0.9602574	0.5490042
B5	BBVM	Vt2	0.9395075	0.2346913
B6	BBVM	Vt2	0.9648292	0.6440972
C1	BBVM	Vt2	0.8920806	0.0293732
C2	BBVM	Vt2	0.9380735	0.2204421
C3	BBVM	Vt2	0.9480353	0.3382502
D1	BBVM	Vt2	0.9360486	0.2017061
D2	BBVM	Vt2	0.9617773	0.5799370
D4	BBVM	Vt2	0.9611706	0.5674928
E1	BBVM	Vt2	0.9623321	0.5914207
E2	BBVM	Vt2	0.8997884	0.0408639

- **Visualizing the differences between the BVM Types per each participant**

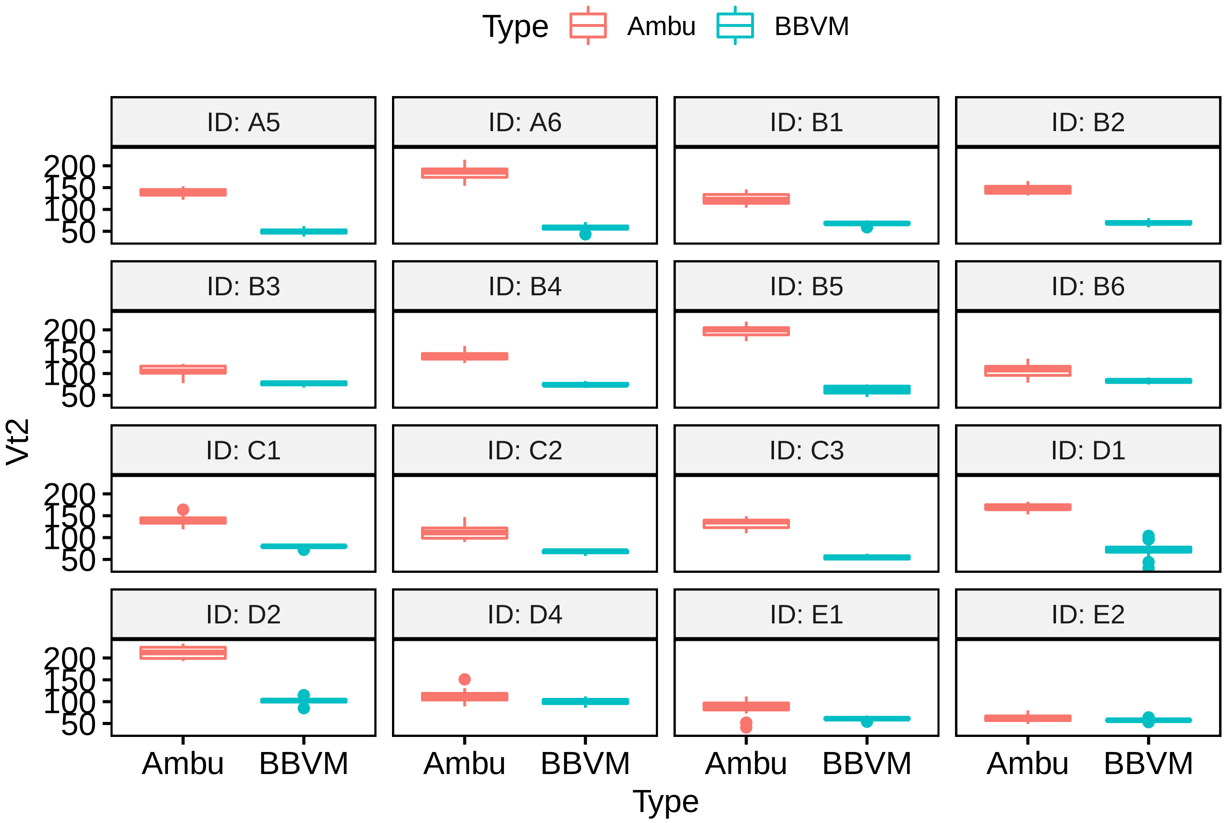

- **Pairwise T-test comparing the BVM Types by each participant**

ID	.y.	group1	group2	n1	n2	statistic	df	p
A5	Vt2	Ambu	BBVM	20	20	37.454437	19	0.00e+00
A6	Vt2	Ambu	BBVM	20	20	33.979028	19	0.00e+00
B1	Vt2	Ambu	BBVM	20	20	19.233851	19	0.00e+00
B2	Vt2	Ambu	BBVM	20	20	29.125171	19	0.00e+00
B3	Vt2	Ambu	BBVM	20	20	9.825978	19	0.00e+00
B4	Vt2	Ambu	BBVM	20	20	27.389151	19	0.00e+00
B5	Vt2	Ambu	BBVM	20	20	43.568305	19	0.00e+00
B6	Vt2	Ambu	BBVM	20	20	7.519453	19	4.00e-07
C1	Vt2	Ambu	BBVM	20	20	21.939650	19	0.00e+00
C2	Vt2	Ambu	BBVM	20	20	10.104017	19	0.00e+00
C3	Vt2	Ambu	BBVM	20	20	26.495534	19	0.00e+00
D1	Vt2	Ambu	BBVM	20	20	18.610975	19	0.00e+00
D2	Vt2	Ambu	BBVM	20	20	26.276904	19	0.00e+00
D4	Vt2	Ambu	BBVM	20	20	3.292816	19	3.83e-03
E1	Vt2	Ambu	BBVM	20	20	7.012007	19	1.10e-06
E2	Vt2	Ambu	BBVM	20	20	2.344780	19	3.01e-02

ANOVA approach for the comparisons

• "Interaction" plot between the Type and the repeated measurements, Trial, on Vt

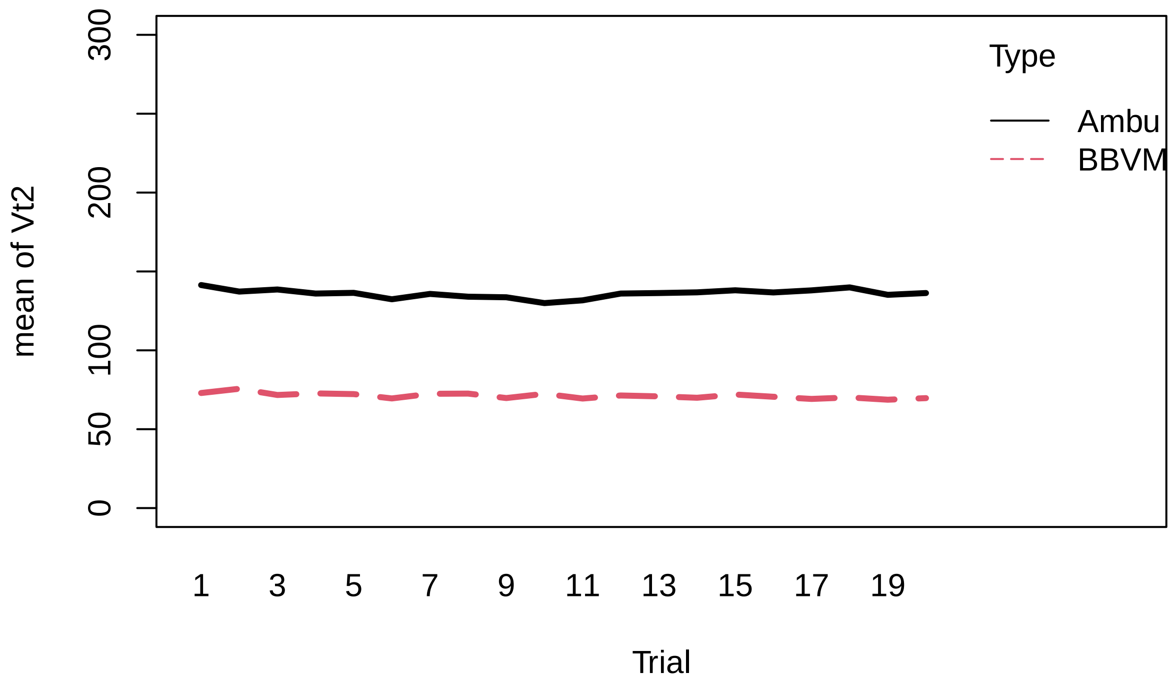

• With Type only and also accounting for the random effects of the Participants.

```
##
## Call:
## aov(formula = Vt2 ~ Type + Error(ID), data = data0)
##
## Grand Mean: 103.5969
##
## Stratum 1: ID
##
## Terms:
##                 Residuals
## Sum of Squares   309014.8
## Deg. of Freedom        15
##
## Residual standard error: 143.5304
##
## Stratum 2: Within
##
```

```
## Terms:
##                      Type Residuals
## Sum of Squares  672364.9  308490.3
## Deg. of Freedom        1       623
##
## Residual standard error: 22.25239
## Estimated effects are balanced
```

```
##           Df Sum Sq Mean Sq F value Pr(>F)
## Residuals 15 309015   20601
```

```
##            Df Sum Sq Mean Sq F value Pr(>F)
## Type        1 672365  672365    1358 <2e-16 ***
## Residuals 623 308490     495
## ---
## Signif. codes:  0 '***' 0.001 '**' 0.01 '*' 0.05 '.' 0.1 ' ' 1
```

- **With Type and Exp and also accounting for the random effects of the Participants (unbalanced case).**

```
##
## Call:
## aov(formula = Vt2 ~ Type + Exp + Error(ID), data = data0)
##
## Grand Mean: 103.5969
##
## Stratum 1: ID
##
## Terms:
##                       Exp Residuals
## Sum of Squares   33799.61 275215.23
## Deg. of Freedom         2        13
##
## Residual standard error: 145.5005
## Estimated effects may be unbalanced
##
## Stratum 2: Within
##
## Terms:
##                      Type Residuals
## Sum of Squares  672364.9  308490.3
## Deg. of Freedom        1       623
##
## Residual standard error: 22.25239
## Estimated effects are balanced
```

```
##           Df Sum Sq Mean Sq F value Pr(>F)
## Exp        2  33800   16900   0.798  0.471
## Residuals 13 275215   21170
```

```
##            Df Sum Sq Mean Sq F value Pr(>F)
## Type        1 672365  672365    1358 <2e-16 ***
```

```
## Residuals 623 308490     495
## ---
## Signif. codes:  0 '***' 0.001 '**' 0.01 '*' 0.05 '.' 0.1 ' ' 1
```

- **With Type and Gender also accounting for the random effects of the Participants (unbalanced case).**

```
##
## Call:
## aov(formula = Vt2 ~ Type + Gender + Error(ID), data = data0)
##
## Grand Mean: 103.5969
##
## Stratum 1: ID
##
## Terms:
##                    Gender Residuals
## Sum of Squares    2202.25 306812.59
## Deg. of Freedom         1        14
##
## Residual standard error: 148.0378
## Estimated effects are balanced
##
## Stratum 2: Within
##
## Terms:
##                     Type Residuals
## Sum of Squares  672364.9  308490.3
## Deg. of Freedom        1       623
##
## Residual standard error: 22.25239
## Estimated effects are balanced
```

```
##           Df Sum Sq Mean Sq F value Pr(>F)
## Gender     1   2202    2202     0.1  0.756
## Residuals 14 306813   21915
```

```
##            Df Sum Sq Mean Sq F value Pr(>F)
## Type        1 672365  672365    1358 <2e-16 ***
## Residuals 623 308490     495
## ---
## Signif. codes:  0 '***' 0.001 '**' 0.01 '*' 0.05 '.' 0.1 ' ' 1
```

• With Type, Exp and Gender also accounting for the random effects of the Participants (unbalanced case).

```
##
## Call:
## aov(formula = Vt2 ~ Type + Gender + Exp + Error(ID), data = data0)
##
## Grand Mean: 103.5969
##
## Stratum 1: ID
##
## Terms:
##                    Gender       Exp Residuals
## Sum of Squares    2202.25  55016.11 251796.48
## Deg. of Freedom         1         2        12
##
## Residual standard error: 144.8552
## Estimated effects may be unbalanced
##
## Stratum 2: Within
##
## Terms:
##                      Type Residuals
## Sum of Squares  672364.9  308490.3
## Deg. of Freedom        1       623
##
## Residual standard error: 22.25239
## Estimated effects are balanced
```

```
##           Df Sum Sq Mean Sq F value Pr(>F)
## Gender     1   2202    2202   0.105  0.752
## Exp        2  55016   27508   1.311  0.306
## Residuals 12 251796   20983
```

```
##            Df Sum Sq Mean Sq F value Pr(>F)
## Type        1 672365  672365    1358 <2e-16 ***
## Residuals 623 308490     495
## ---
## Signif. codes:  0 '***' 0.001 '**' 0.01 '*' 0.05 '.' 0.1 ' ' 1
```
