# Supplementary material for: Improved simulated ventilation with a novel tidal volume and peak inspiratory pressure controlling bag valve mask: A pilot study
Source: Resusc Plus. 2023 Jan 5;13:100350. doi: 10.1016/j.resplu.2022.100350 (PMC9841173; doi:10.1016/j.resplu.2022.100350)
Supplement: Supplementary data 8 [file mmc8.pdf]

Comparing Ambu versus BBVM*

The Pediatric Mannequin Trial

Supplement #7, Analysis of PIP under the **High PIP** Condition

Summary: Experiment setting # 3, PIP Measurements

• Setting up the Data Frame (PIP Measurements)

```
## 'data.frame':    320 obs. of  6 variables:
##  $ ID    : Factor w/ 16 levels "A5","A6","B1",..: 1 1 1 1 1 1 1 1 1 1 ...
##  $ Gender: Factor w/ 2 levels "F","M": 1 1 1 1 1 1 1 1 1 1 ...
##  $ Exp   : Factor w/ 3 levels "T1","T2","T3": 1 1 1 1 1 1 1 1 1 1 ...
##  $ Trial : int  1 2 3 4 5 6 7 8 9 10 ...
##  $ Ambu  : num  23.7 23.7 23.7 26 22.7 22.7 22.2 25.6 27.5 23.7 ...
##  $ BBVM  : num  15.1 15.6 14.7 9.9 11.4 14.2 13.7 12.3 13.2 11.4 ...
```

• The Structure of the Pediatric Data

ID	Gender	Exp	Trial	Ambu	BBVM
A5	F	T1	1	23.7	15.1
A5	F	T1	2	23.7	15.6
A5	F	T1	3	23.7	14.7
A5	F	T1	4	26.0	9.9
A5	F	T1	5	22.7	11.4

• Changing the data frame from a wide format to a Long Style and removing the missing participent "C2"

```
## 'data.frame':    600 obs. of  6 variables:
##  $ ID    : Factor w/ 16 levels "A5","A6","B1",..: 1 1 1 1 1 1 1 1 1 1 ...
##  $ Gender: Factor w/ 2 levels "F","M": 1 1 1 1 1 1 1 1 1 1 ...
##  $ Exp   : Factor w/ 3 levels "T1","T2","T3": 1 1 1 1 1 1 1 1 1 1 ...
##  $ Trial : int  1 2 3 4 5 6 7 8 9 10 ...
##  $ Type  : Factor w/ 2 levels "Ambu","BBVM": 1 1 1 1 1 1 1 1 1 1 ...
##  $ PIP3  : num  23.7 23.7 23.7 26 22.7 22.7 22.2 25.6 27.5 23.7 ...
```

*Supplemental Report to the *Improved Ventilation with a Novel Tidal Volume and Peak Inspiratory Pressure Controlling Bag Valve Mask–A Pilot Study*

- **The numberof participants per each Gender by Experience group**

	F	M
T1	6	4
T2	3	0
T3	0	2

- **The sample sizes per each Gender by Experience group**

Exp	Gender	n	prop
T1	F	240	60
T1	M	160	40
T2	F	120	100
T3	M	80	100

- **Summary statistics for PIP_3 by the two BVM types (while ignoring all other factors)**

Type	variable	n	min	max	median	iqr	mean	sd	se	ci
Ambu	PIP3	300	16.60	37.9	26.5	3.2	26.465	3.009	0.174	0.342
BBVM	PIP3	300	8.99	30.4	21.8	10.1	20.302	5.629	0.325	0.640

- **Visualizing the Distrubution of PIP_3 by the two BVM Types (while ignoring all other factors)**

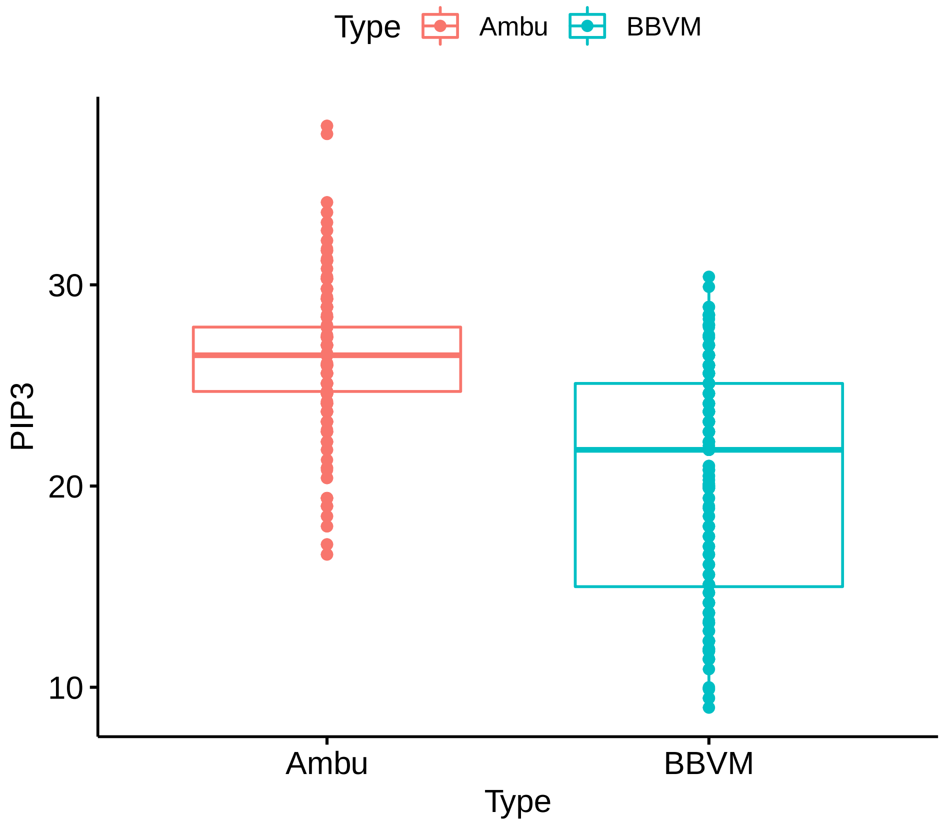

- **Summary statistics for PIP_3 by Gender and Type**

Gender	Type	variable	n	min	max	median	iqr	mean	sd	se	ci
F	Ambu	PIP3	180	16.60	33.6	26.0	2.800	25.842	2.888	0.215	0.425
M	Ambu	PIP3	120	22.70	37.9	27.4	3.825	27.401	2.955	0.270	0.534
F	BBVM	PIP3	180	8.99	26.0	16.1	7.700	16.958	4.553	0.339	0.670
M	BBVM	PIP3	120	15.10	30.4	26.0	3.300	25.319	2.476	0.226	0.447

- **Visualizing the Distrubution of PIP_3 by Type for each Gender**

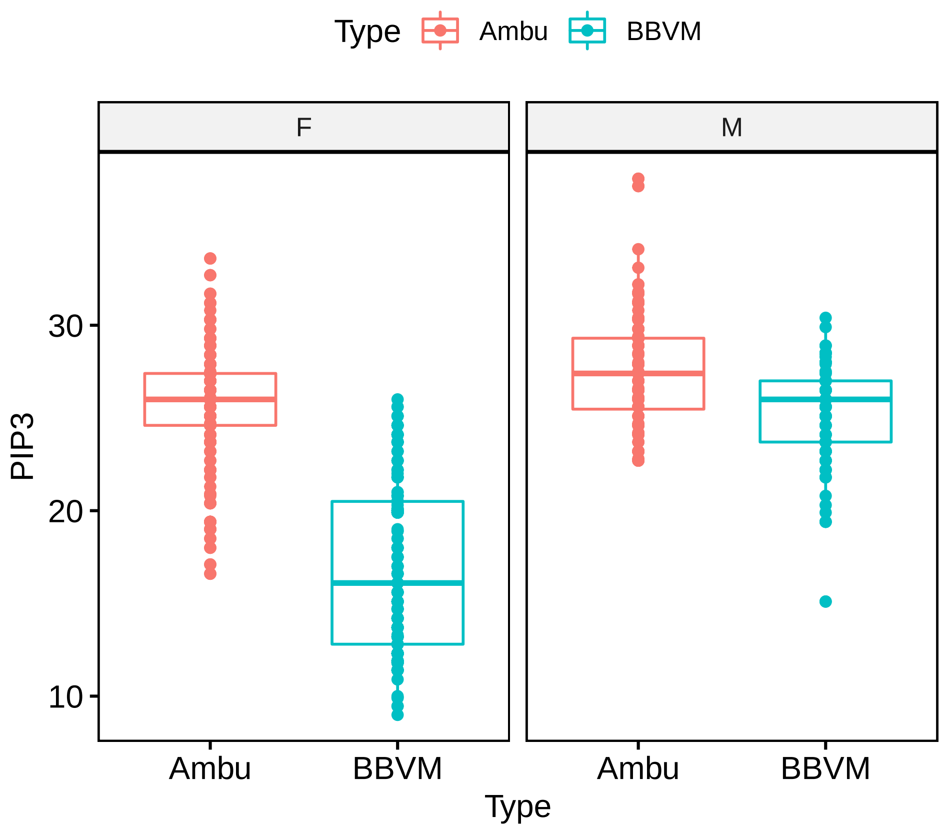

- **Summary statistics for PIP_3 by Type and Years of Expereince**

Exp	Type	variable	n	min	max	median	iqr	mean	sd	se	ci
T1	Ambu	PIP3	200	16.60	37.9	26.5	4.325	26.360	3.467	0.245	0.483
T2	Ambu	PIP3	60	23.70	31.7	26.5	1.800	26.653	1.433	0.185	0.370
T3	Ambu	PIP3	40	22.70	31.7	26.5	2.800	26.710	2.188	0.346	0.700
T1	BBVM	PIP3	200	9.90	30.4	20.5	10.000	20.466	5.631	0.398	0.785
T2	BBVM	PIP3	60	8.99	26.0	15.1	10.225	17.044	5.338	0.689	1.379
T3	BBVM	PIP3	40	15.10	27.0	24.6	2.800	24.372	2.127	0.336	0.680

- **Visualizing the Distrubution of PIP_3 by Type and Years of Expereince**

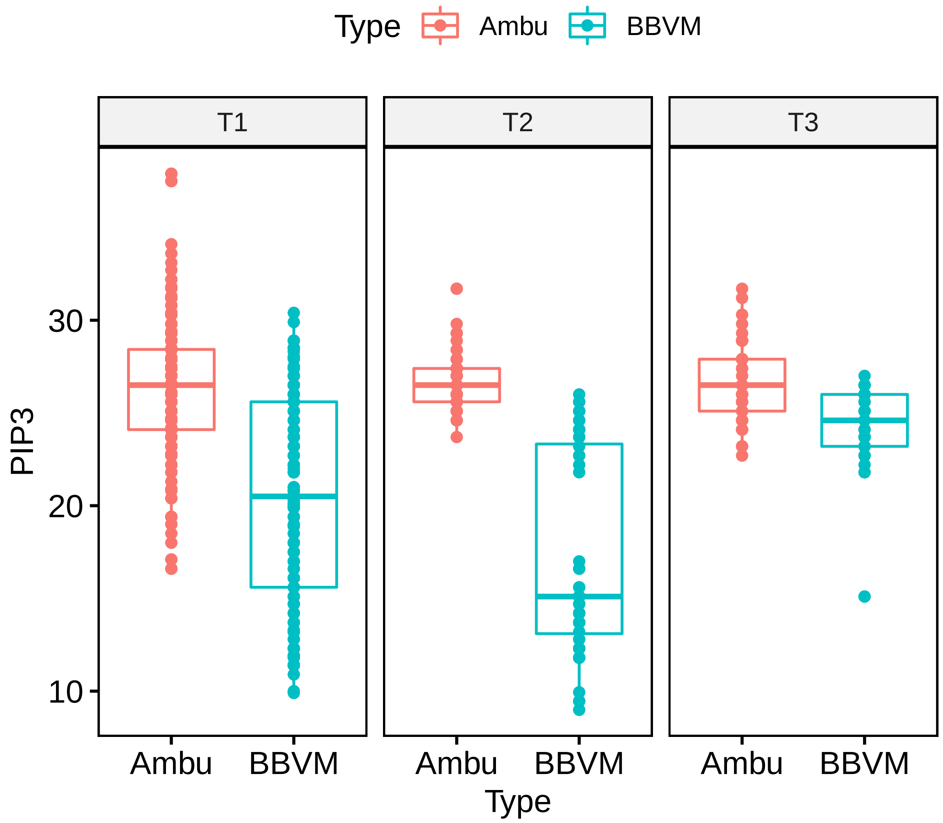

- **Summary statistics of PIP_3 by Participants and Type**

ID	Type	variable	n	min	max	median	iqr	mean	sd	se	ci
A5	Ambu	PIP3	20	22.20	27.5	23.90	2.150	24.280	1.573	0.352	0.736
A6	Ambu	PIP3	20	23.70	33.6	29.30	3.900	28.765	2.701	0.604	1.264
B1	Ambu	PIP3	20	26.00	30.3	27.40	2.025	27.655	1.311	0.293	0.614
B2	Ambu	PIP3	20	26.00	30.3	27.65	0.900	27.620	1.000	0.224	0.468
B3	Ambu	PIP3	20	22.20	30.3	25.60	1.025	25.730	1.719	0.384	0.804
B4	Ambu	PIP3	20	28.90	37.9	30.55	2.150	31.040	2.163	0.484	1.012
B5	Ambu	PIP3	20	22.80	37.5	26.80	5.075	27.425	3.640	0.814	1.704
B6	Ambu	PIP3	20	16.60	27.0	20.90	4.950	21.415	3.071	0.687	1.437
C1	Ambu	PIP3	20	22.70	26.5	25.10	1.900	25.010	1.187	0.265	0.556
C3	Ambu	PIP3	20	25.10	31.7	26.75	1.900	27.180	1.536	0.343	0.719
D1	Ambu	PIP3	20	22.70	29.8	24.60	2.625	24.900	2.091	0.468	0.979
D2	Ambu	PIP3	20	25.10	29.8	27.20	1.400	26.960	1.094	0.245	0.512
D4	Ambu	PIP3	20	19.40	30.3	24.85	4.150	24.770	3.059	0.684	1.431
E1	Ambu	PIP3	20	23.70	29.3	25.60	1.150	25.820	1.304	0.292	0.611
E2	Ambu	PIP3	20	26.00	31.7	27.90	1.600	28.410	1.527	0.341	0.715
A5	BBVM	PIP3	20	9.90	15.6	12.55	1.625	12.770	1.421	0.318	0.665
A6	BBVM	PIP3	20	12.30	23.7	16.80	4.400	16.895	2.934	0.656	1.373
B1	BBVM	PIP3	20	17.00	22.0	20.00	0.525	19.835	1.241	0.277	0.581

ID	Type	variable	n	min	max	median	iqr	mean	sd	se	ci
B2	BBVM	PIP3	20	25.60	28.3	26.50	1.000	26.565	0.767	0.171	0.359
B3	BBVM	PIP3	20	15.60	24.6	22.45	2.150	22.100	2.267	0.507	1.061
B4	BBVM	PIP3	20	24.10	27.0	26.00	1.400	25.870	0.788	0.176	0.369
B5	BBVM	PIP3	20	27.00	30.4	28.50	0.600	28.425	0.776	0.173	0.363
B6	BBVM	PIP3	20	10.00	13.3	11.90	0.900	11.825	0.694	0.155	0.325
C1	BBVM	PIP3	20	15.10	26.5	23.70	1.400	23.520	2.266	0.507	1.060
C3	BBVM	PIP3	20	12.30	17.0	14.20	1.525	14.180	1.238	0.277	0.580
D1	BBVM	PIP3	20	19.40	25.6	22.45	2.650	22.310	1.940	0.434	0.908
D2	BBVM	PIP3	20	8.99	16.6	12.55	2.400	12.818	2.283	0.510	1.068
D4	BBVM	PIP3	20	14.70	23.2	17.75	3.425	18.065	2.225	0.498	1.041
E1	BBVM	PIP3	20	21.80	26.0	24.10	1.525	24.135	1.165	0.260	0.545
E2	BBVM	PIP3	20	21.80	27.0	26.00	1.650	25.225	1.619	0.362	0.758

- **Visualizing the Distrubution of PIP_3 by Participants and Type**

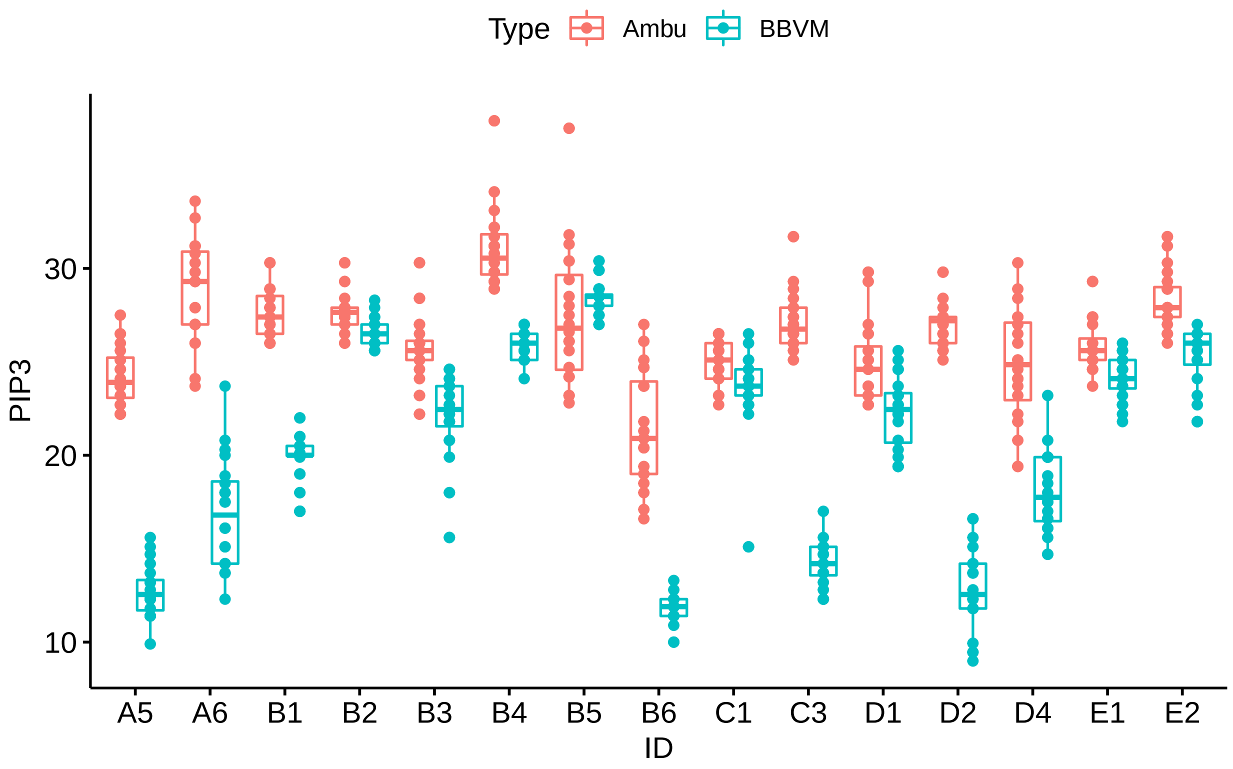

- **Basic test of Normality (Shapiro's Test applied to each Paricipant by Type)**

ID	Type	variable	statistic	p
A5	Ambu	PIP3	0.9472666	0.3274952
A6	Ambu	PIP3	0.9725832	0.8082237
B1	Ambu	PIP3	0.9249952	0.1236679
B2	Ambu	PIP3	0.9089464	0.0608583

ID	Type	variable	statistic	p
B3	Ambu	PIP3	0.9300906	0.1550384
B4	Ambu	PIP3	0.8245172	0.0020533
B5	Ambu	PIP3	0.9243903	0.1203915
B6	Ambu	PIP3	0.9560713	0.4686455
C1	Ambu	PIP3	0.9311643	0.1625900
C3	Ambu	PIP3	0.8896177	0.0264607
D1	Ambu	PIP3	0.8798707	0.0175980
D2	Ambu	PIP3	0.9336443	0.1814318
D4	Ambu	PIP3	0.9772175	0.8933481
E1	Ambu	PIP3	0.9080674	0.0585608
E2	Ambu	PIP3	0.9540688	0.4330887
A5	BBVM	PIP3	0.9666254	0.6826351
A6	BBVM	PIP3	0.9586870	0.5179635
B1	BBVM	PIP3	0.8286177	0.0023848
B2	BBVM	PIP3	0.9224248	0.1103343
B3	BBVM	PIP3	0.8681317	0.0108954
B4	BBVM	PIP3	0.9347000	0.1900802
B5	BBVM	PIP3	0.9132872	0.0736454
B6	BBVM	PIP3	0.9270923	0.1357328
C1	BBVM	PIP3	0.7080860	0.0000490
C3	BBVM	PIP3	0.9441929	0.2873945
D1	BBVM	PIP3	0.9428512	0.2712990
D2	BBVM	PIP3	0.9531951	0.4182050
D4	BBVM	PIP3	0.9548902	0.4474348
E1	BBVM	PIP3	0.9614020	0.5722247
E2	BBVM	PIP3	0.8125718	0.0013386

- **Visualizing the differences between the BVM Types per each participant**

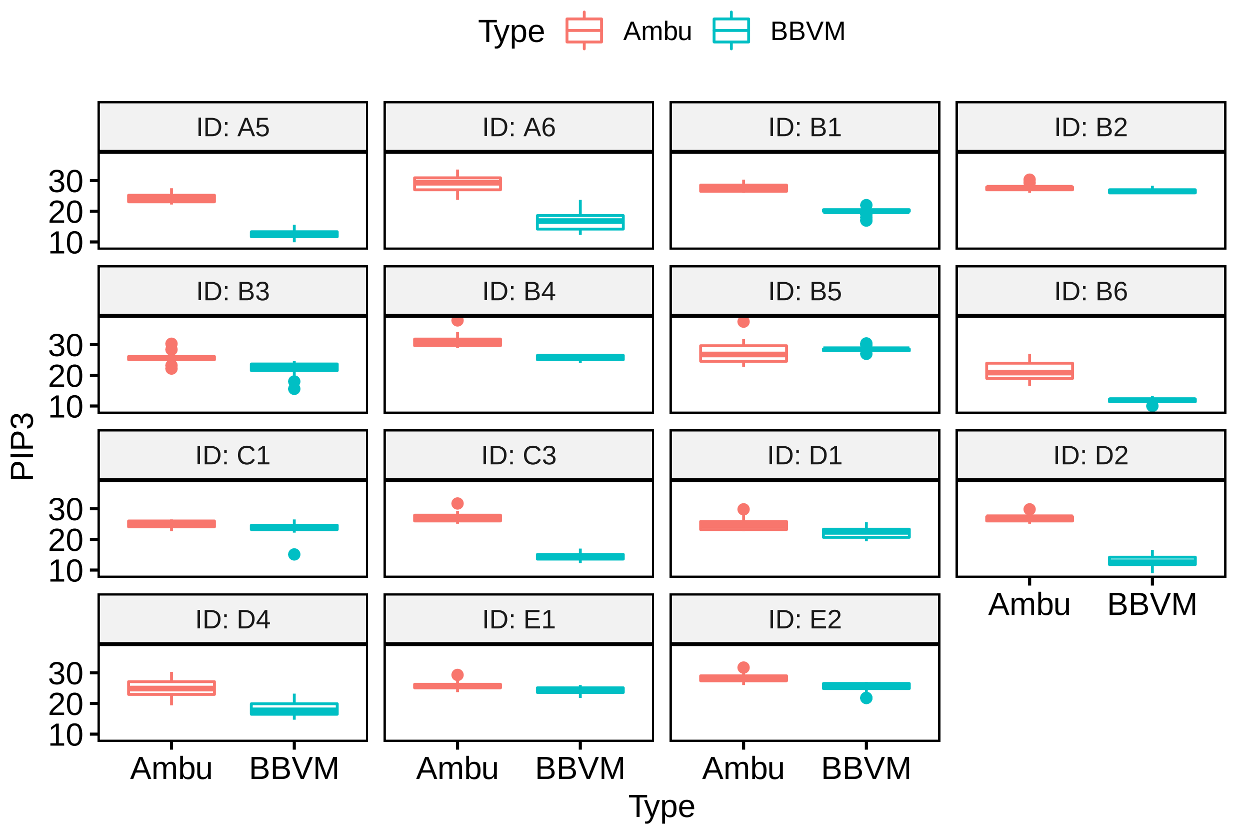

- **Pairwise T-test comparing the BVM Types by each participant**

ID	.y.	group1	group2	n1	n2	statistic	df	p
A5	PIP3	Ambu	BBVM	20	20	23.443590	19	0.00e+00
A6	PIP3	Ambu	BBVM	20	20	11.320282	19	0.00e+00
B1	PIP3	Ambu	BBVM	20	20	18.627386	19	0.00e+00
B2	PIP3	Ambu	BBVM	20	20	3.662568	19	1.66e-03
B3	PIP3	Ambu	BBVM	20	20	4.949680	19	8.90e-05
B4	PIP3	Ambu	BBVM	20	20	11.888654	19	0.00e+00
B5	PIP3	Ambu	BBVM	20	20	-1.252578	19	2.26e-01
B6	PIP3	Ambu	BBVM	20	20	13.256139	19	0.00e+00
C1	PIP3	Ambu	BBVM	20	20	3.007381	19	7.24e-03
C3	PIP3	Ambu	BBVM	20	20	27.802696	19	0.00e+00
D1	PIP3	Ambu	BBVM	20	20	5.258124	19	4.48e-05
D2	PIP3	Ambu	BBVM	20	20	25.168150	19	0.00e+00
D4	PIP3	Ambu	BBVM	20	20	10.311548	19	0.00e+00
E1	PIP3	Ambu	BBVM	20	20	4.804208	19	1.23e-04
E2	PIP3	Ambu	BBVM	20	20	6.631876	19	2.40e-06

ANOVA approach for the comparisons

- **"Interaction" plot between the Type and the repeated measurements, Trial, on Vt**

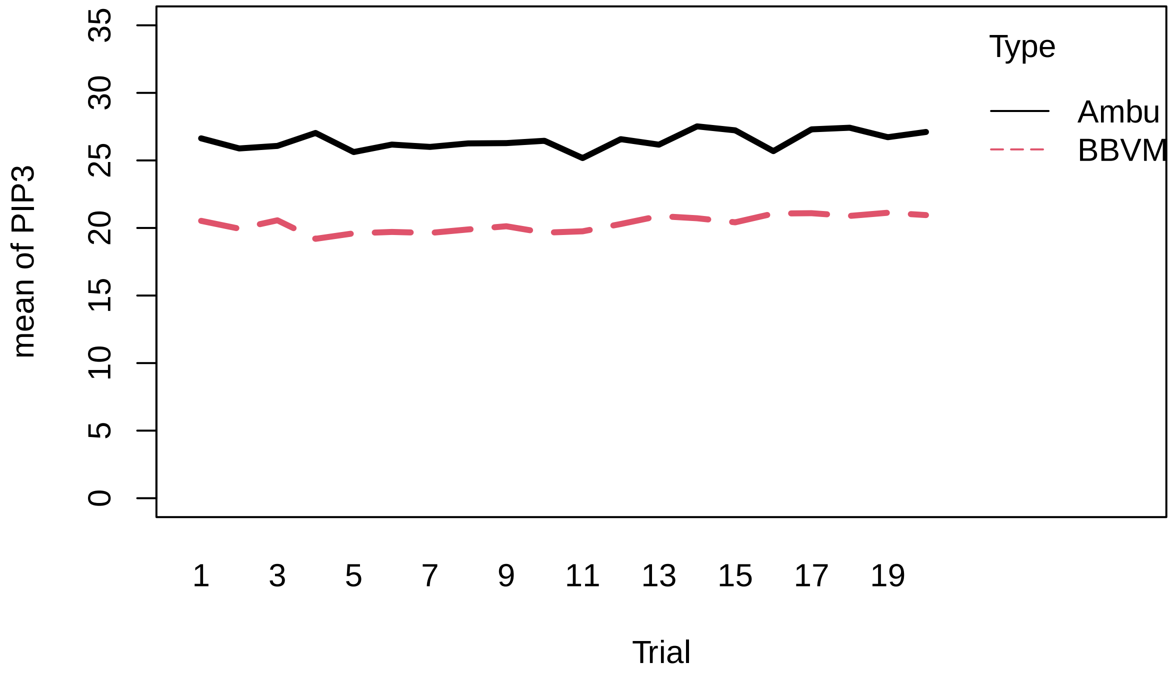

- **With Type only and also accounting for the random effects of the Participants.**

```
##
## Call:
## aov(formula = PIP3 ~ Type + Error(ID), data = data0)
##
## Grand Mean: 23.38392
##
## Stratum 1: ID
##
## Terms:
##                  Residuals
## Sum of Squares    6748.726
## Deg. of Freedom         14
##
## Residual standard error: 21.95568
##
## Stratum 2: Within
##
```

```
## Terms:
##                     Type Residuals
## Sum of Squares  5697.077  5431.259
## Deg. of Freedom        1       584
##
## Residual standard error: 3.049607
## Estimated effects are balanced
```

```
##           Df Sum Sq Mean Sq F value Pr(>F)
## Residuals 14   6749   482.1
```

```
##            Df Sum Sq Mean Sq F value Pr(>F)
## Type        1   5697    5697   612.6 <2e-16 ***
## Residuals 584   5431       9
## ---
## Signif. codes:  0 '***' 0.001 '**' 0.01 '*' 0.05 '.' 0.1 ' ' 1
```

- **With Type and Exp and also accounting for the random effects of the Participants (unbalanced case).**

```
##
## Call:
## aov(formula = PIP3 ~ Type + Exp + Error(ID), data = data0)
##
## Grand Mean: 23.38392
##
## Stratum 1: ID
##
## Terms:
##                      Exp Residuals
## Sum of Squares   655.474  6093.252
## Deg. of Freedom        2        12
##
## Residual standard error: 22.53377
## Estimated effects may be unbalanced
##
## Stratum 2: Within
##
## Terms:
##                     Type Residuals
## Sum of Squares  5697.077  5431.259
## Deg. of Freedom        1       584
##
## Residual standard error: 3.049607
## Estimated effects are balanced
```

```
##           Df Sum Sq Mean Sq F value Pr(>F)
## Exp        2    655   327.7   0.645  0.542
## Residuals 12   6093   507.8
```

```
##            Df Sum Sq Mean Sq F value Pr(>F)
## Type        1   5697    5697   612.6 <2e-16 ***
```

```
## Residuals 584   5431       9
## ---
## Signif. codes:  0 '***' 0.001 '**' 0.01 '*' 0.05 '.' 0.1 ' ' 1
```

- **With Type and Gender also accounting for the random effects of the Participants (unbalanced case).**

```
##
## Call:
## aov(formula = PIP3 ~ Type + Gender + Error(ID), data = data0)
##
## Grand Mean: 23.38392
##
## Stratum 1: ID
##
## Terms:
##                   Gender Residuals
## Sum of Squares  3542.829  3205.897
## Deg. of Freedom        1        13
##
## Residual standard error: 15.70374
## Estimated effects are balanced
##
## Stratum 2: Within
##
## Terms:
##                     Type Residuals
## Sum of Squares  5697.077  5431.259
## Deg. of Freedom        1       584
##
## Residual standard error: 3.049607
## Estimated effects are balanced
```

```
##           Df Sum Sq Mean Sq F value  Pr(>F)
## Gender     1   3543    3543   14.37 0.00225 **
## Residuals 13   3206     247
## ---
## Signif. codes:  0 '***' 0.001 '**' 0.01 '*' 0.05 '.' 0.1 ' ' 1
```

```
##            Df Sum Sq Mean Sq F value Pr(>F)
## Type        1   5697    5697   612.6 <2e-16 ***
## Residuals 584   5431       9
## ---
## Signif. codes:  0 '***' 0.001 '**' 0.01 '*' 0.05 '.' 0.1 ' ' 1
```

- **With Type, Exp and Gender also accounting for the random effects of the Participants (unbalanced case).**

```
##
## Call:
## aov(formula = PIP3 ~ Type + Gender + Exp + Error(ID), data = data0)
##
## Grand Mean: 23.38392
##
## Stratum 1: ID
##
## Terms:
##                   Gender      Exp Residuals
## Sum of Squares  3542.829  116.712  3089.185
## Deg. of Freedom        1        2        11
##
## Residual standard error: 16.75813
## Estimated effects may be unbalanced
##
## Stratum 2: Within
##
## Terms:
##                     Type Residuals
## Sum of Squares  5697.077  5431.259
## Deg. of Freedom        1       584
##
## Residual standard error: 3.049607
## Estimated effects are balanced
```

```
##           Df Sum Sq Mean Sq F value  Pr(>F)
## Gender     1   3543    3543  12.615 0.00454 **
## Exp        2    117      58   0.208 0.81549
## Residuals 11   3089     281
## ---
## Signif. codes:  0 '***' 0.001 '**' 0.01 '*' 0.05 '.' 0.1 ' ' 1
```

```
##            Df Sum Sq Mean Sq F value Pr(>F)
## Type        1   5697    5697   612.6 <2e-16 ***
## Residuals 584   5431       9
## ---
## Signif. codes:  0 '***' 0.001 '**' 0.01 '*' 0.05 '.' 0.1 ' ' 1
```
