# Supplementary material for: Improved simulated ventilation with a novel tidal volume and peak inspiratory pressure controlling bag valve mask: A pilot study
Source: Resusc Plus. 2023 Jan 5;13:100350. doi: 10.1016/j.resplu.2022.100350 (PMC9841173; doi:10.1016/j.resplu.2022.100350)
Supplement: Supplementary data 9 [file mmc9.pdf]

Comparing Ambu versus BBVM*

The Pediatric Mannequin Trial

Supplement #8, Analysis of Vt under the **High PIP** Condition

Summary: Experiment setting # 3, Vt Measurements

• Setting up the Data Frame (Vt Measurements)

```
## 'data.frame':    320 obs. of  6 variables:
##  $ ID    : Factor w/ 16 levels "A5","A6","B1",..: 1 1 1 1 1 1 1 1 1 1 ...
##  $ Gender: Factor w/ 2 levels "F","M": 1 1 1 1 1 1 1 1 1 1 ...
##  $ Exp   : Factor w/ 3 levels "T1","T2","T3": 1 1 1 1 1 1 1 1 1 1 ...
##  $ Trial : int  1 2 3 4 5 6 7 8 9 10 ...
##  $ Ambu  : int  158 144 161 146 128 149 153 145 138 144 ...
##  $ BBVM  : int  65 71 69 66 46 51 64 58 49 52 ...
```

• The Structure of the Pediatric Data

ID	Gender	Exp	Trial	Ambu	BBVM
A5	F	T1	1	158	65
A5	F	T1	2	144	71
A5	F	T1	3	161	69
A5	F	T1	4	146	66
A5	F	T1	5	128	46

• Changing the data frame from a wide format to a Long Style

```
## 'data.frame':    640 obs. of  6 variables:
##  $ ID    : Factor w/ 16 levels "A5","A6","B1",..: 1 1 1 1 1 1 1 1 1 1 ...
##  $ Gender: Factor w/ 2 levels "F","M": 1 1 1 1 1 1 1 1 1 1 ...
##  $ Exp   : Factor w/ 3 levels "T1","T2","T3": 1 1 1 1 1 1 1 1 1 1 ...
##  $ Trial : int  1 2 3 4 5 6 7 8 9 10 ...
##  $ Type  : Factor w/ 2 levels "Ambu","BBVM": 1 1 1 1 1 1 1 1 1 1 ...
##  $ Vt3   : int  158 144 161 146 128 149 153 145 138 144 ...
```

*Supplemental Report to the *Improved Ventilation with a Novel Tidal Volume and Peak Inspiratory Pressure Controlling Bag Valve Mask–A Pilot Study*

- **The numberof participants per each Gender by Experience group**

	F	M
T1	6	4
T2	4	0
T3	0	2

- **The sample sizes per each Gender by Experience group**

Exp	Gender	n	prop
T1	F	240	60
T1	M	160	40
T2	F	160	100
T3	M	80	100

- **Summary statistics for Vt_3 by the two BVM types (while ignoring all other factors)**

Type	variable	n	min	max	median	iqr	mean	sd	se	ci
Ambu	Vt3	320	46	329	176.5	62.5	173.016	61.239	3.423	6.735
BBVM	Vt3	320	46	127	82.5	27.0	83.722	18.893	1.056	2.078

- **Visualizing the Distrubution of Vt_3 by the two BVM Types (while ignoring all other factors)**

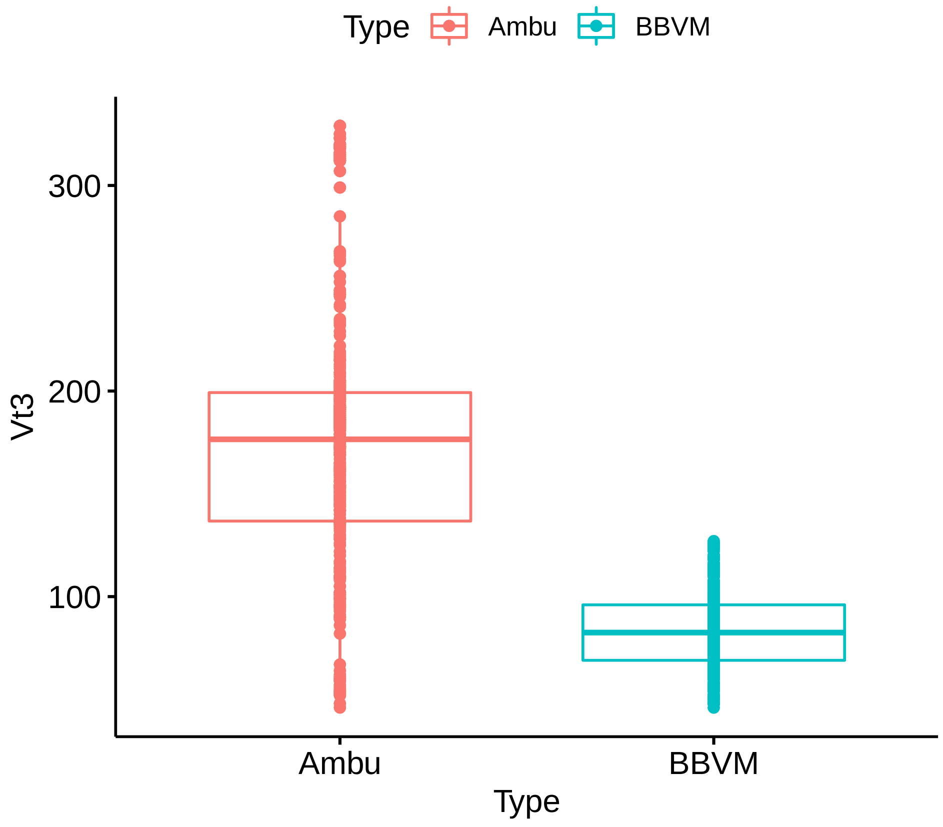

- **Summary statistics for Vt_3 by Gender and Type**

Gender	Type	variable	n	min	max	median	iqr	mean	sd	se	ci
F	Ambu	Vt3	200	46	329	173	65.75	173.980	70.230	4.966	9.793
M	Ambu	Vt3	120	86	264	182	57.00	171.408	42.438	3.874	7.671
F	BBVM	Vt3	200	46	127	79	29.25	82.305	20.338	1.438	2.836
M	BBVM	Vt3	120	52	124	86	18.00	86.083	16.004	1.461	2.893

- **Visualizing the Distrubution of Vt_3 by Type for each Gender**

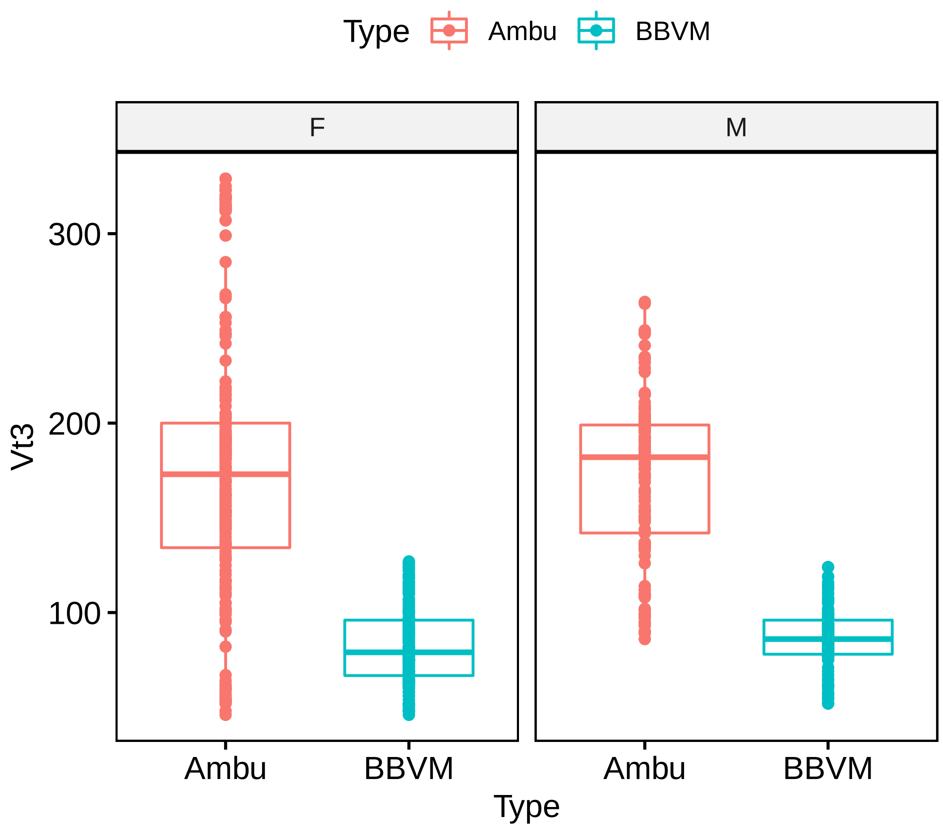

- **Summary statistics for Vt_3 by Type and Years of Expereince**

Exp	Type	variable	n	min	max	median	iqr	mean	sd	se	ci
T1	Ambu	Vt3	200	99	299	181.0	49.00	181.645	36.935	2.612	5.150
T2	Ambu	Vt3	80	46	329	142.0	144.75	165.625	99.932	11.173	22.239
T3	Ambu	Vt3	40	86	216	142.5	89.50	144.650	46.542	7.359	14.885
T1	BBVM	Vt3	200	46	127	84.5	33.00	83.270	21.108	1.493	2.943
T2	BBVM	Vt3	80	56	126	78.5	12.75	82.500	16.712	1.868	3.719
T3	BBVM	Vt3	40	75	101	88.5	10.25	88.425	7.324	1.158	2.342

- **Visualizing the Distrubution of Vt_3 by Type and Years of Expereince**

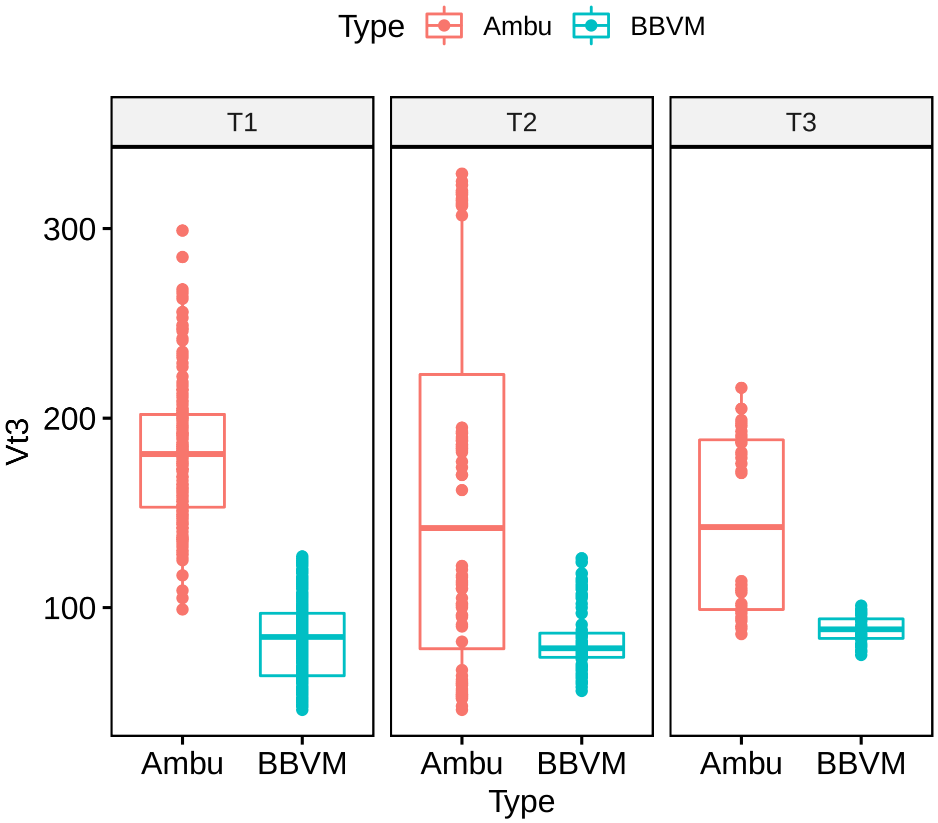

- **Summary statistics of Vt_3 by Participants and Type**

ID	Type	variable	n	min	max	median	iqr	mean	sd	se	ci
A5	Ambu	Vt3	20	125	162	145.5	15.50	146.30	10.593	2.369	4.958
A6	Ambu	Vt3	20	181	299	247.0	41.00	239.80	33.164	7.416	15.521
B1	Ambu	Vt3	20	148	218	182.0	20.50	183.35	19.008	4.250	8.896
B2	Ambu	Vt3	20	169	211	189.0	17.50	191.20	11.606	2.595	5.432
B3	Ambu	Vt3	20	172	219	192.5	18.75	194.40	14.435	3.228	6.756
B4	Ambu	Vt3	20	126	173	142.0	15.75	144.45	11.700	2.616	5.476
B5	Ambu	Vt3	20	144	208	178.5	25.75	177.95	16.330	3.652	7.643
B6	Ambu	Vt3	20	135	209	171.0	36.00	173.95	21.690	4.850	10.151
C1	Ambu	Vt3	20	171	216	189.0	14.50	189.60	11.114	2.485	5.201
C2	Ambu	Vt3	20	46	67	55.5	7.00	56.30	5.243	1.172	2.454
C3	Ambu	Vt3	20	162	195	185.5	8.50	183.50	8.757	1.958	4.098
D1	Ambu	Vt3	20	197	264	228.0	38.25	225.55	21.905	4.898	10.252
D2	Ambu	Vt3	20	307	329	318.0	7.00	318.00	5.767	1.290	2.699
D4	Ambu	Vt3	20	99	175	141.0	25.75	139.50	20.992	4.694	9.825
E1	Ambu	Vt3	20	82	122	103.5	17.25	104.70	10.984	2.456	5.141
E2	Ambu	Vt3	20	86	114	99.0	14.50	99.70	8.310	1.858	3.889
A5	BBVM	Vt3	20	46	71	52.0	11.25	55.60	7.721	1.727	3.614
A6	BBVM	Vt3	20	48	84	69.5	11.50	67.05	10.420	2.330	4.877

ID	Type	variable	n	min	max	median	iqr	mean	sd	se	ci
B1	BBVM	Vt3	20	86	97	90.5	3.00	90.55	2.724	0.609	1.275
B2	BBVM	Vt3	20	77	100	90.0	7.25	88.85	5.752	1.286	2.692
B3	BBVM	Vt3	20	90	103	96.5	4.50	96.60	3.393	0.759	1.588
B4	BBVM	Vt3	20	75	94	81.5	5.50	81.85	4.464	0.998	2.089
B5	BBVM	Vt3	20	52	71	59.0	7.50	59.50	5.680	1.270	2.658
B6	BBVM	Vt3	20	58	80	64.0	5.50	65.35	5.204	1.164	2.436
C1	BBVM	Vt3	20	81	101	93.5	7.25	92.90	5.251	1.174	2.457
C2	BBVM	Vt3	20	74	88	80.5	3.50	80.35	3.774	0.844	1.766
C3	BBVM	Vt3	20	56	75	65.0	8.00	65.15	4.998	1.118	2.339
D1	BBVM	Vt3	20	96	124	109.0	8.50	109.45	7.193	1.608	3.366
D2	BBVM	Vt3	20	81	126	110.0	12.50	107.05	11.409	2.551	5.339
D4	BBVM	Vt3	20	104	127	119.0	7.00	117.90	6.172	1.380	2.889
E1	BBVM	Vt3	20	73	85	77.5	3.50	77.45	2.982	0.667	1.396
E2	BBVM	Vt3	20	75	98	84.5	8.00	83.95	6.353	1.421	2.973

- **Visualizing the Distrubution of Vt_3 by Participants and Type**

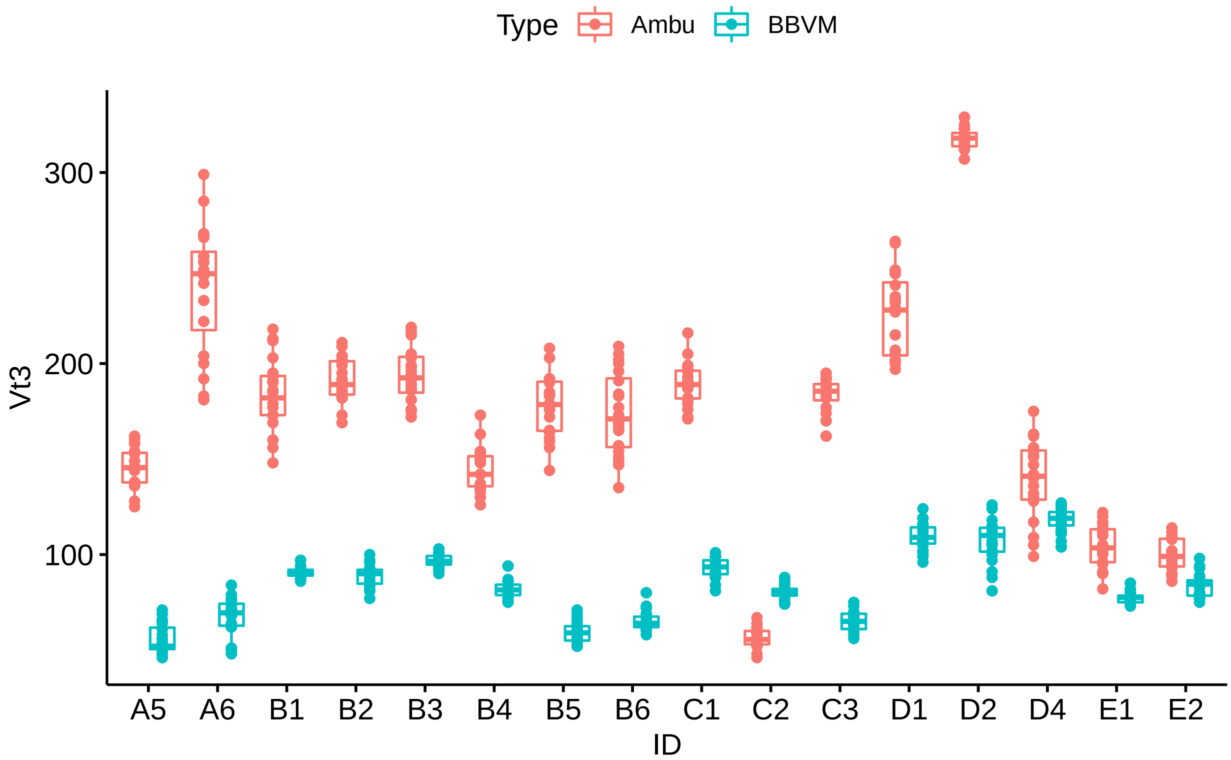

- **Basic test of Normality (Shapiro's Test applied to each Paricipant by Type)**

ID	Type	variable	statistic	p
A5	Ambu	Vt3	0.9583449	0.5113384
A6	Ambu	Vt3	0.9420052	0.2615727

ID	Type	variable	statistic	p
B1	Ambu	Vt3	0.9732545	0.8215605
B2	Ambu	Vt3	0.9609155	0.5622969
B3	Ambu	Vt3	0.9501427	0.3692604
B4	Ambu	Vt3	0.9561419	0.4699344
B5	Ambu	Vt3	0.9829464	0.9662429
B6	Ambu	Vt3	0.9626265	0.5975508
C1	Ambu	Vt3	0.9744738	0.8450338
C2	Ambu	Vt3	0.9815872	0.9529041
C3	Ambu	Vt3	0.9100666	0.0639219
D1	Ambu	Vt3	0.9212124	0.1045571
D2	Ambu	Vt3	0.9651560	0.6510844
D4	Ambu	Vt3	0.9665013	0.6799636
E1	Ambu	Vt3	0.9700687	0.7563251
E2	Ambu	Vt3	0.9513170	0.3875243
A5	BBVM	Vt3	0.8899231	0.0268047
A6	BBVM	Vt3	0.9275672	0.1386234
B1	BBVM	Vt3	0.9712660	0.7813621
B2	BBVM	Vt3	0.9837062	0.9725765
B3	BBVM	Vt3	0.9720668	0.7977934
B4	BBVM	Vt3	0.9490043	0.3522290
B5	BBVM	Vt3	0.9501684	0.3696530
B6	BBVM	Vt3	0.8995833	0.0405039
C1	BBVM	Vt3	0.9568823	0.4835985
C2	BBVM	Vt3	0.9709763	0.7753499
C3	BBVM	Vt3	0.9821394	0.9586349
D1	BBVM	Vt3	0.9893206	0.9972101
D2	BBVM	Vt3	0.9588289	0.5207275
D4	BBVM	Vt3	0.9587685	0.5195503
E1	BBVM	Vt3	0.9055506	0.0524658
E2	BBVM	Vt3	0.9415756	0.2567563

- **Visualizing the differences between the BVM Types per each participant**

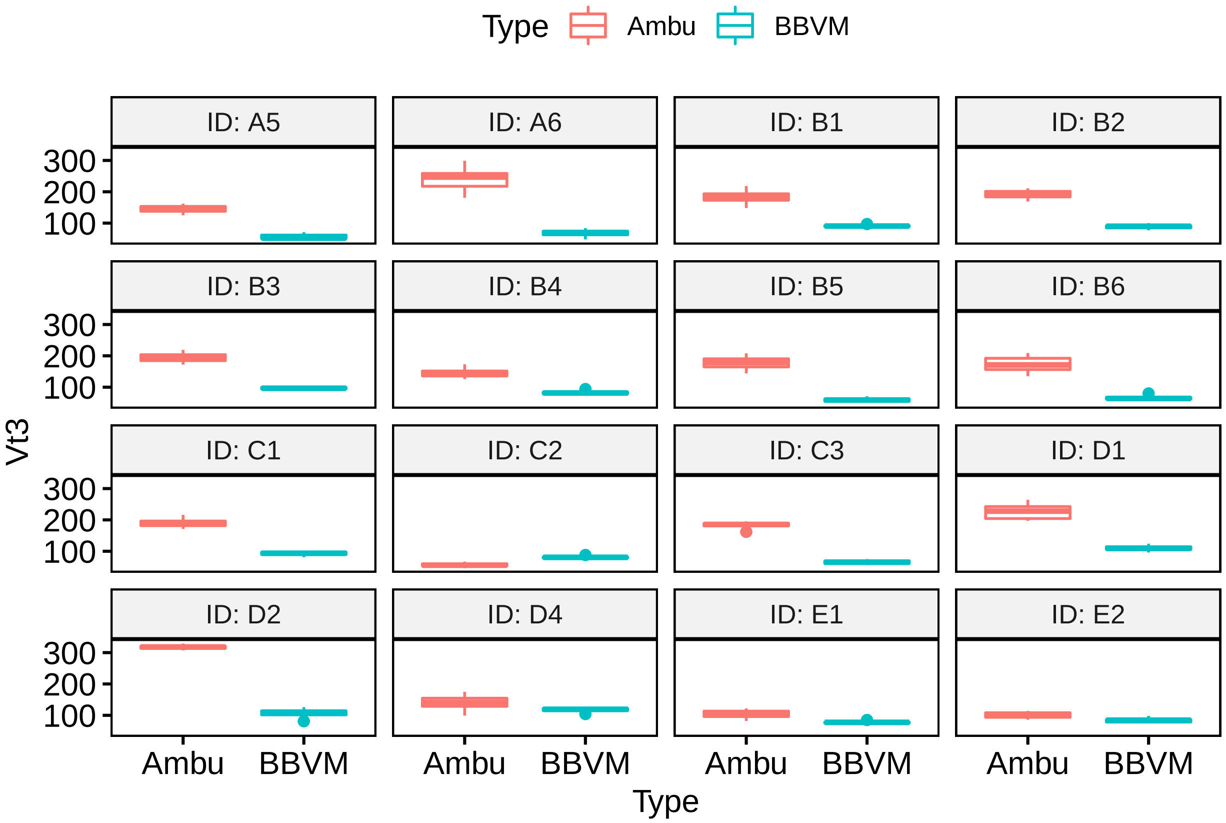

- **Pairwise T-test comparing the BVM Types by each participant**

ID	.y.	group1	group2	n1	n2	statistic	df	p
A5	Vt3	Ambu	BBVM	20	20	40.927950	19	0.000000
A6	Vt3	Ambu	BBVM	20	20	21.656285	19	0.000000
B1	Vt3	Ambu	BBVM	20	20	20.605894	19	0.000000
B2	Vt3	Ambu	BBVM	20	20	32.236589	19	0.000000
B3	Vt3	Ambu	BBVM	20	20	27.670174	19	0.000000
B4	Vt3	Ambu	BBVM	20	20	29.197424	19	0.000000
B5	Vt3	Ambu	BBVM	20	20	33.401089	19	0.000000
B6	Vt3	Ambu	BBVM	20	20	23.844124	19	0.000000
C1	Vt3	Ambu	BBVM	20	20	35.840700	19	0.000000
C2	Vt3	Ambu	BBVM	20	20	-16.862858	19	0.000000
C3	Vt3	Ambu	BBVM	20	20	52.077448	19	0.000000
D1	Vt3	Ambu	BBVM	20	20	23.706873	19	0.000000
D2	Vt3	Ambu	BBVM	20	20	71.813142	19	0.000000
D4	Vt3	Ambu	BBVM	20	20	4.389202	19	0.000315
E1	Vt3	Ambu	BBVM	20	20	11.096210	19	0.000000
E2	Vt3	Ambu	BBVM	20	20	5.827499	19	0.000013

ANOVA approach for the comparisons

- **"Interaction" plot between the Type and the repeated measurements, Trial, on Vt**

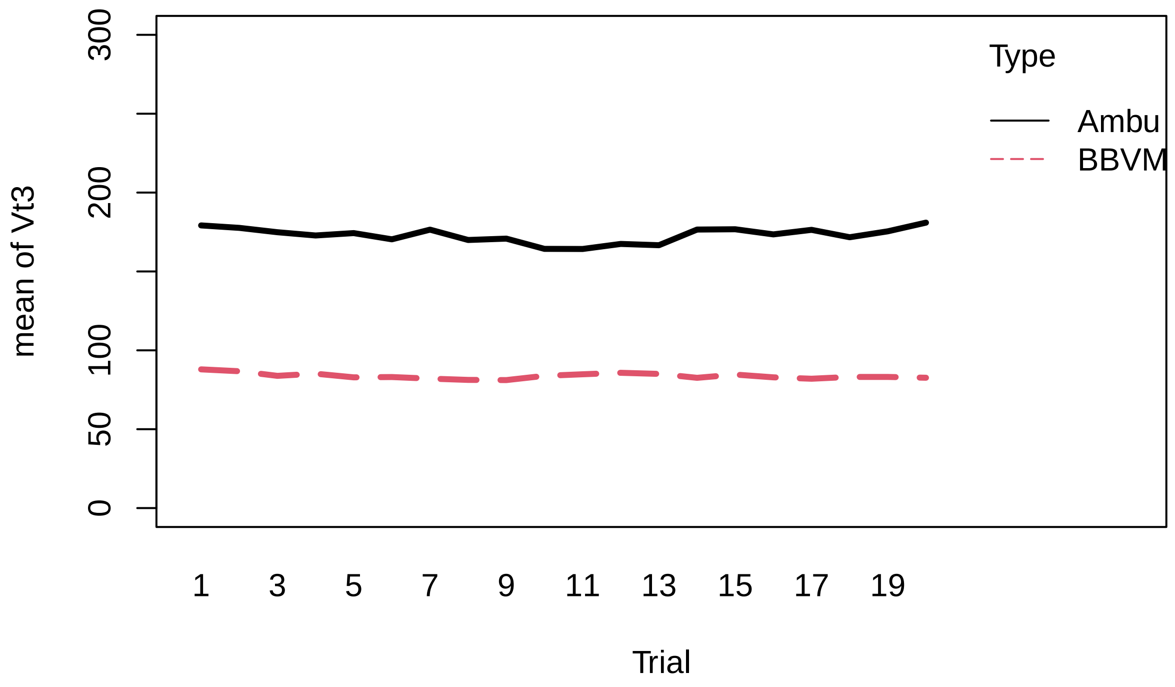

- **With Type only and also accounting for the random effects of the Participants.**

```
##
## Call:
## aov(formula = Vt3 ~ Type + Error(ID), data = data0)
##
## Grand Mean: 128.3687
##
## Stratum 1: ID
##
## Terms:
##                  Residuals
## Sum of Squares    696426.3
## Deg. of Freedom         15
##
## Residual standard error: 215.4725
##
## Stratum 2: Within
##
```

```
## Terms:
##                       Type Residuals
## Sum of Squares  1275739.8  613772.9
## Deg. of Freedom         1       623
##
## Residual standard error: 31.38772
## Estimated effects are balanced
```

```
##           Df Sum Sq Mean Sq F value Pr(>F)
## Residuals 15 696426   46428
```

```
##            Df  Sum Sq Mean Sq F value Pr(>F)
## Type        1 1275740 1275740    1295 <2e-16 ***
## Residuals 623  613773     985
## ---
## Signif. codes:  0 '***' 0.001 '**' 0.01 '*' 0.05 '.' 0.1 ' ' 1
```

- **With Type and Exp and also accounting for the random effects of the Participants (unbalanced case).**

```
##
## Call:
## aov(formula = Vt3 ~ Type + Exp + Error(ID), data = data0)
##
## Grand Mean: 128.3687
##
## Stratum 1: ID
##
## Terms:
##                       Exp Residuals
## Sum of Squares    20852.4  675573.8
## Deg. of Freedom         2        13
##
## Residual standard error: 227.9632
## Estimated effects may be unbalanced
##
## Stratum 2: Within
##
## Terms:
##                       Type Residuals
## Sum of Squares  1275739.8  613772.9
## Deg. of Freedom         1       623
##
## Residual standard error: 31.38772
## Estimated effects are balanced
```

```
##           Df Sum Sq Mean Sq F value Pr(>F)
## Exp        2  20852   10426   0.201  0.821
## Residuals 13 675574   51967
```

```
##            Df  Sum Sq Mean Sq F value Pr(>F)
## Type        1 1275740 1275740    1295 <2e-16 ***
```

```
## Residuals 623  613773     985
## ---
## Signif. codes:  0 '***' 0.001 '**' 0.01 '*' 0.05 '.' 0.1 ' ' 1
```

- **With Type and Gender also accounting for the random effects of the Participants (unbalanced case).**

```
##
## Call:
## aov(formula = Vt3 ~ Type + Gender + Error(ID), data = data0)
##
## Grand Mean: 128.3687
##
## Stratum 1: ID
##
## Terms:
##                   Gender Residuals
## Sum of Squares      54.6  696371.7
## Deg. of Freedom        1        14
##
## Residual standard error: 223.0265
## Estimated effects are balanced
##
## Stratum 2: Within
##
## Terms:
##                      Type Residuals
## Sum of Squares  1275739.8  613772.9
## Deg. of Freedom         1       623
##
## Residual standard error: 31.38772
## Estimated effects are balanced
```

```
##           Df Sum Sq Mean Sq F value Pr(>F)
## Gender     1     55      55   0.001  0.974
## Residuals 14 696372   49741
```

```
##            Df  Sum Sq Mean Sq F value Pr(>F)
## Type        1 1275740 1275740    1295 <2e-16 ***
## Residuals 623  613773     985
## ---
## Signif. codes:  0 '***' 0.001 '**' 0.01 '*' 0.05 '.' 0.1 ' ' 1
```

• **With Type, Exp and Gender also accounting for the random effects of the Participants (unbalanced case).**

```
##
## Call:
## aov(formula = Vt3 ~ Type + Gender + Exp + Error(ID), data = data0)
##
## Grand Mean: 128.3687
##
## Stratum 1: ID
##
## Terms:
##                    Gender      Exp Residuals
## Sum of Squares      54.6  22324.2  674047.4
## Deg. of Freedom        1        2        12
##
## Residual standard error: 237.0034
## Estimated effects may be unbalanced
##
## Stratum 2: Within
##
## Terms:
##                       Type Residuals
## Sum of Squares  1275739.8  613772.9
## Deg. of Freedom         1       623
##
## Residual standard error: 31.38772
## Estimated effects are balanced
```

```
##           Df Sum Sq Mean Sq F value Pr(>F)
## Gender     1     55      55   0.001  0.976
## Exp        2  22324   11162   0.199  0.822
## Residuals 12 674047   56171
```

```
##            Df  Sum Sq Mean Sq F value Pr(>F)
## Type        1 1275740 1275740    1295 <2e-16 ***
## Residuals 623  613773     985
## ---
## Signif. codes:  0 '***' 0.001 '**' 0.01 '*' 0.05 '.' 0.1 ' ' 1
```
